# The unique epicuticular chemistry of Collembola – A cross-species analysis

**DOI:** 10.1016/j.isci.2024.110416

**Published:** 2024-06-28

**Authors:** Anton Möllerke, Gregor Brasse, Jan Bello, Diogo Montes Vidal, Konrad Dettner, Jürg Zettel, Matty P. Berg, Stefan Scheu, Hans Petter Leinaas, Stefan Schulz

**Affiliations:** 1 Technische Universität Braunschweig, Institute of Organic Chemistry, Hagenring 30, 38106 Braunschweig, Germany; 2 Universität Bayreuth, Lehrstuhl für Tierökologie 2, Universitätsstraße 30, 95440 Bayreuth, Germany; 3 Speichergasse 8, 3150 Schwarzenburg, Switzerland; 4 Vrije Universiteit Amsterdam, Institute of Life and Environment, De Boelelaan 1085, 1081 HV Amsterdam, the Netherlands; 5 University of Göttingen, JFB Institute of Zoology and Anthropology, 37073 Göttingen, Germany; 6 University of Göttingen, Centre for Biodiversity and Sustainable Land Use, 37077 Göttingen, Germany; 7 University of Oslo, Department of Bioscience University of Oslo, P.O.Box 1066 Blindern, 0316 Oslo, Norway

**Keywords:** Entomology, Biochemistry, Evolutionary biology

## Abstract

Springtails (Collembola), tiny hexapod arthropods, are abundant in the soil of most ecosystems, but our knowledge of their secondary metabolites is limited, in contrast to that of insects. In insects, the outer cuticle is usually covered by mixtures of long-chain hydrocarbons serving different functions, such as water regulation or chemical communication. In contrast, the knowledge of the epicuticular chemistry of springtails is scarce. We analyzed the cuticular lipids of 23 species covering different lineages. The often complicated structures were elucidated using gas chromatography/mass spectrometry, microderivatization, and synthesis. In contrast to insects, the terpene biosynthetic pathway is used for many of these lipids, producing unprecedented higher terpenes. In addition, evidence for *de novo* cholesterol biosynthesis in springtails was found, which is absent in insects. Finally, diverse non-insect linear compounds originating from the fatty acid biosynthetic pathway were identified. Our comparative analysis showed clear differences compared to insects and shed light on phylogenetic relationships.

## Highlights

• Comparative analysis of epicuticular lipids of 23 springtail species of 8 families
• Epicuticular lipid chemistry of springtails shows clear differences to that of insects
• Discovery of unique higher terpenes, esters, and hydrocarbons as cuticular compounds
• Characterization of many of these lipids by GC/MS, derivatization, and synthesis

## Graphical abstract

**Figure:**
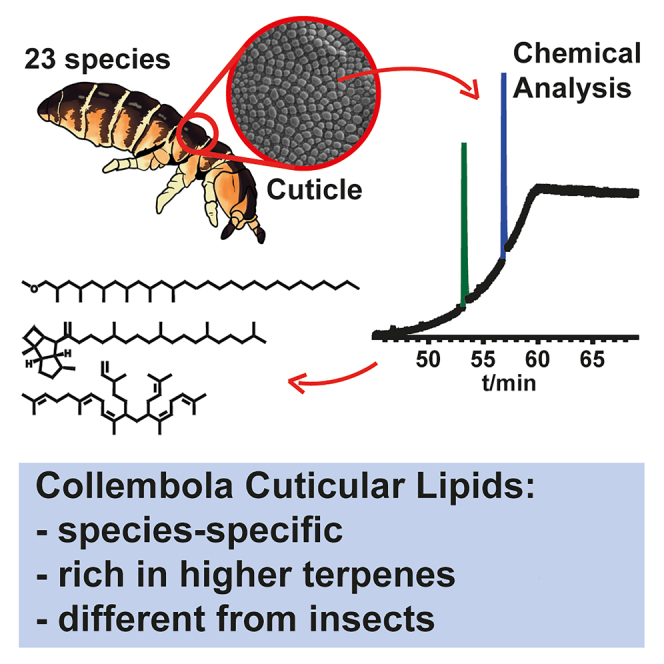

## Teaser

Entomology; Biochemistry; Evolutionary biology; Analytical chemistry; Organic chemistry

## Introduction

Springtails (Collembola) are among the first terrestrial arthropod lineages that appeared about 400 mya.^1^^,^^2^ With about 9,000 recognized species^2^ they are by far the most diverse lineage of basal hexapods that split from a crustacean ancestor together forming the Pancrustacea.^1^^,^^3^ We will compare here springtails especially to insects as more derived hexapods. The phylogenetic relationship of the major lineages of Collembola is still debated, but they include three major species-rich groups: Symphypleona, Poduromorpha, and Entomobryomorpha^4^ with families that also have a very long phylogenetic history.^1^

In contrast to insects which predominantly live above the ground, springtails predominantly colonize soils. Across the globe, springtails form a dominant component of soil animal communities in virtually any ecosystem.^5^^,^^6^ In particular, in high-latitude ecosystems, they reach high abundance, in tundra forests often >100,000 ind/m^2^. They are an important component of the belowground food web being involved in decomposition and mineralization processes with important feedback to plant growth.^7^^,^^8^

An important contribution to the terrestrial success of springtails, insects, and arachnids is the formation of an epicuticular wax layer that reduces water loss.^9^ Respiration is channelized to tracheas in insects and book lounges in spiders. By comparison, springtails except Sminthuridae respire through the main body surface, resulting in a corresponding risk of water loss. To compensate for this, their epicuticle is structured with thinner parts allowing respiration (and water loss) and thicker parts covered by a wax layer that blocks gas exchange. Fine-tuning of the proportion of these two structures allows habitat adaptation. Too much protection will result in reduced respiration and thereby reduced fitness, resulting in a more narrow humidity range compared to insects.^10^^,^^11^ This fine-tuning of the cuticle structure is much less evident in Entomobryidae and Tomoceroidae.^12^

The lipid coating also protects against too much water uptake. The superhydrophobic cuticula of springtails allows some to float on water, while others become covered by a thin air layer (plastron) when submerged in water, allowing respiration and survival when trapped in water-logged soil. This phenomenon depends on the cuticular lipids in combination with the specific physical structure of the cuticle, affecting the contact angle with water.^13^^,^^14^^,^^15^^,^^16^

In addition, epicuticular waxes are involved in chemical communication. This has been extensively documented for insects^17^ and spiders.^18^^,^^19^ but increasing evidence is also accumulating for springtails.^20^^,^^21^ However, the structure of springtail pheromones has been identified only in a few cases.^22^^,^^23^ In insects and spiders, semiochemicals and contact pheromones are known to play an important role in intra- and interspecies recognition.^17^^,^^24^ This may well also be the case for springtails.

Our study investigates how epicuticular lipids and their likely biosynthetic pathways may reflect evolutionary differences in adaptations to terrestrial conditions. Specifically, we ask if there are characteristics of the springtail wax layer that indicate a unique evolutionary event, separated from that of the insects, and if there are systematic differences among the major lineages of springtails.

For these questions, the wax lipids involved in the formation of waterproof coatings are of particular interest. They represent the result of an ancient expansion into the terrestrial environment. In principle, all springtails of different phylogeny and drought exposure could have settled with the same lipid structure, only varying in amount and spatial distribution on the cuticle. However, recent results suggest that a multitude of events during this long terrestrial period have involved both selective and non-selective (e.g., via genetic drift) changes in the compound structure. An important question is whether such changes have occurred sufficiently rare to reveal phylogenetic patterns.

By contrast, the compounds involved in chemical communication reflect continuously changing selections for emission and perception of chemical information. Such changes may occur fairly quickly, even between populations of the same species, and thus complicate its explanatory power of phylogeny, in particular concerning the interpretation of relationships between higher taxa.^25^

The cuticular chemistry of insects is well understood. Their epicuticular wax layer usually consists of a complex mixture of fatty acid-derived compounds,^26^^,^^27^^,^^28^ mainly *n*- and methyl-branched long-chain alkanes and alkenes. Still, it may also contain other components such as long-chain esters, aldehydes, alcohols, or dialkyltetrahydrofurans.^17^^,^^29^^,^^30^ Similar compounds can be found in the phylogenetically more distant spiders (Arachnida) that also use hydrocarbons, but in addition often methyl ethers and esters,^18^^,^^31^^,^^32^ again all biosynthesized via the fatty acid pathway.

Despite their global distribution and high local abundance, our knowledge of the cuticular chemistry of springtails is limited. There have been isolated studies on cuticular lipids^15^^,^^33^ and some structures of individual compounds have been reported.^34^^,^^35^^,^^36^ These limited studies showed that the cuticular chemistry of springtails is likely different from that of insects. Springtails seem to use only a few compounds rather than complex mixtures found in insects and recruit long terpenes with more than six isoprene units and unusual structures, for example, poduran (**18**)^34^ or viaticene (**8**).^35^ However, long-chain alkanes likely formed via the fatty acid biosynthetic pathway are also used, e.g., sarekensane (**1**) containing cyclopropane units not found in other arthropod cuticular lipids.^36^

Some major difficulties in the analysis of springtails are their small body size and difficult taxonomy. Cultivation in the laboratory is tedious and time-consuming, while collection in the wild requires correct species determination on an individual basis, if not mass occurrences can be found. Therefore, gas chromatography (GC) coupled with mass spectrometry (MS) serves as the primary method for the analysis of cuticular lipids. In contrast to insects, in which well-developed mass spectral analysis methods allow relatively easy determination of structures of alkanes,^26^^,^^37^ this is not the case with many of the compounds we encountered during the analysis of a wide variety of lipids from different springtail lineages. Often only a few compounds occurred in the cuticular extracts, different from the typical insect mixtures, showing unusual structures. In some cases, mass occurrences allowed the isolation of enough material for NMR-guided structure determination of an isolated sample.^34^^,^^35^^,^^36^ In other cases, the characterization was performed by interpretation of mass spectra, gas chromatographic retention indices (*I*), microderivatization of extracts, and GC/infrared spectroscopy (GC/IR) measurements.

During our analysis, we found many cuticular compounds that showed marked structural differences from those found in other arthropods. Here, we aim to provide an overview of the cuticular chemistry of springtails to delineate more general patterns of springtail cuticular lipid chemistry. For this analysis, we collected data from species representing the three major lineages of springtails: Poduromorpha, Entomobryomorpha, and Symphypleona. In total, we analyzed 23 species from eight families. We present here the results of our analysis, which may vary in depth from species to species depending on the number of individuals available to us. We could observe a trend toward unique structures, quite opposite to the cuticular chemistry of insects.

## Results

Springtails were either collected in the wild or cultured in the laboratory to increase biomass. For analysis by GC/MS, extracts of whole animals were prepared by short-term extraction in pentane or CH_2_Cl_2_. Although pentane is known to preferentially extract epicuticular lipids, in some cases with low biomass CH_2_Cl_2_ was used, which extracts additionally more internal compounds, allowing the extract to be used also for studies outside the scope presented here. From the various analytical data, sound structural proposals were made, sometimes confirmed by chemical synthesis. In some cases of novel compounds, structural characterization was performed only up to the compound class level. This was possible due to typical mass spectral features. However, complete structure elucidation will have to wait until more material is available or until a usually time-consuming synthesis^38^ confirms the structure.

**Figure 1:**
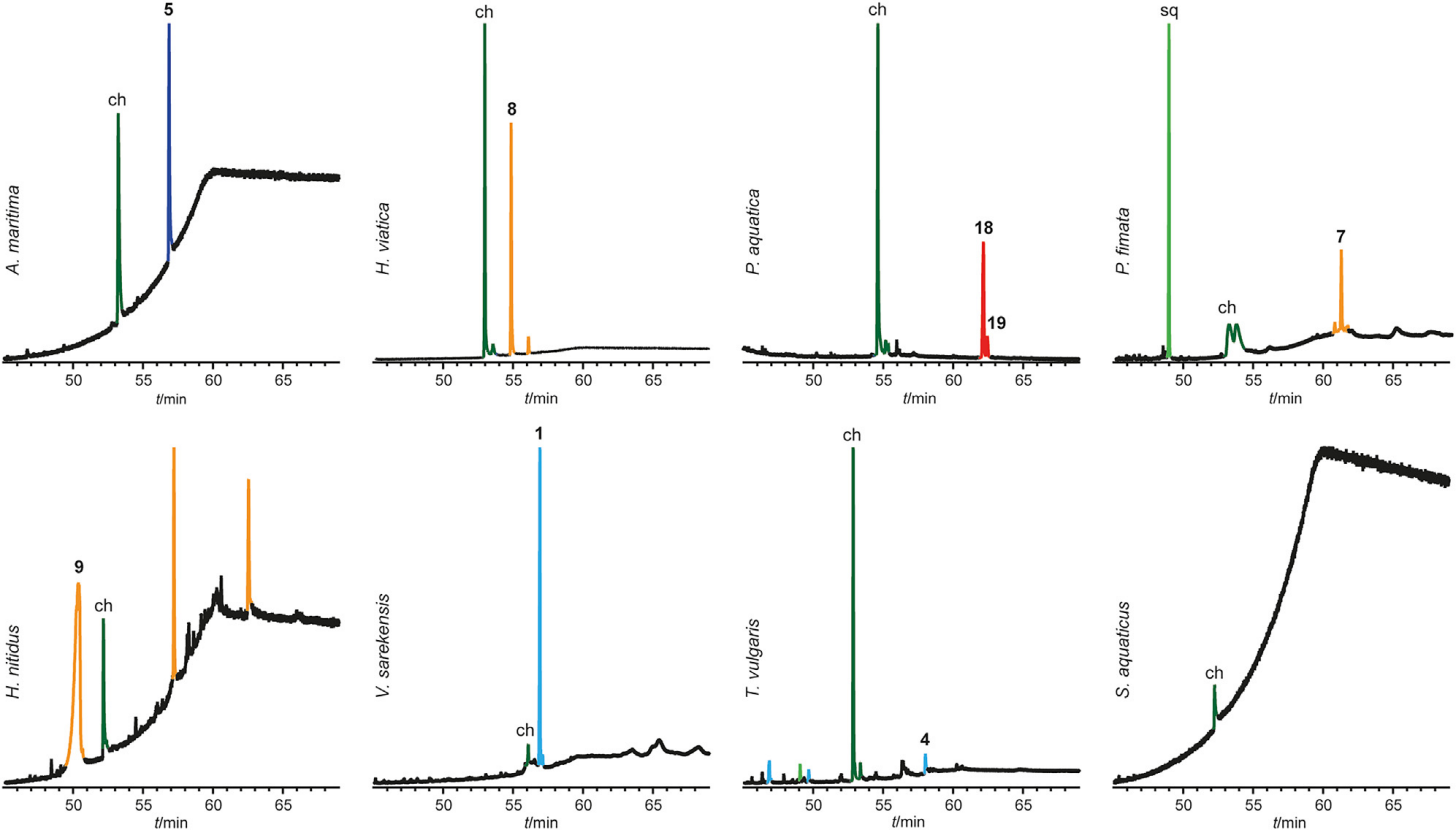
Representative total ion chromatograms of various springtails of different families and their structural compound classes . . . . . .

Unlike insect cuticular extracts the springtail cuticular extracts contained only a few compounds. Most species showed less than five major components, of which cholesterol (ch) and squalene (sq) were present in almost all species. The results of the analysis of the 23 species from the eight families Neanuridae, Hypogastruridae, Poduridae, Isotomidae, Entomobryidae, Tomoceroidae, Onychiuridae, and Sminthurididae are shown in Table S1 in the SI. Representative total ion chromatograms can be found in Figure 1 and the SI. Free fatty acids and glycerides were not further characterized as they were likely internal compounds derived from body fat and varied highly between samples. Compounds with less than 20 carbons were also excluded, as their major biological function is likely to serve as volatile signals or as defense compounds. The data of *Tetrodontophora bielanensis* from Stránský et al.,^33^ who provided a detailed analysis of their lipid composition, were also added. Diterpenes with 20 carbons were included here for completeness, although their function as lipids is in question, as they usually serve different functions in arthropods.^39^^,^^40^

The results showed some general trends. Cholesterol and squalene were ubiquitous. Desmosterol is widely distributed within Poduromorpha (in seven out of 13 species), while it was only detected in one other species (*T. vulgaris*, Entomobryomorpha). The abundance of lycopene derivatives showed a similar trend, being detected in four Poduromorpha species, but not in species of other groups. In the following sections, we will discuss the other components of the different families in detail.

### Neanuridae

*Anurida maritima* was the only species from the family Neanuridae. The lipids contained only ch and compound **ANM-A** (M^+^ *m/z* 564) as major epicuticular compounds (Figure S1). Analysis of the mass spectrum of **ANM-A** and transesterification of the extract indicated this compound to be a long-chain carboxylic ester, 2,4,6-trimethylhexadecyl 2,4,6-trimethylhexadecanoate (**5**). An enantioselective synthesis using a procedure developed by Feringa et al.^41^^,^^42^^,^^43^ proved the structure. It allowed the determination of the absolute configuration of this compound with six stereogenic centers (see STAR methods). All show *S*-configuration. Two minor analogs were identified as 2,4,6-trimethyltridecyl 2,4,6- and 2,4,6-trimethyltetradecyl 2,4,6-trimethylhexadecanoates (see STAR methods). This type of lipid, as a multi-methyl-branched ester, does not occur in any of the other springtails investigated.

### Hypogastruridae and Poduridae

**Figure 2:**
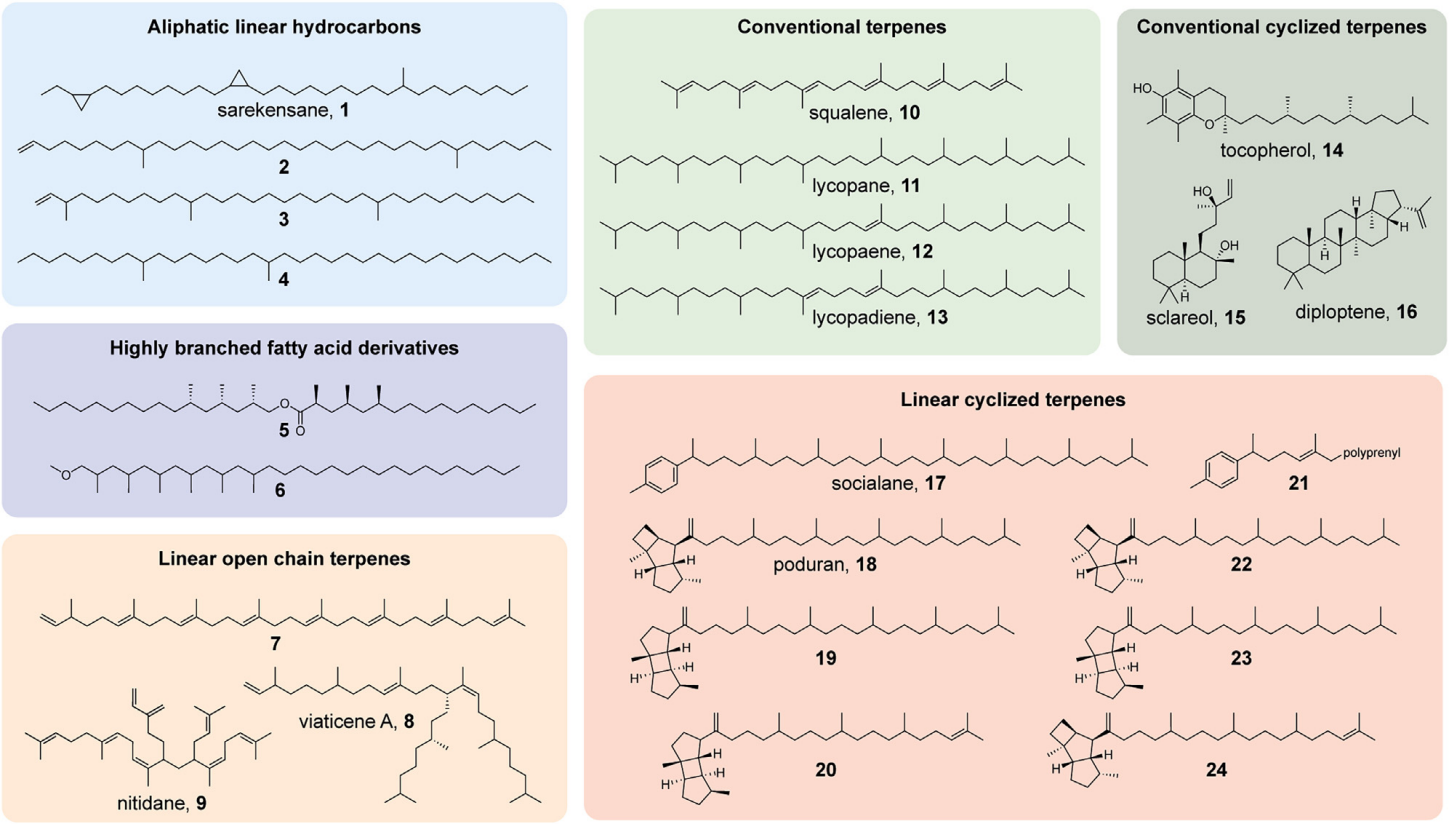
Structures of cuticular compounds of the investigated springtails The compounds are classified into different classes according to their proposed biosynthesis. The color code is used in this manuscript in all figures.

Within the Hypogastruridae, seven species of the three genera *Ceratophysella, Hypogastrura,* and *Xenylla* were analyzed. The lipid profiles of *Ceratophysella denticulata* and *sigillata* are very similar (Figures S2 and S3). Both *C. denticulata* and *C. sigillata* show a combination of regular highly saturated [4^1^+4^1^] tetraterpenes,^35^ namely lycopane (**11**), lycopaene (**12**), and lycopadiene (**13**) (Figure 2). The position of the double bonds was determined by dimethyl disulfide addition and MS analysis (see STAR methods). In addition, both species contain the steroids ch and desmosterol, and *C. sigillata* additionally cholestanol. Interestingly, the triterpene hopanoid diploptene (**16**) occurs in small amounts.

The three *Hypogastrura* species (Figures S4–S6) also show some similarities but differ in lipid composition from *Ceratophysella*. While again different steroids occur, specific unusual terpenes dominate. *Hypogastrura viatica*^35^ and *Hypogastrura vernalis* contain the unique [6^14^+2^1^]-terpene viaticene A (**8**) as a major compound, accompanied by viaticene B. Viaticenes are regularly connected triterpenes that are geranylated at C-14, leading to a unique Y-shape structure not reported from other natural sources. In contrast, *Hypogastrura socialis* contains a regularly head-to-tail linked [9]-terpene, socialane (**17**),^38^ as the major epicuticular compound. In addition, especially *H. socialis*, but also *H. vernalis* show minor amounts of some odd-numbered linear alkanes ranging from C_21_ to C_31_. Nevertheless, no branched alkanes occur, compounds usually found in insect cuticular alkanes.

Before discussing the genus *Xenylla*, we will switch to *Podura aquatica* first, which is the only podurid species included here, because the major lipid is the unique [8]-terpene poduran (**18**)^34^ (Figure S9). This compound defines a new lipid type, a long saturated terpenoid alkyl chain carrying a typical sesquiterpene-like head group. It is accompanied by the related decahydropentaprenylprespatane (**19**)^44^ in a 5:1 ratio. The structure of **19** was confirmed by its mass spectrum and synthesis of ozonolysis degradation products (STAR methods). A similar structural principle is found in **17**. Minor amounts of steroids were also detected.

The two *Xenylla* species contain related long isoprenyl compounds with cyclic head groups. *Xenylla grisea* (Figure S7) showed a compound whose mass spectrum (Figure S30) is highly similar to that of poduran (**18**),^34^ but has an M^+^ ion 70 amu lower. This indicated one saturated isoprene unit less in the side chain, making it a [7]-terpene, octahydrotetraprenylkelsoene (**22**), compared to the [8]-terpene **18**. Similarly, the shorter variant of **19**, octahydrotetraprenylprespatane (**23**) occurred, as well as two hexahydrotetraprenyl analogs (**20**, **24**) and two unknown [7]-terpenes (Figure S31) of similar overall structure with an unidentified head group, **XGR-C** and **F** (see STAR methods).

*X. maritima* (Figure S8) was collected in the wild and occurred together with *Anurophorus laricis.* The two species were carefully separated before extraction. The major lipid **XEM-B** was an unknown [8]-terpene (M^+^ *m/z* 552) (Figure S32). The structure could not be elucidated, but the mass spectrum (Figure S31) indicated a similar head/tail rearrangement as in the other *Xenylla* compounds (see STAR methods). *X. maritima* additionally contains 1-methoxy-2,4,6,8,10,12-hexamethylnonacosane (**6**) as a main compound, whereas in *X. grisea* only small amounts of *n*-alkanes (C_28_–C_30_) were detected as additional components of the cuticular lipids. The structure of **6**, the first polymethyl-homovicinal-branched lipid in arthropods resembling respective acids of *Mycobacterium tuberculosis* such as phthioceranic acid,^43^ was deduced from the mass spectrum (Figure S33). The ion *m/z* 45 indicates the presence of a methyl ether group, a compound class reported earlier from various spider cuticular and web lipids.^18^^,^^45^ The methyl branching pattern was deduced from the spectra of related compounds (for details, see STAR methods).

### Onychuiridae

The onychiurid species analyzed include *Protaphorura fimata*, *Onychiurus fimatus*, and *Megaphorura arctica* (Figures S10–S12). All of them contain ch and desmosterol. The tetraterpenes **11**–**13** were reported from *Tetrodontophora bienalensis* earlier.^15^^,^^33^ A major tetraterpene (**PFI-C**) showed a mass similar to that of lycopersene but with distinct differences. A detailed analysis (see STAR methods) showed that this compound is most likely a [8]-terpene, different from the [4^1^+4^1^]-connectivity found in conventional tetraterpenes such as **11**–**13**. The mass spectral data indicated the tentative structure **7**. Some minor related compounds differing in the number of double-bonds accompanied **PFI-C**. Compound **7** also occurred in *O. fimatus*.

In *M. arctica* only tocopherol (**14**) was detected besides the steroids and sq. An older study of whole-body extracts of *Tetrodontophora bielanensis* reported several alkanes (C_15_–C_35_, C_40_–C_51_) and a secondary alcohol (C_40_H_82_O) although the cuticular composition remained unclear.^30^ In *M. arctica, O. fimatus,* and *P. fimata,* no straight-chain hydrocarbons were detected.

### Isotomidae

Within the Isotomidae five species were analyzed, *Anurophorus laricis, Folsomia quadrioculata*, *F. candida, Vertagopus sarekensis*, and *Cryptopygus clavatus.* The extracts of *A. laricis* showed ch and cholest-3,5-diene, but no higher terpenes (Figure S13). A straight-chain hydrocarbon occurred, likely hentetracosadiene (see STAR methods). In contrast, two structurally similar unsaturated tetraterpenes **FQU-E/F** were present as the major epicuticular lipids in *F. quadrioculata* (Figure S14). The mass spectrum of **FQU-E** was similar in overall appearance and some ions with branched terpenes such as **8** and nitidane (**9**). The low amount of material available did not allow full structural identification but suggested an isoprenyl-branched [8]-terpene structure. In addition, the diterpene sclareol (**15**), some unknown terpenoids, and a long-chain aliphatic compound occurred. Finally, a late-eluting compound was tentatively identified as a hexatriacontatetraene.

Several terpenes, including [7]- and [8]-terpenes were detected in the extracts of *F. candida* (Figure S15). The mass spectra of **FC-B**–**FC-D** (Figure S45) indicate partly unsaturated linear terpene analogs of socialane (**17**) with a tolyl head group (**21**) (see STAR methods for more details). Several other unknown compounds occurred as well.

The pentane extracts of *Vertagopus sarekensis* (Figure S16) were dominated by the recently described unusual long-chain dicyclopropane hydrocarbon sarekensane (**1**).^36^ Trace constituents are likely long-chain alkenes (C_23_–C_32_), but since the mass spectra of dialkylcyclopropanes are indistinguishable from those of the corresponding alkenes,^36^ they may also be additional cyclopropanes. *C. clavatus* showed tocopherol (**14**) and cholest-7-en-3-ol, as well as several unknown long-chain fa-derived compounds (Figure S17).

### Entomobryidae and Sminthurididae

Three species of Entomobryidae were analyzed: *Heteromurus nitidus, Orchesella cincta,* and *Sinella curviseta*. The epicuticle of *H. nitidus* (Figure S18) is preferentially formed by the unique, highly branched nitidane (9),^46^ accompanied by two other highly branched open-chain terpenes. **HEN-B** is likely a [8]-terpene with nine double bond equivalents (Figure S48) and **HEN-C** (Figure S49) is the corresponding [9]-terpene. In addition, a diterpene (C_20_H_30_O) is present in the extract. In *Orchesella cincta,* no terpenes were detected besides ch. The epicuticular extract was dominated by methyl-branched alkenes (Figure S19), 9,29-dimethylpentatriacont-1-ene (**2**), and 3,11,23-trimethyltritriacont-1-ene (**3**). *S. curviseta* extracts contained an unknown diterpene and a polymethyl-branched hexatriacontahexaene (**SIC-A**) (Figures S20 and S53).

The only species of the Tomoceroidae, *Tomocerus vulgaris*, did not contain any epicuticular terpenes besides ch and **10** (Figure S21). Several long-chain alkenes, heptacosadiene, heptacosene, and nonacosene, as well as 9,17-dimethylpentatriacontane (**4**) were identified. The structures were deduced from the mass spectra (Figure S55).

*Sminthurides aquaticus* (Sminthurididae) was the only Symphypleona analyzed. The extracts of this species did not contain any terpenes apart from ch and showed small amounts of two acetates only, hexadecyl and octadecyl acetate (Figure S22).

## Discussion

Many of the springtail lipids discussed here are unique, not found in other organisms, and comprise a wide range of compound structures. They can be divided into six different compound classes according to their structure and their likely biosynthetic origin, as shown in Figure 2. Aliphatic linear hydrocarbons known from other arthropods are present but rarely abundant. Highly poly-β-methyl branched esters and ethers originate from the fatty acid biosynthetic pathway. These highly β-branched fatty acid derivatives, containing oxygen, were identified here for the first time as arthropod lipids. Regular tri- and tetraterpenes, termed here conventional terpenes because of their typical [3^1^+3^1^]- and [4^1^+4^1^]-linkage, are common, as are most conventional cyclized terpenes, which include steroids but also other compounds. Of special interest are regularly connected terpenes, which are present either as linear open-chain terpenes that often include branching through prenylation, or constitute linear cyclized terpenes that show a typical cyclized head group with a long aliphatic chain. Such compounds are very rare in nature. A few examples are [6]- and [7]-terpenes found in bacteria^47^^,^^48^^,^^49^^,^^50^ and prenylated [3^6^+2^1^]- and [3^6^+3^1^]-terpenes from diatoms.^51^^,^^52^ In summary, our results indicate a complex, abundant terpene and cuticular lipid biosynthesis in springtails, often different from those of other organisms.

**Figure 3:**
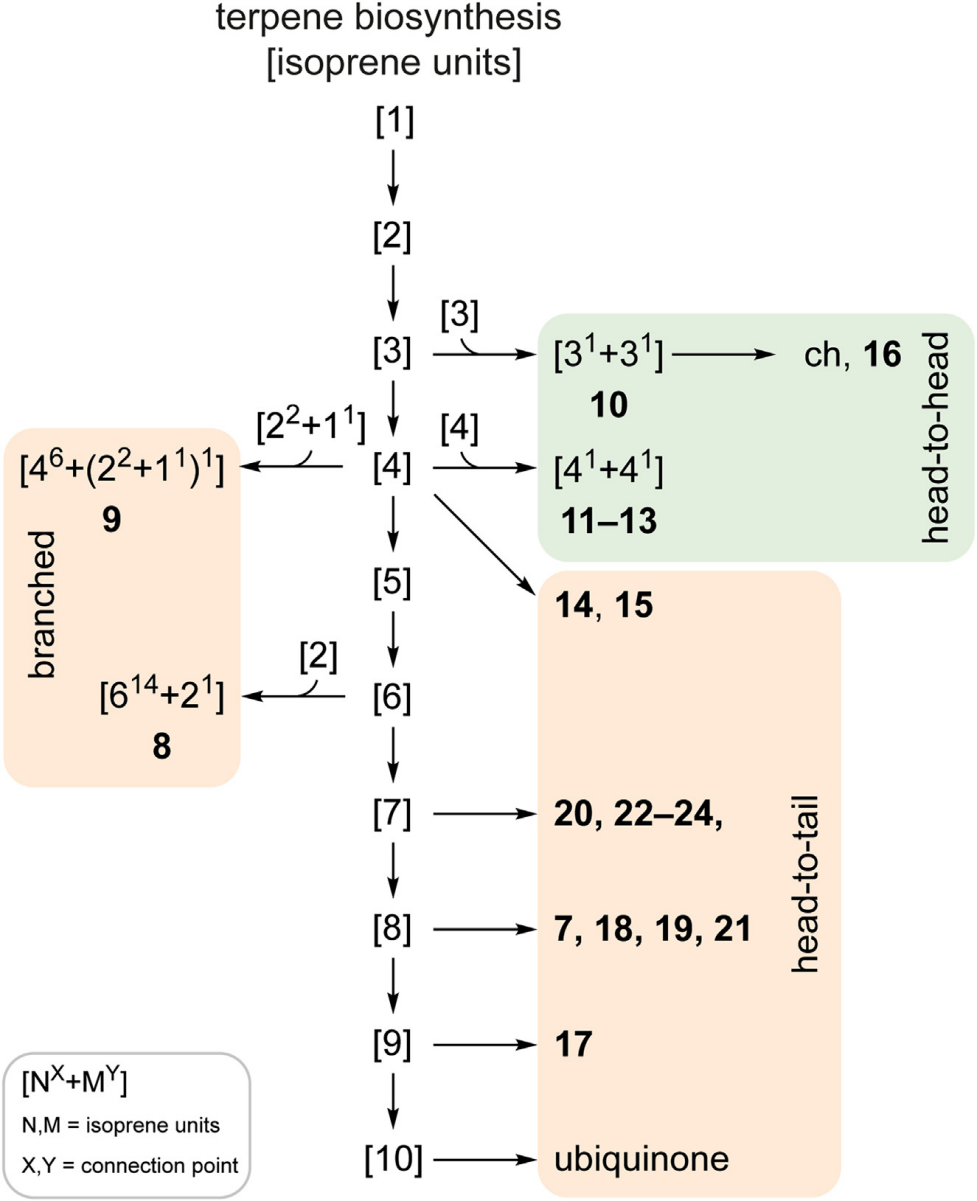
Proposed origin of the springtail cuticular terpenes from the ubiquinone biosynthetic pathway Common terpene biosynthesis to smaller terpenes and sterols follows the green pathway in most organisms, while springtails also exploit the ubiquinone pathway. Numbers in square brackets denote the number of isoprene units present in the respective pyrophosphates, the terpene building blocks.

Our data show important differences in the cuticular chemistry of springtails compared to insects, but also to the less studied, phylogenetically distant, arachnids. While in insects complex mixtures of hydrocarbons are common, all springtails investigated showed a much-reduced composition, typically representing only up to two or three major components (Figure 1, Table S1). Arachnids also use complex mixtures of hydrocarbons,^53^^,^^54^^,^^55^ alkyl methyl ethers, or esters,^18^^,^^55^^,^^56^^,^^57^ however, in some species single or few compounds occur.^24^^,^^58^ All these compounds are biosynthesized via the fatty acid biosynthetic pathway.^59^^,^^60^ The epicuticular waxes of insects can contain >90% hydrocarbons, while higher terpenes (more than 20 C) except sq and ch are rare.^61^ In contrast, the use of higher terpenes, e.g., [7]–[9]-terpenes, is widespread in springtails; 16 out of 23 analyzed species showed such compounds (Figure 3), including linear, prenylated structures, or compounds with a cyclic head group. In most cases, aliphatic linear hydrocarbons present in insects were detected in minor amounts, only. The abundance of fa-derived compounds in springtails is reduced, although dominating in some taxa. However, in such species structurally unique compounds not reported from insects or spiders were used, such as the polymethyl-branched ether **6** or the ester **5**, both characterized here for the first time. Even methyl-branched alkanes such as **1**–**4** are different from insect hydrocarbons that show often a 1,5- or 1,7-arrangement of methyl groups, while in springtails the methyl groups a wider apart from each other. The latter can also be observed in methyl ethers of arachnids.^24^^,^^45^ The dicyclopropylalkane motif of **1** is not reported from other organisms. Furthermore, all higher terpenes and hydrocarbons of the springtails are somewhat larger, 35–45 carbons, compared to pterygote insects that usually preferentially contain between 25 and 35 carbons. This may be due to the larger surface-to-body area of the small springtails compared to insects, making prevention of water loss more important. The higher carbon number and the reduced mixtures indicate a lower permeability of water of the springtail lipid layer compared to insects. Furthermore, the often highly branched chain might reduce viscosity of the lipid layer in the springtail lipids compared to hydrocarbon mixtures.

**Figure 4:**
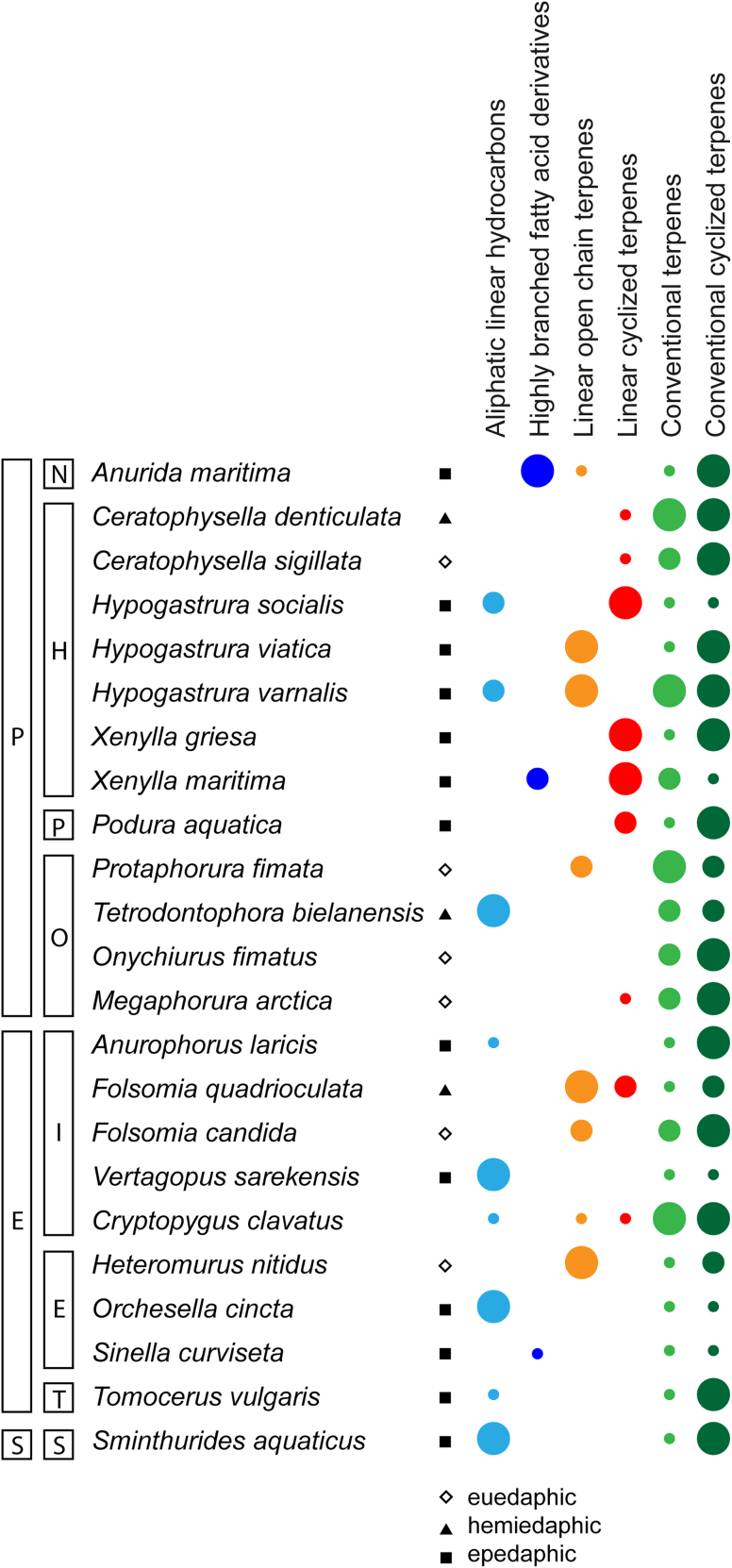
Compound classes and relative abundance in the analyzed springtail species The blue compounds are formed via the fatty acid biosynthetic pathway. The green compounds are products of the conventional tri- and tetraterpene biosynthesis. The orange/red compounds include linear terpenes, their cyclization products and prenylated compounds with linear head-to-tail connection, likely recruited from the ubiquinone pathway. P, Poduromorpha; N, Neanuriadae; H, Hypogastruridae; P, Poduridae; O, Onychiuridae; E, Entomobryomorpha; I, Isotomidae; E, Entomobryidae; T, Tomoceridae; S, Symplypleona,S, Sminthuridae.

Although insects and other protostomes were believed not to be able to biosynthesize triterpenes, tetraterpenes, and sterols, with some exceptions like aphids,^62^ lacking the required biosynthesis genes,^63^^,^^64^^,^^65^ both the triterpene squalene (**10**), a [3^1^+3^1^]-terpene, as well as cholesterol were present in almost all species (Figure 4). Laboratory colonies fed exclusively yeast showed cholesterol, but not the yeast ergosterol. Therefore, we looked into springtail genomes in the NCBI database for cholesterol biosynthetic genes (Figure S56; Table 1). All biosynthetic genes of the cholesterol pathway were present in the genomes of *F. candida*, *O. cincta*, and *Allacama fusca* (see STAR methods), except squalene monooxygenase (SQLE) and cholestenol delta-isomerase (EBP). Furthermore, squalene synthase (SQS) and lanosterol synthase (LSS) were found only in *F. candida*. These data strongly suggest that springtails can synthesize at least triterpenes and sterols *de novo*. Furthermore, a putative squalene-hopene cyclase might explain the occurrence of hopanoids such as diploptene (**16**) in some species. However, uptake with food^66^ or production by symbiotic microorganisms might also be possible.

**Table 1:** Genes related to cholesterol biosynthesis and the springtail species in which genomes they were found

Name	Description	Accession No.	Found in the genome of (Sequence ID; E value; percentage identity)
SQS	farnesyl-diphosphate farnesyltransferase	NP_001274671	*F. candida* (NW_019091197; 3e-58; 34.64%)
SQLE	squalene monooxygenase	NP_003120	
LSS	lanosterol synthase	NP_001001438	*F. candida* (XP_035701421; 8e-41; 25.08%)
CYP51A1	sterol-14 α demethylase	NP_000777	*F. candida* (OXA62685.1; 5e-24; 25.00%)*, O. cincta* (ODM89791.1; 6e-28; 26.08%), *A. fusca* (CAG7726578.1; 2e-24; 33.51%)
TM7SF2	delta14-sterol reductase	NP_003264	*F. candida* (XP_021944966.1; 8e-36; 28.30%), *O. cincta* (ODN02551.1; 4e-40; 31.86%), *A. fusca* (CAG7668699.1; 2e-42; 35.77%)
LBR	lamin-B receptor	XP_011542487.1	*F. candida* (XP_021944966.1; 4e-35; 27.66%)*, O. cincta* (ODM97531.1; 1e-43; 30.77%)*, A. fusca* (CAG7668699.1; 5e-42; 36.15%)
SC4MOL	methylsterol monooxygenase	NP_006736	*F. candida* (XP_021962910.1; 4e-25; 32.55%)*, O. cincta* (ODN01110.1; 2e-09; 27.73%)*, A. fusca* (CAG7821441.1; 7e-05; 24.89%)
NSDHL	3β-chydroxy-4α-carboxy-sterol-3-dehydrogenase	NP_001123237	*F. candida* (CAG7832827.1; 2e-33; 27.84%)*, O. cincta* (CAG7832827.1; 2e-38; 29.78%)*, A. fusca* (CAG7832827.1; 5e-42; 30.98%)
ERG27	3-keto steroid reductase	NP_057455	*F. candida* (XP_021953395.1; 7e-21; 27.09%)*, O. cincta* (ODN02268.1; 5e-19; 29.03%)*, A. fusca* (CAG7654950.1; 3e-13; 25.51%)
CHCR24	Δ24-sterol reductase	NP_055577	*F. candida* (XP_021959020.1; 0; 54.14%)*, O. cincta* (ODM91838.1; 0; 59.31%)*, A. fusca* (CAG7720780.1; 0; 59.92%)
EBP	cholestenol delta-isomerase	NP_006570	
SC5DL	Δ-sterol 5(6)-desaturase	NP_001020127	*F. candida* (XP_021967476.1; 8e-105; 55.15%)*, O. cincta* (ODN01110.1; 5e-123; 54.52%)*, A. fusca* (CAG7821441.1; 1e-62; 54.82%)
DHCR7	7-dehydrocholesterol reductase	NP_001157289	*F. candida* (XP_021944966.1; 1e-50; 33.24%)*, O. cincta* (ODN02551.1; 2e-56; 35.77%)*, A. fusca* (CAG7668699.1; 2e-49; 36.64%)

Linear [7–9]-terpenes are abundantly present in springtails. These metabolites are likely recruited from the biosynthetic pathway leading to ubiquinones that use [9]- or [10]-terpene sidechains with regularly head-to-tail connected prenyl groups (Figure 3).^67^ These ubiquinone pathway terpenes are often further modified leading to cyclized head groups resembling smaller sesquiterpenes (brown in Figure 3) or are prenylated to arrive at highly branched compounds (tan in Figure 3).

The cuticular chemistry of springtails shows some phylogenetic influence. Firstly, there is a relation between taxonomic distance and cuticular lipid composition. *C. denticulata* and *C. sigillata* show a nearly identical lipid profile. The same is true for *H. viatica* and *H. vernalis*, but not for *H. socialis*. Furthermore, the abundance of desmosterol and lycopane derivatives is especially high in species of the order Poduromorpha. There were also several structural motifs shared between different families, e.g., the poduran derivatives in *X. grisea* and *P. aquatica*. These phylogenetic patterns suggest that some epicuticular lipid characteristics may persist over a long evolutionary time. However, several species-specific compounds, such as **5**, **17**, or **3**, suggest that evolution also may occur quicker. For instance, the great difference between *H. socialis* and its two conspecifics indicates the result of distinct selection, perhaps as an adaptation to its advanced behavior and habitat utilization.^68^

Many springtails aggregate in large groups. Several studies imply a major role of chemical signals in this aggregation behavior and in interspecific recognition.^20^^,^^69^^,^^70^^,^^71^ Also, molting^68^ and the reproductive behavior^72^ of springtails may also be controlled by chemical signals. Aggregation also indirectly supports reproductive behavior.^72^ Even so there is exceeding evidence for chemical communication in springtails, we do know little about the chemical composition of these chemical signals.^20^ The long-chain alcohol (*Z*)-14-tricosenol was identified as the sperm-attracting signal of *O. cincta*.^23^ The structural uniqueness of many of the compounds reported here indicates costly specialized enzymes to synthesize the often unique compounds. This might indicate a role in communication because simple desiccation prevention would be achievable through simple compounds such as **10** or simple alkanes. Because mixtures are missing, unique signals can only be achieved by structural variation, while the use of compound mixtures, as often found in insect signaling systems, is not possible. This may indicate the wide structural variability in springtail epicuticular lipids.

These epicuticular lipids may also play an important role in the superhydrophobicity of the springtail cuticle.^73^ The compounds discussed are all highly apolar compounds that may contribute to the wetting characteristics of the springtail epicuticle. Combined with the structural nanopattern of the cuticle the surface becomes strongly water-repellent.^13^^,^^14^ Both components appear to be important, as seasonal changes observed in *C. clavatus* indicate that modification of the lipid layer without affecting the surface nanostructures totally removes the water repellency.^74^

In summary, we identified several new springtail lipids such as **2**–**6**, **11**–**13**, **19**, **21**, and **22**–**24**. Cyclized and branched terpenes dominate, recruited obviously from the ubiquinone biosynthetic pathway. The abundance of sq (**10**) and ch as well as genetic evidence indicate the presence of sterol biosynthesis in springtails, different from insects. The few compounds usually present may influence the water balance and superhydrophobicity of the cuticle, while their structural uniqueness also indicates potential signaling functions. The striking differences between springtails and insects, the former using few highly structurally diverse compounds while the latter relying on complex mixtures of similar hydrocarbons, is likely a functional adaptation to their different lifestyles. Nevertheless, more work is needed on all these aspects to understand the actual role of cuticular compounds in springtails.

### Limitations of the study

This study is based on GC-based approaches, which means that the scope of substances is limited to apolar compounds up to a molecular weight of about 700 Da. Furthermore, in some cases, we were only able to elucidate the substance class of a given compound due to the minute amount of biological material available. Only a selected number of species were available to us for these analyses due to difficulty in obtaining enough material for analysis. The animals originated from central and northern Europe and a wider range of springtails, including e.g., tropical springtails may yield further insights. Furthermore, environmental influences such as season, stress, etc. may lead to changes in the chemical composition, which were not accounted for in this study, as were potential sex and age differences.

## STAR★Methods

### Key resources table

**Table:** 

REAGENT or RESOURCE	SOURCE	IDENTIFIER
**Biological samples**		

*Anurida maritima*	H.P. Leinaas	
*Hypogastrura socialis*	H.P. Leinaas^38^	
*Hypogastrura viatica*	H.P. Leinaas^35^	
*Hypogastrura varnalis*	H.P. Leinaas	
*Ceratophysella denticulata*	S. Scheu	
*Ceratophysella sigillata*	J. Zettel^75^	
*Xenylla grisea*	K. Dettner	
*Xenylla maritima*	H.P. Leinaas	
*Podura aquatica*	K. Dettner^34^	
*Protaphorura fimata*	S. Scheu	
*Onychiurus fimatus*	K. Dettner	
*Megaphorura arctica*	H.P. Leinaas	
*Anurophorus laricis*	H.P. Leinaas	
*Folsomia quadrioculata*	H.P. Leinaas	
*Vertagopus sarekensis*	H.P. Leinaas^36^	
*Cryptopygus clavatus*	H.P. Leinaas	
*Heteromurus nitidus*	S. Scheu	
*Folsomia candida*	S. Scheu	
*Orchesella cincta*	M. Berg	
*Sinella curviseta*	S. Scheu	
*Tomocerus vulgaris*	S. Scheu	
*Sminthurides aquaticus*	S. Scheu	

**Chemicals, peptides, and recombinant proteins**		

mycocerosic alcohol	A. Minaard	CAS: 27829-63-6
Pentane, suprasolv	Merck	CAS: 109-66-0
Dichloromethane, suprasolv	Merck	CAS: 75-09-2
MSTFA	CS-Chromographie	CAS: 24589-78-4
TMSH	TCI	CAS: 17287-03-5

**Deposited data**		

Gas chromatograms of the natural samples	This paper	Shared upon request by the lead contact
Mass spectrometric data of the identified compounds	This paper	Will be published in the open data repository MACE.^76^
NMR data of synthetic products	This paper	Shared upon request by the lead contact

### Resource availability

#### Lead contact

Further information and requests for resources and reagents should be directed to and will be fulfilled by the lead contact, Stefan Schulz (stefan.schulz@tu-bs.de).

#### Materials availability

This study did not generate new unique reagents.

#### Data and code availability

• EI-Mass spectra of all identified compounds will be published in a computer-readable format in the open data repository MACE^76^ in the next release. The current release MACE-r5.1 can be found at https://doi.org/10.24355/dbbs.084-202402071310-0. Any other data reported in this paper will be shared by the lead contact upon request.
• This paper does not report original code.
• Any additional information required to reanalyze the data reported in this paper is available from the lead contact upon request.

### Experimental model and study participant details

*Xenylla grisea* and *Onychiurus fimatus* were collected near Bayreuth, Germany and the latter maintained as a colony. *Hypogastrura vernalis, Xenylla maritima*, *Megaphorura arctica, Anurophorus laricis,* and *Anurida maritima* were collected in Norway mountains and on Norway shores. *Folsomia quadrioculata* and *Cryptopygus clavatus* were maintained as laboratory cultures and fed with cyanobacteria on pieces of bark. The origin of the following species has been described: *Ceratophysella sigillata,*^75^ *Hypogastrura viatica,*^35^ *Hypogastrura socialis,*^38^ *Podura aquatica,*^34^ *Vertagopus sarekensis*.^36^ The following species were cultured in the laboratory: *Ceratophysella denticulata*, *Heteromurus nitidus*, *Onychiurus fimatus, Orchesella cincta*, *Protaphorura fimata*, *Sinella curviseta*, *Sminthurides aquaticus*, *Tomocerus vulgaris.*

Cultures of springtails were established and extracted at differing times after the start of the cultivation. A mixture of plaster of Paris and activated charcoal (10:1) was used as substrate. Food (baker’s yeast or cyanobacteria on pieces of bark) was given *ad libitum*. GC/MS analysis of extracts of yeast or cyanobacteria did not reveal the presence of any of the compounds discussed in the manuscript.

### Method details

#### Compound identification

##### *Anurida maritima*

The cuticular extracts of *A. maritima* (Figure S1) showed one unknown compound **ANM-A** besides cholesterol and some fatty acids (fa). The mass spectrum of **ANM-A** had a molecular ion at *m/z* 564 (Figure S23) and two prominent ions *m/z* 299 and 266, arising from the McLafferty rearrangement of an ester.^58^^,^^77^ Microderivatization with trimethylsulfonium hydroxide (TMSH) yielded the corresponding methyl ester (**S1**) with M^+^ at *m/z* 312 (Figure S24). Its mass spectrum is dominated by the ions *m/z* 101 and 88, which together with *m/z* 59 are characteristic of α-methyl-branched esters.^32^ Furthermore, the ions *m/z* 129, 143, and 171 indicate additional methyl branches at C-4 and C-6.

The alcohol part was further transformed into its trimethylsilyl derivative with *N*-methyl-*N*-(trimethylsilyl)trifluoroacetamide (MSTFA). The retention index *I* was 2024, much shorter than reported for trimethylsilylated 1-nonadecanol (2250),^78^ indicating multiple methyl branches. The mass spectrum (Figure S25) showed a base peak at *m/z* 103 of much higher intensity compared to unbranched primary silyl ethers. This indicates a methyl branch at C-2. The ions *m/z* 168 and 209 are formed by cleavage from the alkyl end next to the branch positions. The ions *m/z* 125 arise from loss of Me_3_SiOH, followed by loss of the long alkyl chain by cleavage at C-6, as indicated in Figure S25. These data suggest the alcohol to be 2,4,6-trimethylhexadecan-1-ol.

The characteristic ion *m/z* 125 is also found in the spectra of **ANM-A**. From these data, we proposed compound **5** as the structure of **ANM-A**, in which the alcohol and the acid share the same carbon skeleton.

In some extracts of this species minor amounts of homologs occurred with M^+^ at *m/z* 536 and *m/z* 522, both showing fragment *m/z* 299 and an intensive *m/z* 125, as well as *m/z* 252 or 238, respectively. Careful analysis of the mass spectra indicated chain shortening on the alcohol side with an intact 2,4,6-methyl-arrangement. The lipids were thus proposed to be 2,4,6-trimethyltridecyl and 2,4,6-trimethyltetradecyl 2,4,6-trimethylhexadecanoates. To support this hypothesis, we synthesized undecyl 2,4,6-trimethylhexadecanoate (**S13**), with an unbranched alcohol that did not show the ion *m/z* 125.

##### The lycopane compound family

The extracts of *C. denticulata*, *C. sigillata, T. bielanensis*, and *P. fimata* showed a group of similar tetraterpenes, differing in the number of double bonds. The structure of the saturated compound (**CDE-D**, **CSI-D**) was identified as lycopane (**11**) (Figure S27A) by comparison with literature data.^79^^,^^80^ As typical for methyl-branched alkanes, the secondary ions at the branching points are increased in the mass spectrum, e.g., *m/z* 253, 183, 113. The regular difference of 70 units is typical for saturated. terpenes. A second derivative (**CSI-E**) showed a molecular ion reduced by two units, which indicates a double bond. The position of the double bond was determined by the fragmentation pattern. The increased ions *m/z* 350 and 266 indicate the double bond position to be at C-14/15 (Figure S27B). To verify this, a microderivatization using dimethyl disulfide (DMDS) was performed. Interestingly, only one thiomethyl group was added at C-11 instead of two, a feature sometimes observed with trisubstituted double bonds (Figure S28). Nonetheless, the position of the thiomethyl group supports the double bond location, confirming the structure of **11**. The third derivative, containing two double bonds was identified by its mass spectrum as lycopadiene (**13**) because of the symmetric structure and fitting characteristic ions (Figure S27C). The spectrum is identical to a literature mass spectrum of **13**.^81^

##### The poduran compound family

*P. aquatica* and *X. grisea* showed a group of structurally similar terpenes. *P. aquatica* produces the tetraterpenes poduran (**18**)^34^ and decahydropentaprenylprespatane (**19**),^44^ which carry a different head group. The extracts of *X. grisea* showed four compounds with very similar mass spectra compared to **18** and **19** but with a molecular mass of 70 or 72 amu lower. The reduced mass of 70 units corresponds to one saturated isoprene unit, indicating that the side-chain is shortened. Therefore, the compounds were identified as the [7]-terpenes **22** and **23** respectively. The mass reductions by 72 amu implies one additional unsaturation. Since the ion *m/z* 69 is increased in the spectra, these compounds were tentatively identified as compounds **20** and **24**, carrying a terminal isopropylidene group.^44^ The structures of **19**, **23**, and **20** were deduced as explained below and in detail in.^44^ In short, the mass spectrum of **19** showed an intense ion at M-70, shifted from M-28 in poduran (Figures S29 and S30).^34^ Because the ion M-28 arises from cyclobutane cleavage and loss of ethylene in **18**, a loss of 70 indicates loss of a cyclopentene ring, arriving at compounds **19**, **20**, and **23**, all showing similar mass spectra.

##### *Xenylla grisea*-XGR-F

The molecular ion *m/z* 494 of **XGR-F** (Figure S31) is ten units higher than those of **22** and **23**. Still, the general overall appearance of the mass spectrum indicates that **XGR-F** is again a cyclic terpene with a different head group, maybe carrying a heteroatom. The mass spectrum is dominated by ion *m/z* 137. Noticeable is also the ion *m/z* 466 [M–28]^+^, an unusual fragmentation that can also observed in poduran derivatives **22** and **18**. Other fragments of **23** are [M–82]^+^ as well as *m/z* 161 and 189, which are all present in **XGR-F** even though they show lower intensity (Figures S30 and S31). Since all ions are present, the structure of **XGR-F** is probably similar, but the fragmentation of ion *m/z* 137 is favored. Maybe the α-position next to the polycyclic ring is saturated in **XGR-F**, and the number of rings is reduced. The higher molecular mass also indicates other changes, so more information is needed to elucidate the structure. However, the mass spectrum strongly indicated again a structure derived from a [7]-terpene.

##### *Xenylla maritima*

The mass spectrum of **XEM-B** (Figure S32) from *X. maritima* showed a molecular ion at *m/z* 552, indicating five double-bond equivalents. The fragmentation pattern indicates again a polyisoprene structure, a [8]-terpene. The high abundance of ion *m/z* 470 [M–82]^+^ may be the result of cleavage or preferred fragmentation of a cyclic head group as in **19**, **20**, or **23**, indicating a loss of a methylcyclopentene unit. Nevertheless, the difference in the spectra below *m/z* 200 indicates a different core structure compared to the head group of **19**.

The extracts of *X. maritima* showed a major unknown compound **XEM-A**. In the mass spectrum, *m/z* 490 was the highest ion, with other important ions found at *m/z* 45 and 125 (Figure S33A). The ion *m/z* 45 of medium intensity is characteristic of alkyl methyl ethers,^18^^,^^45^ known as cuticular lipids of spiders. The ion *m/z* 490 is therefore the [M-MeOH]^+^ ion indicating a pentatriacontyl methyl ether. The ion at *m/z* 125 occurs also in spectra of homomethyl branched ethers we obtained synthetically from respective available alcohols, namely 1-methoxy-2,4,6-trimethylhexadecane (**S5**) (Figure S33C) and 1-methoxy-2,4,6,8-tetramethyloctacosane (**S4**) (Figure S33B). The formation of a cyclic 1,3,5-trimethylcyclohexyl ion as a thermodynamically favorable product may explain the high abundance of ion *m/z* 125. These ions in the spectrum of **XEM-A** suggest that the compound is a methyl ether with at least three methyl branches in positions 2, 4, and 6. The tetramethyl compound **S4** shows an additional ion at *m/z* 167, 42 amu higher than *m/z* 125. This indicates the fourth methyl group. Every additional homovicinal methyl branch is marked by this 42 amu gap. In the mass spectrum of **XEM-A**, additional ions at *m/z* 209/210 and 251/252 occur, but none at *m/z* 293/294. These ions indicate additional methyl groups at positions 8, 10, and 12. This led us to the conclusion that **XEM-A** is 1-methoxy-2,4,6,8,10,12-hexamethylnonacosane (**6**).

##### *Protaphorura fimata*

Compound **PFI-C** had the mass spectrum shown in Figure S34. The spectrum was superficially similar to the spectrum of the linear tetraterpene lycopersene (**S6**), but differences are visible. Given the occurrence of various irregular terpenes in springtail surface lipids, an alternative structure would be a regular [8]-terpene connectivity, in contrast to the [4^1^ + 4^1^]-connectivity found in **S6** and its hydrogenated analogs **11**–**13**.

A more detailed view of the mass spectra of **S6** (Figure S35) and **PFI-C** (Figure S36) shows large differences. The allylic cleavages of symmetrical **S6** lead to intense ions at m/z 273, 241, 409, and 477. In contrast, unique even-numbered ions at *m/z* 274, 342, and 410 occur in the **PFI-C** spectrum (Figure S36). This spectrum resembles more that of α-geranylgeranylfarnesene (**S7**), a [7]-terpene,^61^ although the latter carries an additional double-bond (db).

While **PFI-C** has eight dbs, an additional db is formed in regular terpene hydrocarbons by elimination leading to the typical diene head groups, found e.g., in myrcene, α- and β-farnesene, or **S7**. Such a diene group seems to be missing in **PFI-C**. Nevertheless, a monoene head structure is not unprecedented in springtails, as it is present in viaticene A (**8**). The gas chromatographic retention index *I* of **PFI-C** is 3843, consistent with a linear [8]-terpene structure. We therefore tentatively assign structure **7** for **PFI-C**, called dihydrogeranylfarnesylfarnesene. Compounds **PFI-B** and **PFI-D** exhibit M^+^ ions two units higher or lower than **PFI-C** and an *I* of 3809 (**B**) and 3877 (**D**), respectively, indicating dehydro and dihydro derivatives of **7**.

##### *Anurophorus laricis*

The extracts of *A. laricis* showed a late eluting compound **ANL-B** with an estimated retention index *I* of 4020–4080. The highest visible ion in the mass spectrum was *m/z* 572. The intensive doubly unsaturated ion series *m/z* 67, 81, 95, … indicated either a C_41_-diene with M^+^ at *m/z* 572, a C_41_-enol, or a C_41_-dienal, the latter both with [M–18]^+^ at *m/z* 572 as highest ion. The *I* value could only be estimated because the available alkane standard ended at C_40_. According to the retention index, an oxidized compound seems less likely, suggesting a methyl-branched hentetracosadiene or respective cyclopropyl compound as a possible structure. The oxidized compounds would show even higher *I* values. A terpenoid backbone seems unlikely because such compounds would have smaller *I* values.

##### *Folsomia quadrioculata*

The mass spectrum of **FQU-A** showed an M^+^ at *m/z* 330 and a base ion of *m/z* 260 [M–70]^+^. Given the high molecular ion and the fragmentation pattern, **FQU-A** is most probably a polycyclic compound. The ion series *m/z* 330, 301, 287, 273, and 259 indicate the presence of a linear alkyl side chain of at least five carbons, which is atypical for terpenes. The spectrum gives the impression of a likely aromatic compound with several rings.

The mass spectrum of **FQU-D** (Figure S41) contains *m/z* 366 as the highest ion. The dominating ions are *m/z* 123 and 69. The ion *m/z* 69 is often found in spectra of linear terpenes and *m/z* 123 might indicate a cyclic head group or preferred branching. **FQU-C** also contains the ions *m/z* 69, 123, 137, 152, 165, 179, 164 and also *m/z* 256/257 but not *m/z* 366.

The mass spectrum of **FQU-E** (Figure S42) is highly similar to that of octaprenol,^82^ both with an ion at *m/z* 544 (M^+^) that is M^+^-18 in octaprenol. The ion *m/z* 544 indicates 9 dbe and *m/z* 475 [M–69]^+^ is typical for terpenes. Differences to other open-chain linear terpenes occur at the dominating ion *m/z* 109 and some minor fragments. This compound is certainly an open-chain terpene hydrocarbon. Although it remains unclear whether it is a linear [8]-terpene or a [4^1^+4^1^]-terpene, a prenylated derivative related to viaticene (**8**) seems most likely due to the nonregular occurrence of ions in the middle of the spectrum. **FQU-E** elutes close to **FQU-F** and shows a similar mass spectrum, but a less intense ion at *m/z* 109. Given this information, we postulate an unsaturated prenylated, linear tetraterpene as the structure for both **FQU-E** and **F**.

The mass spectrum of **FQU-G** (Figure S43) showed the highest ion at *m/z* 512. Prominent ions are *m/z* 55 and 81. The spectrum may fit a linear alkene with four double bonds such as hexatriacontatetraene or an aldehyde. Alternatively, an alcohol with three double bonds is possible, where *m/z* 512 is [M–18]^+^. Because of its linearity, this compound elutes later than the highly branched terpenes, underlining its biosynthetic origin from the fatty acid pathway.

##### *Folsomia candida*

The mass spectrum of **FC-A** (Figure S44) showed a molecular ion of *m/z* 446. In The extracts of *F. candida* can also be found a peak with a similar fragmentation pattern, but a molecular ion of *m/z* 474, which indicates a chain elongation of two carbons. Both mass spectra are dominated by ion *m/z* 121. The spectrum does not allow clear assignment to any of the compound classes discussed.

The mass spectra of **FC-B**–**D** (Figure S45) indicated characteristic ions for terpenes, such as the dominating ion *m/z* 69. The prominent ion *m/z* 119 is associated with a tolyl head group and a methyl branch in the α-position as in **17**.^38^ Unsaturated polyprenyl-β-curcumene derivates, [6]- and [7]-terpenes,^47^ have highly similar spectra and proportionality of the ions *m/z* 69 and 119 compared to those of **FC-B**–**D**. Compound **FC-D** had an M^+^ ion at *m/z* 548, translated into an [8]-terpene with seven dbe, from which four are in the aromatic head group. The polyprenyl chain must therefore be partly hydrogenated, but the exact positions remain unknown. Nevertheless, the base peak at m/z 69 indicated an unsaturated isoprene unit at the tail. The molecular ions of **FC-B** and C cannot be identified due to their low abundance, but the difference in retention indices (*I*_C_ 3341, *I*_D_ 3371, *I*_E_ 3796; Δ*I* 425–455) indicates that the polyprenyl chain is one isoprene unit shorter, resulting in [7]-terpenes. Previously an Δ*I* of 470 was reported for one isoprene unit in open-chain terpenes.^61^ The slight differences might be due to differences in saturation.

##### *Cryptopygus clavatus*

The mass spectrum of **CCL-C** is shown in Figure S46.

##### *Heteromurus nitidus*

The compound identification of nitidane (**9**) (Figure S47) has recently been described.^46^ The mass spectrum of **HEN-B** (Figure S48) shows clear characteristics of a highly-branched terpene, like the dominating ion *m/z* 69. The M^+^ ion is found at *m/z* 544, as expected for a [8]-terpene with nine dbe.

The mass spectrum of **HEN-C** (Figure S49) is similar. The highest measured ion is *m/z* 529, which most probably is not the molecular ion but might be the result of a primary fragmentation. The expected M^+^ for a [9]-terpene would be *m/z* 612. Whether the branching in **HEN-B** and **HEN-C** is similar to nitidane (**9**) remains unclear.

##### *Orchesella cincta*

The two compounds **OSA-A** and **B** showed similar mass spectra indicating long-chain alkenes (Figure S50). Dimethyl disulfide (DMDS) addition (Figure S51) and microozonolysis showed that both **OSA-A** and **OSA-B** were terminal alkenes. Microhydrogenation led to the saturated alkanes (**S10**, **S11**), their mass spectra together with the retention indices (*I* 3418 and 3577) allowed the allocation of several methyl branches (Figure S52). However, the double bond could be located at both ends of the molecules so far, leading to two possible structures. GC-Orbitrap-MS solved this problem. The mass spectra of the aldehydes obtained by ozonolysis showed only two or three unique O-containing ions, e.g., *m/z* 141.1274 (C_9_H_17_O) and 434.4483 (C_30_H_58_O), formed by cleavage next to the methyl branching, thus indicating the original position of the double bond relative to the methyl groups. Together, these data confirmed 9,29-dimethylpentatriacont-1-ene (**19**) to be **OSA-A**. Similarly, **OSA-B** was identified as 3,11,23-trimethyltritriacont-1-ene (**20**).

##### *Sinella curviseta*

The molecular formula of **SIC-A** is C_36_H_62_ with six dbe, as determined by GC/HR-MS. Microhydrogenation showed an M^+^ ion at *m/z* 506 (Figure S53), indicating six double bonds in the molecule. Analysis of the mass spectra of the saturated compound indicated several methyl branches. Given the high number of double-bonds and methyl groups, a terpenoid structure was possible. Still, the retention index of the saturated compound (*I* 3315) is higher than the expected value of a saturated [7]-terpene with an additional methyl group. We therefore assign **SIC-A** to be a highly unsaturated linear hydrocarbon. While the location of the double bonds cannot be deduced using mass spectrometric methods, the mass spectrum of the hydrogenated compound hinted at possible structures such as **S12** or **S13** (Figure S54).

##### *Tomocerus vulgaris*

The mass spectrum of **TOM-E** (Figure S55) shows *m/z* 505 [M-15]^+^ as the heaviest ion. The fragmentation pattern is typical for long-chain methyl-branched alkanes. The increased ion pairs at *m/z* 140/407 and 267/280 indicate methyl-branches at C-9 and C-17, which gives 9,17-dimethylpentatriacontane (**4**) as the structure. The retention index *I* 3562 is typical for internal branched dimethylalkanes.^55^

#### Syntheses

##### Synthesis of (2*S*,4*S*,6*S*)-2,4,6-trimethylhexadecyl (2*S*,4*S*,6*S*)-2,4,6-trimethylhexadecanoate (5)

**Scheme 1:**
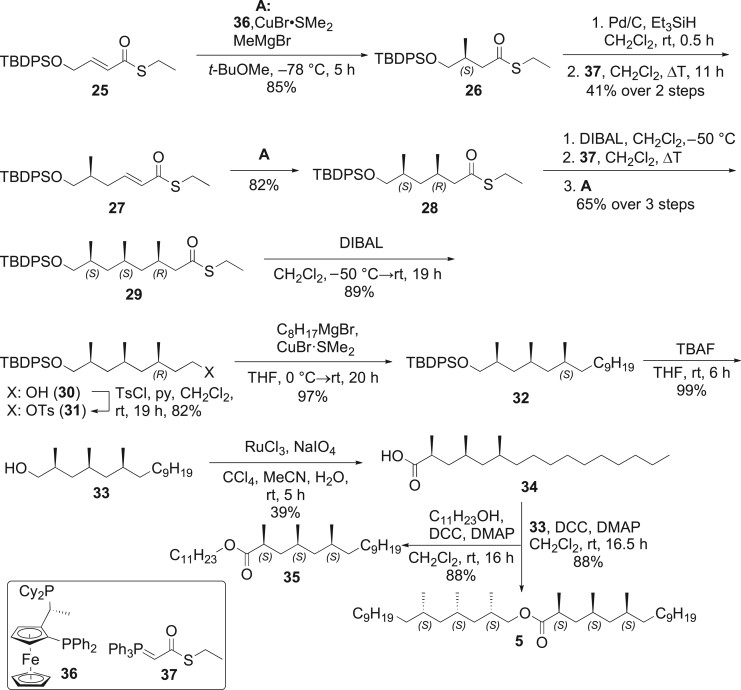
Synthesis of tetradecyl (2*S*,4*S*,6*S*)-2,4,6-trimethylhexadecyl (2*S*,4*S*,6*S*)-2,4,6-trimethylhexadecanoate (**5**) Please note that the configurational prefixes change during the synthesis due to the CIP rules.

To confirm our proposal, ester **5** was synthesized according to Scheme 1. The synthesis is based on a general procedure developed by Feringa et al. for the stereoselective synthesis of homo-vicinal oligo-methyl-branched acids in an enantioselective manner.^41^^,^^42^^,^^43^ The synthesis of 2,4,6- trimethylhexadecanoic acid (**34**) started with thioester **25**^32^ that underwent an enantioselective 1,4-addition with methylmagnesium bromide using Josiphos (**36**) as a ligand for CuBr. The resulting thioester **26** was then reduced by Pd/C with Et_3_SiH as a hydrogen source, giving the corresponding aldehyde that underwent a Wittig reaction with **37**^32^ forming the α,β-unsaturated thioester **27**. Thioester **27** was used as a substrate for the second enantioselective 1,4-addition with methylmagnesium bromide to form compound **28**. The reaction steps were then repeated to obtain thioester **29** after reduction with diisobutylaluminium hydride (DIBAL) delivered the corresponding alcohol **30**, followed by tosylation. Tosylate **31** then underwent substitution with C_11_H_23_MgBr to give compound **32**. The cleavage of the silyl ether with tetra-*n*-butylammonium fluoride (TBAF) delivered alcohol **33** that was oxidized with RuO_4_ to finally furnish carboxylic acid **34**. Esterification with **33** or undecanol yielded (2*S*,4*S*,6*S*)-2,4,6-trimethylhexadecyl (2*S*,4*S*,6*S*)-2,4,6-trimethylhexadecanoate (**5**) and undecyl (2*S*,4*S*,6*S*)-2,4,6-trimethylhexadecanoate (**35**) as final products. Product **5** showed identical mass spectral and gas chromatographic behavior with the natural compound **ANM-A**, proving its structure.

##### Stereochemistry of compound ANM-A (5)

To elucidate the stereochemistry of **ANM-A**, an independently synthesized sample of **34** comprising the (2*R*∗,4*R*∗,6*R*∗)- and (2*R*∗,4*R*∗,6*S*∗)-diastereomers as major components and the others as traces was separated on a Hydrodex β-6TBDM GC phase (Figure S26). The separation required very long elution times. The enantiomer pairs are usually separated close to each other. The large peak pair at 258 min are the (2*R*^∗^,4*R*^∗^,6*R*^∗^)-esters, as indicated by injection of methyl (2*S*,4*S*,6*S*)-**34**. The other diastereomers are well separated. Coinjection with the methyl ester derived from A**NM-A**, obtained by transesterification with TMSH, showed that both compounds eluate simultaneously. We assume that the alcohol part is a biosynthetic reduction product of the precursor acid **33**. Therefore, we propose compound **ANM-A** to be (2*S*,4*S*,6*S*)-2,4,6-trimethylhexadecyl (2*S*,4*S*,6*S*)-2,4,6-trimethylhexadecanoate (**5**).

##### Syntheses for poduran type compound identification

**Scheme 2:**
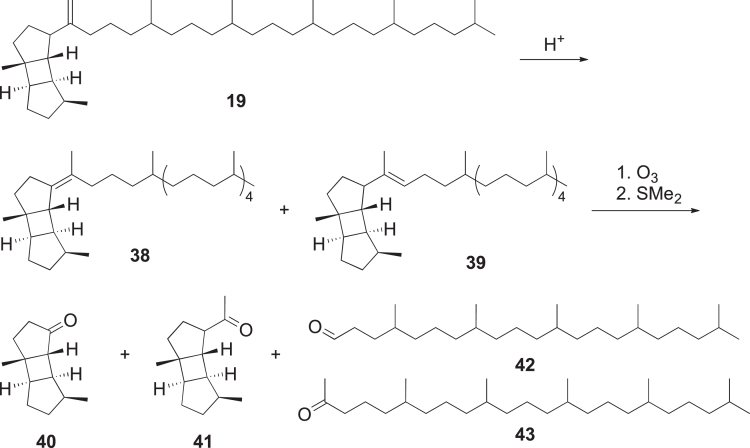
Derivatization of natural terpenoid **19**

**Scheme 3:**
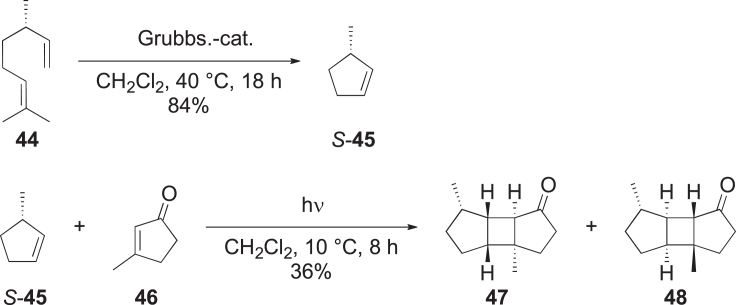
Synthesis of the tricycles **47** and **48**

To verify the proposals, an acidic isomerization of the double bond of **19** and ozonolysis were performed, yielding two tricyclic ketones (**40**, **41**) as cleavage products, with the intact head group (Scheme 2). To prove the structure, ketone **40** was synthesized by photochemical reaction from 3-methyl-2-cyclopentenone (**45**) and 3-methylcyclopentene (**46**) (Scheme 3). The synthetic product and the cleavage ketone of **19** were identical, thus proving the constitution of the tricycle. Further synthetic and chromatographic work established the absolute configuration around the head group of **19** as shown in Figure S29.^44^

#### Genetic analysis

We performed the NCBI database search to investigate whether Springtails harbor genes for the *de novo* synthesis of cholesterol. We used BLASTP to search for homologs of genes of the cholesterol biosynthetic pathway (Figure S56)^65^ in the gene sets of Springtails (taxid: 30001).

#### General experimental procedures

All reactions were performed in oven-dried glassware under a nitrogen atmosphere. Solvents were dried according to standard procedures. Column chromatography: silica 60 (0.063–0.200 mm, 70–230 mesh ASTM). Thin-layer chromatography (TLC): Polygram SIL G/UV silica 60, 0.20 mm. Compounds were stained with potassium permanganate solution. IR spectra were measured on a Bruker Tensor 27 (diamond-ATR) or Agilent Technologies 7890B gas chromatograph equipped with an HP5 phase connected to a Dani Instruments DiscovIR DDFTIRInterface. NMR spectra were recorded either on Avance III HD 300N (^1^H NMR: 300 MHz, ^13^C NMR: 76 MHz), AVII 400 (^1^H NMR: 400 MHz, ^13^C NMR: 101 MHz), or AVIIIHD-500 MHz (^1^H NMR: 500 MHz, ^13^C NMR: 125 MHz) instruments. Mass spectra were recorded with a combination of an Agilent Technologies 5977B gas chromatograph connected to an Agilent Technologies 8860 Series MSD. Gas chromatographic retention indices were calculated against a series of *n*-alkanes according to van den Dool and Kratz^83^ using a standard HP-5 phase. An Exactive GC orbitrap mass spectrometer (ThermoScientific, Bremen, Germany) was used for high-resolution MS. The resolution was set to 60,000 (FWHM; instrument setting at 200 u). Mass range was 50–650 u and 2 micro scans were averaged per data scan. Automated gain control (AGC target) was set to 1 × 10^6^ and maximum inject time was set to “auto.” Auxiliary temperatures were set to 290°C for both transfer lines 1 and 2 and the temperature of the electron ionization source was set to 220°C. EI was performed at 70 eV energy in positive mode. Helium (carrier gas) and nitrogen (supply for the C-Trap) were equipped with gas purification cartridges to trap moisture and organic impurities of the gases (Thermo Scientific, Bremen, Germany). Column bleed ion at 207.03235 u was used as lock mass for internal mass calibration of the data. For chemical ionization in positive mode (CIP), methane (99.995%) was used as CI-gas at a 1.5 mL/min flow rate. GC oven programs are reported in the SI. Compound identification was performed by different methods including GC/IR analysis, chemical microderivatization, gas chromatographic retention indices, and mass spectrometry using internal databases, data interpretation as well as NIST17^82^ and MACE^76^ databases.

#### Gas chromatographic analysis

GC/MS analyses were performed using an HP-5 phase (30.0 m × 0.25 mm) in combination with temperature programs A and B or a BPX-5 phase (25.0 m × 0.25 mm) in combination with temperature programs C and D.

Temp. A: initial temp. 50°C for 5 min then 5°C min^−1^ to 320°C holding time for at least 5 min. Temp. B: initial temp. 100°C for 5 min then 10°C min^−1^ to 320°C holding time for at least 5 min. Temp. C: initial temp. 45°C for 5 min then 5°C min^−1^ to 300°C holding time for at least 5 min. Temp. D: initial temp. 45°C for 5 min then 10°C min^−1^ to 300°C holding time for at least 5 min.

#### Microderivatization

##### Hydrogenation

Microhydrogenation was performed according to a protocol published by us before.^36^ A minute amount of Pd/C was added to a solution of the analyte or an extract (50–100 μL). A hydrogen atmosphere was applied for 3 h or until GC/MS analysis showed complete conversion. The catalyst was then removed by filtration through a Celite pad. The resulting solution was analyzed by GC/MS.

##### Dimethyl disulfide additions

Double bond elucidation was performed according to a protocol of Francis and Veland.^84^ *Dimethyl disulfide* (DMDS, freshly distilled, 50 μL) and a solution of iodine in CH_2_Cl_2_ (0.25 M, 5 μL) were added to a solution of the analyte (50–100 μL). The resulting solution was heated overnight to 60°C–80°C. The reaction was quenched by the addition of aq. Na_2_S_2_O_3_ solution (50 μL). The phases were carefully separated in a pipette and the aqueous phase was extracted with pentane (3 × 50 μL). The resulting solution was analyzed by GC/MS.

##### Transesterification with trimethylsulfonium hydroxide (TMSH)

Transesterification of fatty acids was performed according to a protocol of Müller et al.^85^ TMSH (0.2 M in MeOH, 50 μL) was added to a solution of the analyte (50–100 μL). The resulting solution was either analyzed directly by GC/MS or heated 1 h to 60°C–80°C first.

##### Silylation with *N*-methyl-*N*-(trimethylsilyl)trifluoracetamide (MSTFA)

The silylation of alcohols was performed after a protocol of Pasikanti et al.^86^ MSTFA (50 μL) was added to a solution of the analyte (50–100 μL). The resulting solution was heated 1 h to 60°C–80°C and the volatiles were removed by a gentle nitrogen flow. The residue was taken up in 20 μL CH_2_Cl_2_ and analyzed by GC/MS.

##### Ozonolysis

Microozonolysis was performed according to a protocol of Bello et al.^35^ Ozone was bubled through CH_2_Cl_2_ (1 mL) in a 1.5 mL vial cooled to −78°C. A portion of the saturated solution (200 μL) was added to a solution of the analyte or extract (50–100 μL). After 15 min, the reaction was quenched by the addition of dimethylsulfide in CH_2_Cl_2_ (5% v/v), and the volatiles were removed by a gentle nitrogen flow, reducing the volume to about 30 μL. This solution was used for GC/MS analysis.

#### Synthetic protocols

The synthesis of starting materials, compounds **25–28** was performed as previously described.^32^

##### Preparation of *S*-ethyl (3*R*,5*S*,7*S*)-8-((tert-butyldiphenylsilyl)oxy)-3,5,7-trimethyloctanethioate (29)

*S*-ethyl (3*S*,5*R*)-6-((*tert*-butyldiphenylsilyl)oxy)-3,5-dimethylhexanethioate (**28**, 3418 mg, 7.72 mmol, 1.0 eq.) was dissolved in CH_2_Cl_2_ (125 mL). The mixture was cooled to −50°C. Diisobutylaluminium hydride (1 M in cyclohexane, 10.04 mmol, 10.04 mL) was added slowly at −50°C. After 3 h Rochelle solution (100 mL) was added to quench the reaction. The mixture was allowed to warm up to room temperature. After 30 min stirring at room temperature the phases were separated. The aqueous phase was extracted with CH_2_Cl_2_ (100 mL). After drying the combined organic phases over MgSO_4_, the solvent was removed under reduced pressure. The residue was purified by column chromatography (pentane/Et_2_O; 20:1). The aldehyde was obtained as a colorless oil (2801 mg, 7.321 mmol, 95%) and was used directly in the Wittig olefination.

*S*-Ethyl (*S*)-2-(triphenyl-λ5-phosphaneylidene)ethanethioate (**37**, 4471 mg, 12.26 mmol, 1.7 eq.) was added to a stirred solution of the aldehyde (2801 mg, 7.321 mmol, 95%) in CH_2_Cl_2_ (40 mL). The mixture was heated to reflux for 35 h. The solvent was removed under reduced pressure and the residue was purified by flash chromatography (pentane/Et_2_O; 40:1). *S*-Ethyl (5*S*,7*S*,*E*)-8-((*tert*-butyldiphenylsilyl)oxy)-5,7-dimethyloct-2-enethioate was obtained as a colorless oil (3073 mg, 6.555 mmol, 90%). ${\left[\alpha \right]}_{D}^{25}$ = −9.5 (10 mg/mL; CHCl_3_). FT-IR: *ν*/cm^−1^ = 2958, 2929, 2858, 1670, 1632, 1465, 1428, 1385, 1309, 1264, 1189, 1108, 1084, 1000, 973, 939, 891, 823, 801, 740, 703, 615, 565, 537. ^1^H-NMR: (300 MHz, CDCl_3_) *δ*/ppm = 7.71–7.62 (m, 1H), 7.46–7.33 (m, 1H), 6.83 (ddd, *J* = 15.2, 7.9, 7.1 Hz, 1H), 6.07 (dt, *J* = 15.4, 1.4 Hz, 1H), 3.46 (ddd, *J* = 16.1, 9.8, 5.8 Hz, 1H), 2.93 (q, *J* = 7.4 Hz, 1H), 2.24–2.11 (m, 1H), 1.91 (m, 1H), 1.79–1.59 (m, 1H), 1.39 (dt, *J* = 13.7, 6.8 Hz, 1H), 1.27 (t, *J* = 7.4 Hz, 1H), 1.11–1.02 (m, 2H), 1.02–0.88 (m, 1H), 0.84 (d, *J* = 6.6 Hz, 1H). ^13^C-NMR, DEPT: (76 MHz, CDCl_3_) *δ*/ppm = 190.1 (C_q_), 144.19 (CH), 135.8 (CH_Ar_), 134.1 (C_q_), 130.0 (CH_Ar_), 129.7 (CH_Ar_), 127.7 (CH), 68.8 (CH_2_), 40.9 (CH_2_), 39.6 (CH_2_), 33.3 (CH), 30.1 (CH), 27.0 (CH_3_), 23.2 (CH_2_), 20.3 (CH_3_), 19.4 (CH), 17.8 (CH_3_), 15.0 (CH_3_). EI-MS (70 eV): *m/z* (%) = 411 (72), 243 (35), 199 (100), 183 (46), 181 (29), 175 (26), 135 (40), 95 (34), 55 (48), 41 (30). For NMR spectra see Figure S57.

The synthesis of **29** was performed according to^43^ using (*R*)-1-[(*S*_P_)-2-(diphenylphosphino)ferrocenyl]ethyldicyclohexylphosphine (Josiphos, **36**, 0.08 mmol, 50 mg, 0.012 eq.), CuBr·SMe_2_ (0.06 mmol, 13 mg, 0.01 eq.), methyl *tert*-butyl ether (MTBE, 50 mL), MeMgBr (7.53 mmol, solution in diethyl ether), and *S*-ethyl (5*S*,7*S*,*E*)-8-((*tert*-butyldiphenylsilyl)oxy)-5,7-dimethyloct-2-enethioate (6.27 mmol, 2940 mg, 1 eq.) to afford **S7** as a colorless oil (2311 mg, 4.767 mmol, 76%). ${\left[\alpha \right]}_{D}^{25}$ = −7.8 (10 mg/mL; CHCl_3_). FT-IR: *ν*/cm^−1^ = 2957, 2929, 2859, 1689, 1459, 1427, 1382, 1263, 1189, 1002, 972, 943, 823, 792, 740, 703, 615. ^1^H–NMR: (300 MHz, CDCl_3_) *δ*/ppm = 7.69–7.63 (m, 4H), 7.45–7.33 (m, 6H), 3.45 (ddd, *J* = 16.2, 9.8, 5.8 Hz, 2H), 2.86 (q, *J* = 7.4 Hz, 2H), 2.36 (ddd, *J* = 22.9, 14.1, 6.8 Hz, 1H), 2.16–2.01 (m, 1H), 1.71 (m, 1H), 1.56–1.41 (m, 1H), 1.41–1.15 (m, 4H), 1.05 (s, *J* = 2.9 Hz, 9H), 1.00–0.85 (m, 9H), 0.82 (d, *J* = 6.5 Hz, 3H). ^13^C–NMR, DEPT: (76 MHz, CDCl_3_) *δ*/ppm = 199.5 (C_q_), 135.8 (CH_Ar_), 134.2 (C_Ar_), 129.6 (CH_Ar_), 127.7 (CH_Ar_), 68.9 (CH_2_), 51.1 (CH_2_), 44.9 (CH_2_), 41.3 (CH_2_), 34.3 (CH), 33.3 (CH), 28.8 (CH), 27.8 (CH), 27.0 (CH_3_), 23.4 (CH_2_), 22.5 (CH_2_), 20.7 (CH_3_), 20.6 (CH_3_), 19.5 (CH), 18.1 (CH_3_), 15.0 (CH_3_), 14.2 (CH_3_). EI-MS (70 eV): *m/z* (%) = 427 (84), 428 (28), 199 (100), 183 (32), 181 (22), 135 (27), 123 (24), 69 (23), 57 (21), 43 (28). HR-CIP-MS: *m/z* = 483.27652 [M–H]^+^. *theo*.: 483.27726.

##### Preparation of (3*R*,5*S*,7*S*)-8-((tert-butyldiphenylsilyl)oxy)-3,5,7-trimethyloctan-1-ol (30)

Compound *S*-ethyl (3*R*,5*S*,7*S*)-8-((*tert*-butyldiphenylsilyl)oxy)-3,5,7-trimethyl-octanethioate (**29**, 2250 mg, 4.64 mmol, 1.0 eq.) was dissolved in CH_2_Cl_2_ (20 mL). DIBAL (1 M in cyclohexane, 6.03 mmol, 6.033 mL) was added slowly at −50°C. The mixture was stirred 15 h at −50°C. Rochelle solution (10 mL) was added and the mixture was allowed to warm up to room temperature. After stirring 30 min at room temperature the phases were separated. The aqueous phase was extracted with CH_2_Cl_2_ (3 × 30 mL). The combined organic phases were dried over Na_2_SO_4_ and the solvent was removed under reduced pressure. The previous steps were repeated with the crude aldehyde. The crude alcohol was purified by flash chromatography (pentane/Et_2_O; 1:1) to yield the product (**30**) as a colorless oil (1757 mg, 4.122 mmol, 89%). ${\left[\alpha \right]}_{D}^{25}$ = −6.3 (10 mg/mL; CHCl_3_). FT-IR: *ν*/cm^−1^ = 3321, 3071, 2955, 2928, 2858, 1461, 1428, 1379, 1189, 1109, 1006, 972, 939, 823, 793, 702, 614, 567, 547. ^1^H–NMR: (300 MHz, CDCl_3_) *δ*/ppm = 7.71–7.63 (m, 4H), 7.46–7.33 (m, 6H), 3.74–3.56 (m, 2H), 3.46 (ddd, *J* = 16.2, 9.8, 5.8 Hz, 2H), 1.82–1.44 (m, 4H), 1.44–1.12 (m, 4H), 1.05 (s, 9H), 0.98–0.78 (m, 11H). ^13^C-NMR, DEPT: (76 MHz, CDCl_3_) *δ*/ppm = 135.8 (C_Ar_), 134.3 (C_q_), 129.6 (C_Ar_), 127.7 (C_Ar_), 68.9 (CH_2_), 61.4 (CH_2_), 45.6 (CH_2_), 41.5 (CH_2_), 39.7 (CH_2_), 33.3 (CH), 27.72 (CH), 27.0 (CH_3_), 20.9 (CH_2_), 20.6 (CH_2_), 19.5 (CH_2_), 18.2 (CH_2_). EI-MS (70 eV): *m/z* (%) = 200 (20), 199 (100), 183 (16), 181 (22), 97 (88), 83 (54), 57 (24), 55 (41), (39), 41 (17). For NMR spectra see Figure S58.

##### Preparation of (3*R*,5*S*,7*S*)-8-((tert-butyldiphenylsilyl)oxy)-3,5,7-trimethyloctyl 4-methylbenzenesulfonate (31)

Compound (3*R*,5*S*,7*S*)-8-((*tert*-butyldiphenylsilyl)oxy)-3,5,7-trimethyloctan-1-ol (**30**) and pyridine (7.80 mmol, 617 mg, 2.0 eq.) were dissolved in CH_2_Cl_2_ (25 mL). Tosyl chloride (1487 mg, 7.80 mmol, 2 eq.) was added. The mixture was stirred for 19 h at room temperature. The reaction was quenched through the addition of H_2_O (50 mL). The phases were separated and the aqueous phase was extracted with CH_2_Cl_2_ (3 × 50 mL). The combined organic phases were dried over MgSO_4_ and the solvent was removed under reduced pressure. The residue was purified through flash chromatography (pentane/Et_2_O; 20:1→4:1) to obtain **31** as a colorless oil (1862 mg,3.205 mmol, 82%). ${\left[\alpha \right]}_{D}^{25}$ = −6.3 (10 mg/mL; CHCl_3_). FT-IR: *ν*/cm^−1^ = 3066, 2957, 2925, 2861, 1596, 1464, 1429, 1363, 1299, 1257, 1213, 1180, 1105, 947, 890, 818, 744, 702, 664, 615, 557. ^1^H-NMR: (300 MHz, CDCl_3_) *δ*/ppm = 7.78 (dt, *J* = 8.3, 2.0 Hz, 2H), 7.66 (ddd, *J* = 6.5, 3.9, 2.1 Hz, 4H), 7.45–7.29 (m, 8H), 4.12–3.97 (m, 2H), 3.43 (ddd, *J* = 16.3, 9.8, 5.8 Hz, 2H), 2.43 (s, 1H), 1.77–1.50 (m, 2H), 1.50–1.36 (m, 1H), 1.36–1.20 (m, 1H), 1.15–1.06 (m, 1H), 1.06–1.03 (m, 4H), 0.90 (d, *J* = 6.7 Hz, 2H), 0.87–0.79 (m, 1H), 0.76 (dd, *J* = 6.5, 1.2 Hz, 3H). ^13^C-NMR, DEPT: (76 MHz, CDCl_3_) *δ*/ppm = 144.7 (C_q_), 135.8 (C_Ar_), 134.2 (C_q_), 133.4 (C_q_), 129.9 (C_Ar_), 129.7 (C_Ar_), 128.0 (C_Ar_), 127.7 (C_Ar_), 69.2 (CH_2_), 68.9 (CH_2_), 45.1 (CH_2_), 41.3 (CH_2_), 35.4 (CH_2_), 33.2 (CH), 27.6 (CH), 27.0 (CH_3_), 26.8 (CH_2_), 21.8 (CH_3_), 20.8 (CH_3_), 20.1 (CH_3_), 19.44 (C_q_), 18.1 (CH_3_). EI-MS (70 eV): *m/z* (%) = 309 (22), 199 (94), 183 (27), 181 (27), 97 (100), 83 (63), 69 (36), 57 (50), 55 (37), 41 (22). For NMR spectra see Figure S59.

##### Preparation of tert-butyldiphenyl(((2*S*,4*S*,6*S*)-2,4,6-trimethylhexadecyl)oxy)silane (32)

1-Bromoctane (2500 mg, 12.95 mmol) was added to magnesium turnings (315 mg, 12.95 mmol) covered with THF (45 mL). After heating to reflux for 1 h 15 mL of the solution (4.32 mmol, 1.6 eq.) was transferred to a stirred solution of (3*R*,5*S*,7*S*)-8-((*tert*-butyldiphenylsilyl)oxy)-3,5,7-trimethyloctyl 4-methylbenzenesulfonate (**31**) (1600 mg, 275 mmol, 1.0 eq.) and CuBr·SMe_2_ (113 mg, 0.55 mmol, 0.2 eq.) at 0°C. The mixture was stirred 1 h at 0°C. Afterward, the mixture was allowed to warm up to room temperature and stirred further for 20 h at room temperature. Saturated NH_4_Cl solution (40 mL) and diethyl ether (50 mL) were added. The phases were separated and the aqueous phase was extracted with diethyl ether (3x 60 mL). The combined organic phases were dried over MgSO_4_ and the solvent was removed under reduced pressure. The residue was purified by flash chromatography (pentane). The silane **32** was obtained as a colorless oil (1399 mg, 2.68 mmol, 97%). ${\left[\alpha \right]}_{D}^{25}$ = −6.0 (10 mg/mL; CHCl_3_). FT-IR: *ν*/cm^−1^ = 3071, 2955, 2923, 2854, 1590, 1464, 1428, 1378, 1239, 1188, 1108, 1004, 972, 940, 822, 738, 701, 614, 568, 549. ^1^H-NMR: (300 MHz, CDCl_3_) *δ*/ppm = 7.71–7.64 (m, 4H), 7.45–7.33 (m, 6H), 3.46 (ddd, *J* = 16.2, 9.8, 5.8 Hz, 2H), 1.72 (octett, *J* = 6.7 Hz, 1H), 1.60–1.11 (m, 22H), 1.05 (s, *J* = 2.9 Hz, 9H), 1.02–0.74 (m, 16H). ^13^C-NMR, DEPT: (76 MHz, CDCl_3_) *δ*/ppm = 135.8 (CH_Ar_), 134.3 (C_Ar_), 129.6 (CH_Ar_), 127.7 (CH_Ar_), 69.0 (CH_2_), 45.6 (CH_2_), 41.6 (CH_2_), 36.7 (CH_2_), 33.4 (CH), 32.1 (CH_2_), 30.2 (CH_2_), 30.2 (CH), 29.9 (CH_2_), 29.9 (CH_2_), 29.8 (CH_2_), 29.5 (CH_2_), 27.8 (CH), 27.1 (CH), 22.9 (CH_2_), 21.1 (CH_3_), 20.7 (CH_3_), 19.5 (C_q_), 18.2 (CH_3_), 14.3 (CH_3_). EI-MS (70 eV): *m/z* (%) = 466 (17), 465 ([M-^t^Bu]^+^, 44), 429 (<1), 200 (19), 199 (100), 183 (20), 97 (22), 83 (24), 57 (34), 43 (23), 111 (18). HR-CIP-MS: *m/z* = 521.41730 [M–H]^+^. *theo*.: 521.41732. For NMR spectra see Figure S60.

##### Preparation of (2*S*,4*S*,6*S*)-2,4,6-trimethylhexadecan-1-ol (33)

Tetra-*n*-butylammonium fluoride (1M in THF, 7.389 mL,7.39 mmol) was added to a stirred solution of *tert*-butyldiphenyl(((2*S*,4*S*,6*S*)-2,4,6-trimethylhexadecyl)oxy)silane (**32**) (1288 mg, 2.46 mmol, 1.0 eq.) in THF (20 mL). The mixture was stirred for 5.5 h at room temperature. The solvent was removed under reduced pressure. The residue was purified by flash chromatography (pentane→pentane/Et_2_O; 10:1). **33** was obtained as a mixture with the free siloxane as a colorless oil (1268 mg, 2.42 mmol, 99%). ${\left[\alpha \right]}_{D}^{25}$ = −8.4 (10 mg/mL; CHCl_3_). FT-IR: *ν*/cm^−1^ = 3361, 3072, 3051, 2955, 2923, 2854, 1463, 1428, 1377, 1261, 1190, 1112, 1030, 939, 857, 820, 739, 700, 607, 569, 555, 532. ^1^H-NMR: (300 MHz, CDCl_3_) *δ*/ppm = 7.74–7.69 (m, 1H, siloxane), 7.44–7.33 (m, 1H, siloxane), 3.45 (ddd, *J* = 17.3, 10.5, 5.9 Hz, 2H), 1.81–1.64 (m, 1H), 1.64–1.38 (m, 4H), 1.39–1.11 (m, 20H), 1.11–1.05 (m, 1H, siloxane), 1.04–0.59 (m, 16H). ^13^C-NMR, DEPT: (76 MHz, CDCl_3_) *δ*/ppm = 135.5 (C_Ar_, siloxane), 135.0 (CH_Ar,_ siloxane), 129.7 (CH_Ar,_ siloxane), 127.8 (CH_Ar,_ siloxane), 68.4 (CH_2_), 45.3 (CH_2_), 41.4 (CH_2_), 36.6 (CH_2_), 33.2 (CH), 32.07 (CH), 30.2 (CH_2_), 30.1 (CH), 29.9 (CH_2_), 29.9 (CH_2_), 29.8 (CH_2_), 29.5 (CH_2_), 27.7 (CH_3_, siloxane), 27.0 (CH_2_), 26.7 (CH_3_), 22.8 (CH_2_), 21.1 (CH_3_), 20.6 (CH_3_), 17.7 (CH_3_), 14.3 (CH_3_). EI-MS (70 eV): *m/z* (%) = 266 (2), 252 (1), 237 (3), 209 (19), 168 (39), 125 (49), 85 (35), 83 (100), 71 (49), 69 (83), 57 (96), 55 (93), 43 (86), 41 (63). ***I* =** 1983 (HP-5 phase). HR-CIP-MS: *m/z* = 283.29962 [M–H]^+^. *theo*.: 283.29954. For NMR spectra see Figure S61.

##### Preparation of (2*S*,4*S*,6*S*)-2,4,6-trimethylhexadecanoic acid (34)

To a solution of (2*S*,4*S*,6*S*)-2,4,6-trimethylhexadecan-1-ol (**33**) (300 mg, 1.5 mmol, 1.0 eq.) in a mixture of MeCN (12 mL), H_2_O (24 mL), and CCl_4_ (12 mL) was added RuCl_3_ (55 mg, 0.26 mmol, 0.3 eq.) and NaIO_4_ (1128 mg, 5.27 mmol, 5.0 eq.). The mixture was stirred for 5 h at room temperature. Afterward, it was diluted with CH_2_Cl_2_ (40 mL) and H_2_O (10 mL) was added. The phases were separated and the aqueous phase was extracted with CH_2_Cl_2_ (3x 40 mL). The combined organic phases were dried over MgSO_4_ and the solvent was removed under reduced pressure. The residue was purified by flash chromatography (pentane/Et_2_O/AcOH; 90:10:1) to obtain **34** as a colorless oil (123 mg, 0.41 mmol, 39%). ${\left[\alpha \right]}_{D}^{25}$ = −7.0 (10 mg/mL; CHCl_3_). FT-IR: *ν*/cm^−1^ = 2956, 2922, 2853, 2661, 1706, 1462, 1416, 1378, 1234, 1154, 1092, 942, 814, 722, 641, 536. ^1^H-NMR: (300 MHz, CDCl_3_) *δ*/ppm = 2.66–2.50 (m, 1H), 1.75 (ddd, *J* = 13.9, 9.4, 4.8 Hz, 1H), 1.65–1.41 (m, 2H), 1.41–1.22 (m, 18H), 1.22–0.92 (m, 9H), 0.92–0.79 (m, 11H). ^13^C-NMR, DEPT: (76 MHz, CDCl_3_) *δ*/ppm = 183.8 (C_q_), 45.4 (CH_2_), 41.2 (CH_2_), 37.5 (CH), 37.1 (CH_2_), 34.3 (CH_2_), 32.1 (CH_2_), 30.2 (CH_2_), 30.0 (CH), 29.9 (CH_2_), 29.9 (CH_2_), 29.8 (CH_2_), 29.5 (CH_2_), 28.3 (CH), 27.1 (CH_2_), 22.9 (CH_2_), 22.5 (CH_2_), 20.5 (CH_3_), 20.2 (CH_3_), 18.1 (CH_3_), 14.3 (CH_3_), 14.2 (CH_3_). A small portion was converted into the respective methyl ester by treatment with trimethylsilyldiazomethane. EI-MS (70 eV): *m/z* (%) = 312 ([M]^+^, 3), 297 ([M-CH_3_]^+^, <1), 102 (14), 101 (100), 89 (11), 88 (90), 71 (10), 69 (19), 57 (18), 55 (15), 43 (16), 41 (12). *I* = 1995 (HP-5 phase). HR-EI-MS: *m/z* = 298.28677 [M–H]^+^. *theo*.: 298.28663. For NMR spectra see Figure S62.

##### Preparation of undecyl (2*S*,4*S*,6*S*)-2,4,6-trimethylhexadecanoate (35)

To a solution of (2*S*,4*S*,6*S*)-2,4,6-trimethylhexadecanoic acid (**34**) (15 mg, 0.050 mmol, 1.0 eq.), undecanol (10 μL, 0.050 mmol, 10.0 eq.) and 4-dimethylaminopyridine (1 mg, 0.005 mmol, 0.1 eq.) in CH_2_Cl_2_ (0.8 mL) was added *N*,*N*′-dicyclohexylcarbodiimide (10 mg, 0.050 mmol, 1.0 eq.). The mixture was stirred for 16 h at room temperature. The solvent was removed and the residue was purified by flash chromatography (pentane) to obtain **34** as a colorless oil (20 mg, 0.044 mmol, 88%). ${\left[\alpha \right]}_{D}^{25}$ = +10.6 (9 mg/mL; CHCl_3_). FT-IR: *ν*/cm^−1^ = 2957, 2926, 2854, 1707, 1467, 1420, 1380, 1301, 1240, 1110, 958, 822, 723. ^1^H–NMR: (600 MHz, CDCl_3_) *δ*/ppm = 4.11–4.00 (m, 2H), 2.60–2.50 (m, 1H), 1.76–1.67 (m, 1H), 1.61 (q, *J* = 6.6 Hz, 3H), 1.52–1.44 (m, 3H), 1.40–1.15 (m, 49H), 1.14 (d, *J* = 6.9 Hz, 3H), 1.10–0.90 (m, 5H), 0.90–0.79 (m, 22H). ^13^C-NMR, DEPT: (151 MHz, CDCl_3_) *δ*/ppm = 177.3 (C_q_), 64.4 (CH_2_), 45.5 (CH_2_), 41.6 (CH_2_), 37.7 (CH), 37.1 (CH_2_), 32.1 (CH_2_), 32.1 (CH_2_), 30.2 (CH_2_), 30.0 (CH), 29.9 (CH_2_), 29.9 (CH_2_), 29.8 (CH_2_), 29.8 (CH_2_), 29.7 (CH_2_), 29.7 (CH_2_), 29.5 (CH_2_), 29.5 (CH_2_), 29.4 (CH_2_), 28.9 (CH_2_), 28.4 (CH), 27.1 (CH_2_), 26.1 (CH_2_), 22.9 (CH_2_), 22.8 (CH_2_), 20.4 (CH_3_), 20.3 (CH_3_), 18.4 (CH_3_), 14.3 (CH_3_). EI-MS (70 eV): *m/z* (%) = 452 ([M]^+^, 1%), 299 (10), 241 (10), 154 (15), 87 (89), 85 (31), 75 (37), 74 (63), 71 (50), 69 (50), 57 (100), 55 (55), 43 (93), 41 (45). HR-CIP-MS: m/z = 451.45132 [M–H]^+^. *theo*.: 451.45096. For NMR spectra see Figure S64.

##### Preparation of (2*S*,4*S*,6*S*)-2,4,6-trimethylhexadecyl (2S,4S,6S)-2,4,6-trimethylhexadecanoate (5)

*N*,*N*′-dicyclohexylcarbodiimide (15 mg, 0.070 mmol, 1.0 eq.) was added to a solution of (2*S*,4*S*,6*S*)-2,4,6-trimethylhexadecanoic acid (**34**) (20 mg, 0.070 mmol, 1.0 eq.), (2*S*,4*S*,6*S*)-2,4,6-trimethylhexadecan-1-ol (**33**) (21 mg, 0.070 mmol, 10.0 eq.), and 4-dimethylaminopyridine (0.9 mg, 0.007 mmol, 0.1 eq.) in CH_2_Cl_2_ (0.8 mL) The mixture was stirred for 16.5 h at room temperature. The solvent was removed and the residue was purified by flash chromatography (pentane) to obtain **5** as a colorless oil (35 mg, 0.062 mmol, 88%). ${\left[\alpha \right]}_{D}^{25}$ = −2.6 (10 mg/mL; CHCl_3_). FT-IR: *ν*/cm^−1^ = 2957, 2923, 2853, 1737, 1462, 1377, 1257, 1174, 1085, 981, 723, 596, 555. ^1^H–NMR: (300 MHz, CDCl_3_) *δ*/ppm = 4.01–3.76 (m, 2H), 2.64–2.48 (m, 1H), 1.89 (td, *J* = 13.7, 6.9 Hz, 1H), 1.79–1.66 (m, 1H), 1.66–1.40 (m, 5H), 1.37–1.10 (m, 44H), 1.11–0.97 (m, 5H), 0.96–0.76 (m, 27H). ^13^C-NMR, DEPT: (76 MHz, CDCl_3_) *δ*/ppm = 177.3 (C_q_), 69.1 (CH_2_), 45.5 (CH_2_), 45.4 (CH_2_), 41.6 (CH_2_), 41.6 (CH_2_), 37.8 (CH_3_), 37.1 (CH_2_), 36.7 (CH_2_), 32.1 (CH_2_), 30.2 (CH), 30.2 (CH), 30.1 (CH), 30.0 (CH_2_), 29.9 (CH_2_), 29.9 (CH_2_), 29.8 (CH_2_), 29.5 (CH_2_), 28.4 (CH), 27.6 (CH), 27.1 (CH_2_), 22.9 (CH_3_), 20.9 (CH_3_), 20.6 (CH_3_), 20.4 (CH_3_), 20.3 (CH_3_), 18.4 (CH_3_), 18.2 (CH_3_), 14.3 (CH_3_). EI-MS (70 eV): *m/z* (%) = 355 (<1), 341 (<1), 299 (7), 281 (4), 207 (15), 125 (23), 85 (40), 83 (43), 71 (58), 69 (51), 57 (100), 56 (23), 55 (50), 43 (81), 41 (37). HR-CIP-MS: *m/z* = 563.57635 [M–H]^+^. *theo*.: 563.57616. For NMR spectra see Figure S63.

##### Preparation of (*S*)-3-methylcyclopent-1-ene (45)

Benzylidene-bis(tricyclohexylphosphino)-dichlororuthenium (Grubbs cat.) was added to a stirred solution of (*S*)-citronellene (**44**, 10 mL, 7.6 g, 55 mmol) in CH_2_Cl_2_ (100 mL). The resulting solution was heated to reflux for 18 h. The product was separated from the catalyst by distillation and was obtained as a solution in CH_2_Cl_2_.^44^^,^^87^^,^^88^ The yield was 84% (GC). EI-MS (70 eV)**:** *m/z* (%) = 82 (23), 81 (11), 67 (100), 65 (10), 53 (7), 51 (5), 41 (18), 39 (22).

##### Preparation of (5*S*)-1,5-dimethyl-tricyclo[5.3.0.02.6]decan-8-one (47)

A mixture of the crude (*S*)-3-methylcyclopent-1-ene (**45**) and 3-methylcyclopent-2-en-1-one (**46**) (5 mL, 4.85 g, 50 mmol) were dissolved in CH_2_Cl_2_ (150 mL) and placed in a photoreactor (150 W). The solution was stirred for 3 h and the solvent was removed under reduced pressure.^44^^,^^89^ The residue was purified by flash chromatography (pentane/Et_2_O; 9:1) to obtain **47** as a mixture of isomers (3040 mg, 17 mmol, 34%). EI-MS (70 eV): *m/z* (%) = 178 (22), 163 (14), 160 (8), 149 (5), 145 (11), 136 (14), 121 (15), 107 (17), 97 (97), 93 (28), 91 (21), 82 (19), 81 (25), 79 (35), 77 (24), 67 (100), 55 (17), 53 (25), 41 (37), 39 (35). HR-EI-MS: *m/z* = 178.1359 [M–H]^+^. *theo*.: 178.1358. ^1^H-NMR: (400 MHz, CDCl_3_) *δ*/ppm = 2.71–2.62 (ddd, *J* = 17.5, 9.2, 0.8 Hz, 1H), 2.56–2.41 (m, 2H), 2.36–2.28 (ddd, *J* = 9.6, 6.4, 1.6 Hz, 1H), 2.07 (d, *J* = 3.4 Hz, 1H), 2.04–1.97 (m, 1H), 1.96–1.85 (m, 2H), 1.82–1.74 (m, 2H), 1.60–1.51 (m, 1H), 1.33–1.23 (m, 1H), 1.00 (d, *J* = 6.7 Hz, 3H), 0.93 (s, 3H). ^13^C-NMR, DEPT: (100 MHz, CDCl_3_) *δ*/ppm = 221.8 (C_q_), 50.0 (CH), 47.4 (CH), 44.7 (CH), 39.4 (CH), 38.1 (CH_2_), 37.8 (CH_2_), 37.2 (CH), 33.6 (CH_2_), 27.8 (CH_2_), 20.3 (CH_3_), 13.8 (CH_3_).
