# Supplementary material for: The unique epicuticular chemistry of Collembola – A cross-species analysis
Source: iScience. 2024 Jun 28;27(8):110416. doi: 10.1016/j.isci.2024.110416 (PMC11321324; doi:10.1016/j.isci.2024.110416)
Supplement: Document S1. Figures S1–S64 and Table S1 [file mmc1.pdf]

iScience, Volume *27*

Supplemental information

The unique epicuticular chemistry of Collembola – A cross-species analysis

Anton Möllerke, Gregor Brasse, Jan Bello, Diogo Montes Vidal, Konrad Dettner, Jürg Zettel, Matty P. Berg, Stefan Scheu, Hans Petter Leinaas, and Stefan Schulz

Table S1. Overview of the compounds found in extracts of 23 Springtail species. Major components of the lipids of a species are shown in bold, except in species with only minor amounts of lipids. sq = squalene (**10**), ch = cholesterol, desmo = desmosterol. Long-chain alkanes and alkenes are described by their total carbon number, e.g. C_{20} for eicosane, related to Figure 4.

Order	Family	Species	Cuticular lipids		
			Fatty acid pathway	Terpene pathway	
				steroids	non-steroids
Poduromorpha	Neanuridae	*Anurida maritima*	**2,4,6-trimethylhexadecyl 2,4,6-trimethylhexadecanoate** (**1**)<br>2,4,6-trimethyltridecyl 2,4,6-trimethylhexadecanoate<br>2,4,6-trimethyltetradecyl 2,4,6-trimethylhexadecanoate	ch	sq (**10**)
	Hypogastruridae	*Ceratophysella denticulata*		ch, desmo	sq (**10**), **lycopane** (**11**), lycopaene (**12**), lycopadiene (**13**), diploptene (**16**)
		Ceratophysella sigillata		ch, desmo, cholestanol	sq (**10**), **lycopane** (**11**), **lycopaene** (**12**), lycopadiene (**13**), diploptene (**16**)
		Hypogastrura socialis	C_{21}, C_{23}, C_{25}, C_{27}	ch, cholest-3,5-diene, steroid	sq (**10**), **socialane** (**17**), dihydrosocialane
		Hypogastrura viatica		ch, desmo, ergosta-5,22-dien-3-ol	sq (**10**), **viaticene A** (**8**) and B
		Hypogastrura vernalis	C_{25}, C_{27}, C_{31}	ch, cholest-3,5-diene	sq (**10**), **viaticene A** (**8**) and B
		Xenylla grisea	C_{28}, C_{29}, C_{30}	ch	sq (**10**), **poduran**-derivatives **20,22–24**
		Xenylla maritima	**1-methoxy-2,4,6,8,10,12,14-heptamethyloctacosane** (**6**)	ch	sq (**10**), **[8]-terpene**
	Poduridae	*Podura aquatica*		ch, desmo, cholesteryl acetate	sq (**10**), **poduran** (**18**), tetrahydropentaprenylprespatane (**19**)
	Onychiuridae	*Protaphorura fimata*		ch, desmo	**sq** (**10**), **dihydrogeranylfarnesylfarnesene** (**7**) and derivatives
		Tetrodontophora bielanensis[1, 2]	C_{15}–C_{35}, C_{40}–C_{51}, long-chain esters (C_{18} acids), secondary alcohol ($C_{40}H_{82}O$)[3]	ch, desmo	lycopane (**4**), lycopaene (**5**), lycopadiene (**6**)
		Onychiurus fimatus		**ch, desmo**	sq (**10**), **7**
		Megaphorura arctica		ch, desmo	sq (**10**), tocopherol (**14**)
Ento	Isotomidae	*Anurophorus laricis*	hentetracosadiene	**ch**, cholest-3,5-diene	sq (**10**)

Order	Family	Species	Cuticular lipids		
			**Fatty acid pathway**	**Terpene pathway**	
				**steroids**	**non-steroids**
		Folsomia quadrioculata	hexatriacontatetraene	**ch**	sq (**10**), sclareol (**15**), **two tetraterpenes**
		Folsomia candida		**ch**	sq (**10**), socialane-type terpenes (**21**), and other terpenoids
		Vertagopus sarekensis	**sarekensane** (**1**)**,** C_{23}–C_{32}	ch	sq (**10**)
		Cryptopygus clavatus	Unknown compounds	**ch**, cholest-7-en-3-ol	**sq** (**10**), tocopherol (**14**)
	Entomobryidae	*Heteromurus nitidus*		ch	sq (**10**), **nitidane** (**9**), and related [8]- and [9]-terpenes.
		Orchesella cincta	**3,11,23-trimethyltritriacont-1-ene** (**3**), **9,29-dimethylpentatriacont-1-ene** (**2**)	**ch**	sq (**10**)
		Sinella curviseta	**pentamethylhentriacontahexanene**	ch	sq (**10**)
	Tomoceroidae	*Tomocerus vulgaris*	unknown ester ($C_{28}H_{46}O_2$), C_{27}-diene, C_{27}-ene, C_{29}-ene, 9,17-dimethylpentatriacontane (**4**)	**ch**, desmo	sq (**10**)
Symphypleona	Sminthurididae	*Sminthurides aquaticus*	octadecyl acetate	ch	

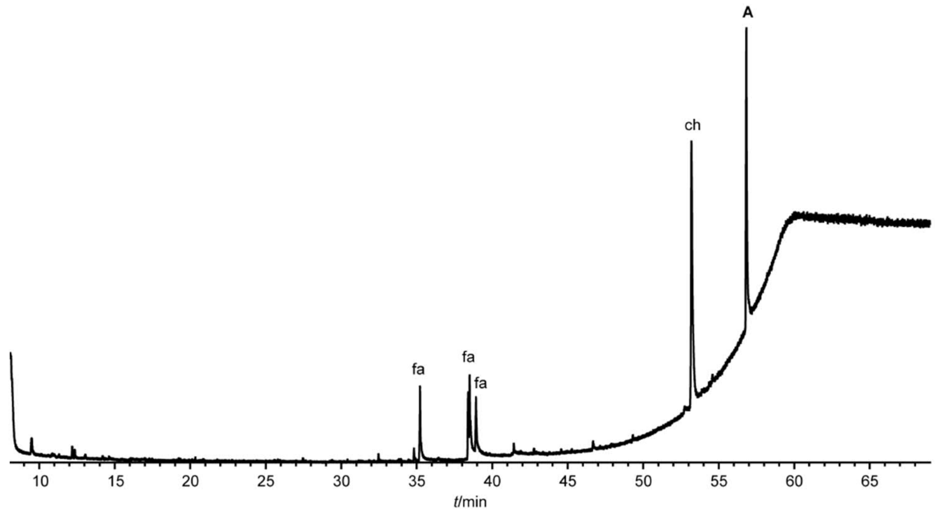

Figure S1. TIC of a pentane extract of *Anurida maritima* (temp. A). **A**= 2,4,6-trimethylhexadecyl 2,4,6-trimethylhexadecanoate (**5**), related to Figure 1.

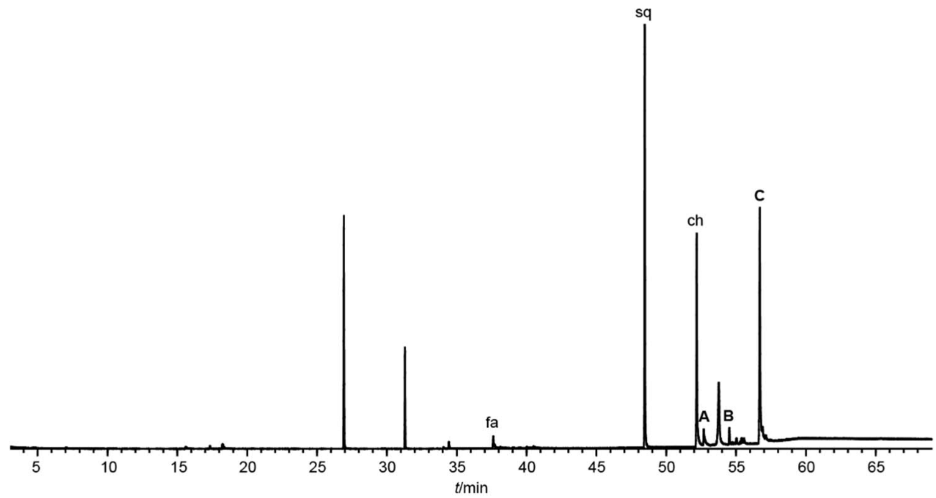

Figure S2. TIC of the pentane extract of *Ceratophysella denticulata* (temp. A). **A**= desmosterol, **B**= diploptene (**16**), **C**= lycopane (**11**), related to Figure 1.

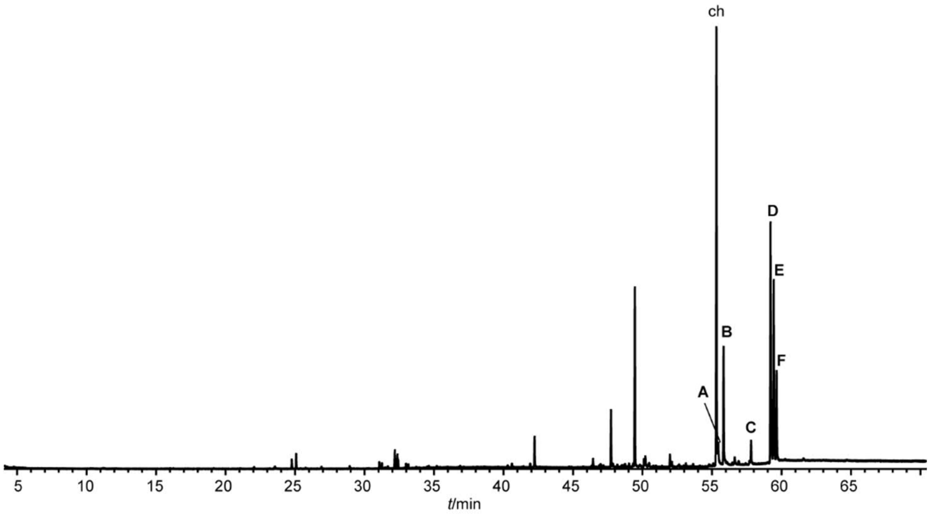

Figure S3. TIC of the pentane extract of *Ceratophysella sigillata* (temp. A). **A**= cholestenol, **B**= desmosterol, **C**= diploptene (**16**), **D**= lycopane (**11**), **E**= lycopaene (**12**), **F**= lycopadiene (**13**), related to Figure 1.

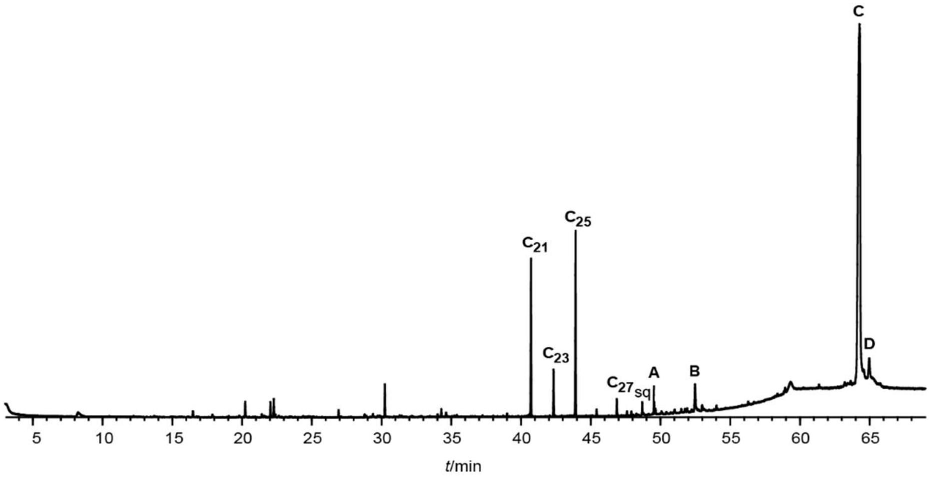

Figure S4. TIC of the pentane extract of *Hypogastrura socialis* (temp. A). **A**= cholest-3,5-diene, **B**= ch, **C**= socialane (**17**), **D**= dihydro-socialane, related to Figure 1.

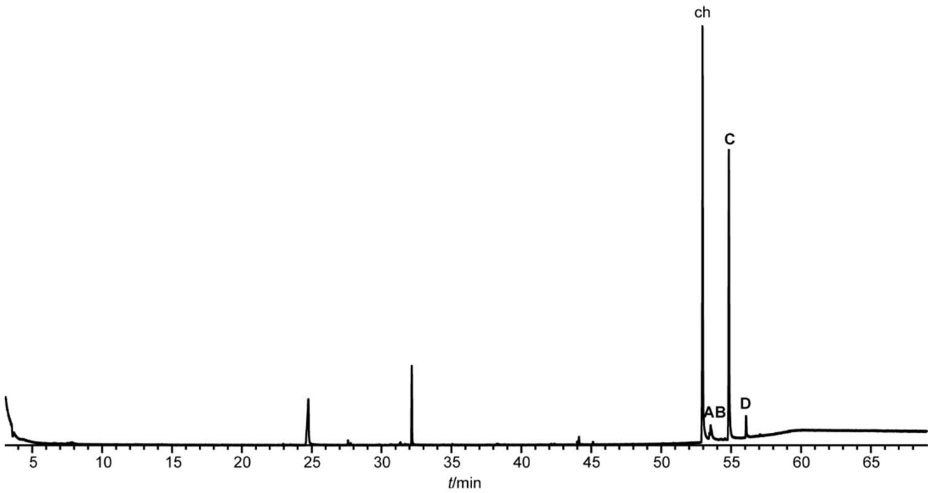

Figure S5. TIC of the pentane extract of *Hypogastrura viatica* (temp. A). **A**= desmosterol, **B**= ergosta-5,22-dien-3-ol, **C**= viaticene A (**8**), **D**= viaticene B, related to Figure 1.

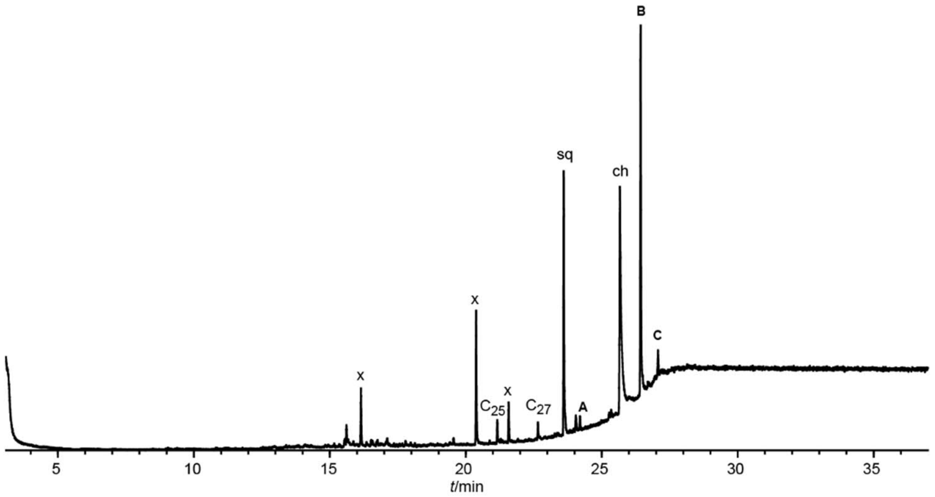

Figure S6. TIC of the pentane extract of *Hypogastrura vernalis* (temp. B). **A**= cholest-3,5-diene, **B**= viaticene A (**8**), **C**= viaticene B, related to Figure 1.

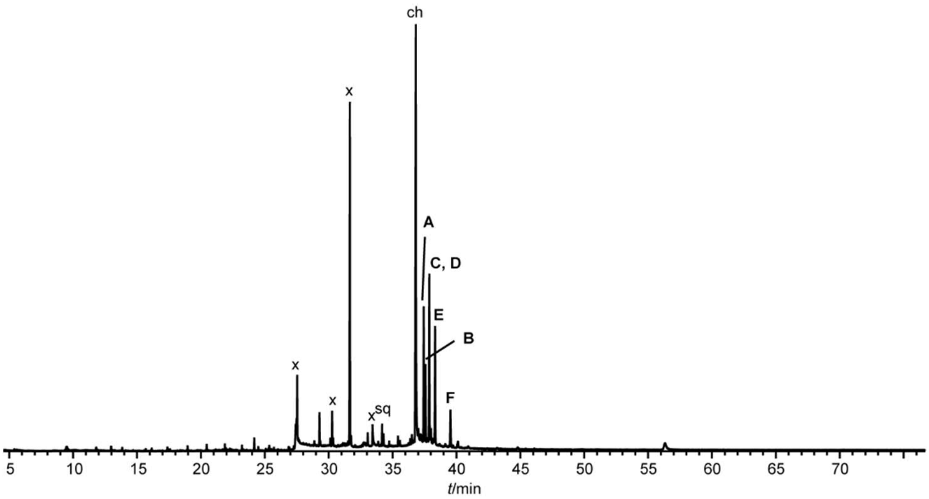

Figure S7. TIC of the CH_2Cl_2 extract of *Xenylla grisea* (temp. C). **A**= **22**, **B**= **23**, **D**= **21**, **E**= **24**, **C** and **F**= unknown terpenes, related to Figure 1.

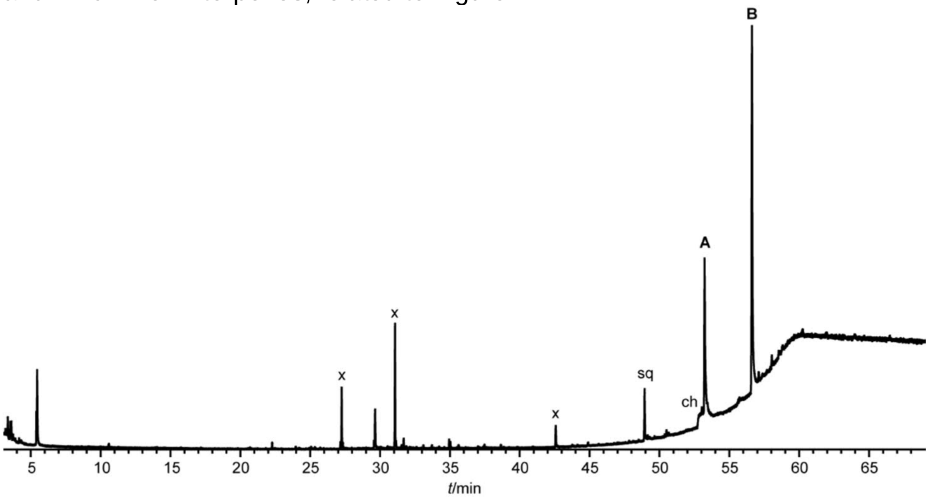

Figure S8. TIC of the pentane extract of *Xenylla maritima* (temp. A). **A**= 1-methoxy-2,4,6,8,10,12-hexamethylnonacosane (**6**), **B**= [8]-terpene, related to Figure 1.

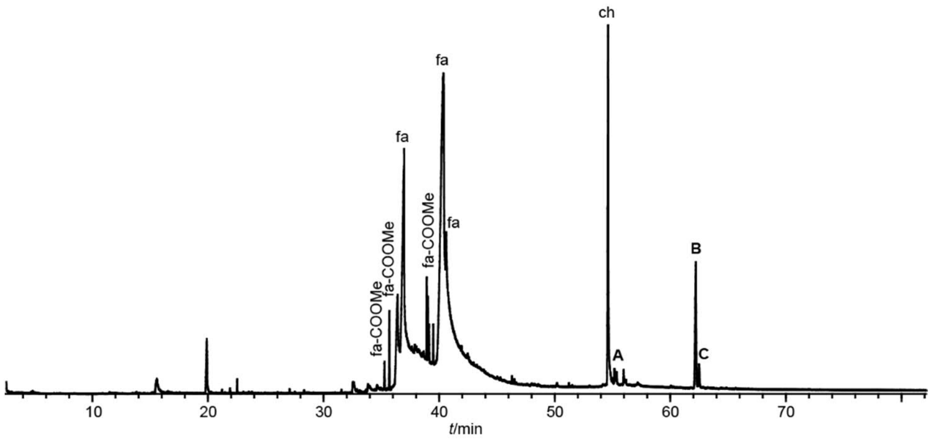

Figure S9. TIC of the MeOAc extract of *Podura aquatica* (temp. C). **A**= cholesteryl acetate, **B**= poduran (**18**), **C**= decahydropentaprenylprespatane (**19**), related to Figure 1..

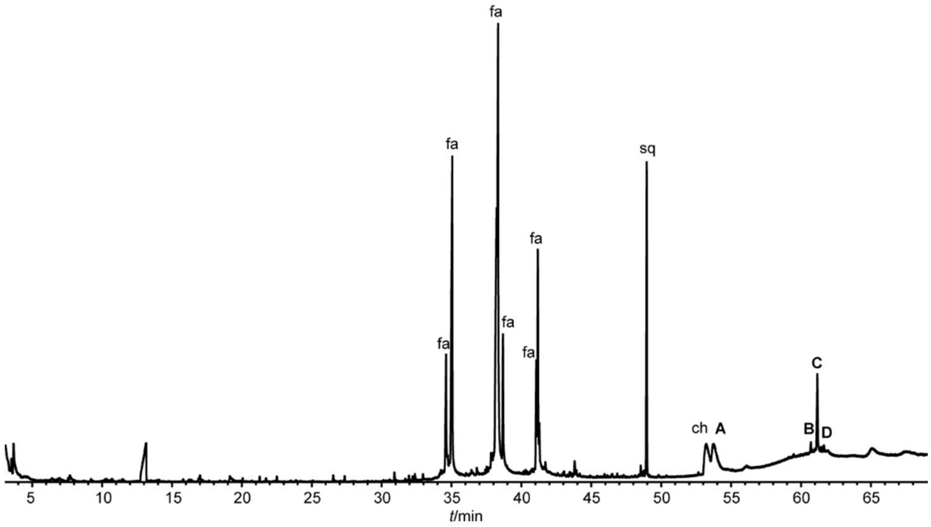

Figure S10. TIC of the CH_2Cl_2 extract of *Protaphorura fimata* (temp. A). **A**= desmosterol, **C**= dihydrogeranylfarnesylfarnesene (**7**), **B**, **D**= derivatives of **7**, related to Figure 1.

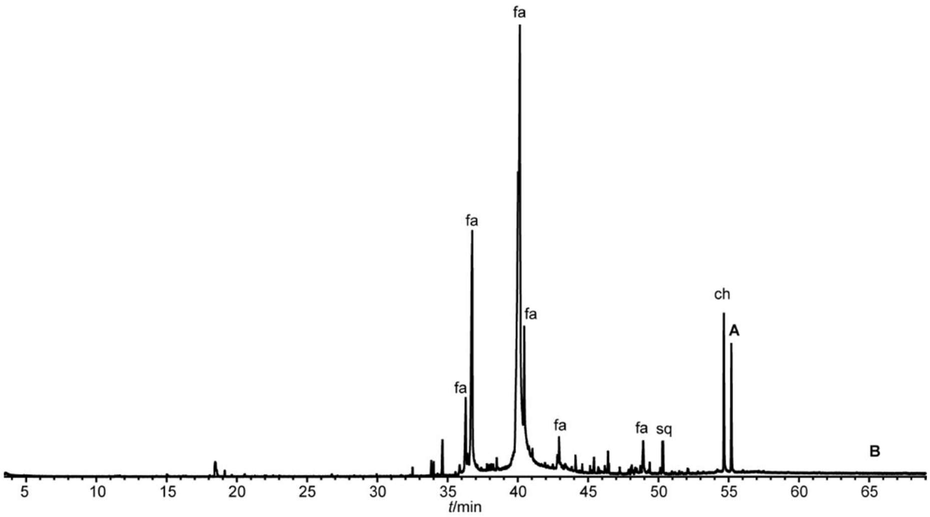

Figure S11. TIC of the CH_2Cl_2 extract of *Onychiurus fimatus* (temp. A). **A**= desmosterol, **B**= **7**, related to Figure 1.

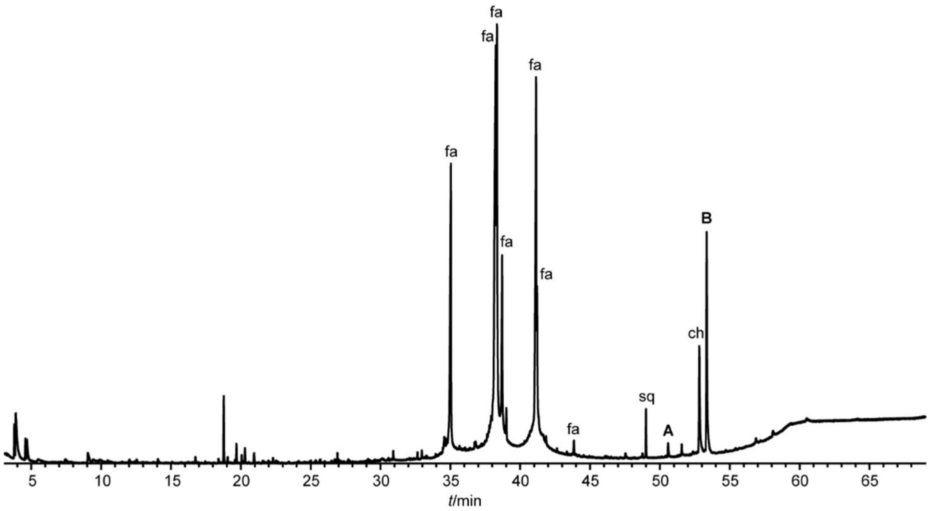

Figure S12. TIC of the CH_2Cl_2 extract of *Megaphorura arctica* (temp. A). **A**= tocopherol (**15**), **B**= desmosterol, related to Figure 1.

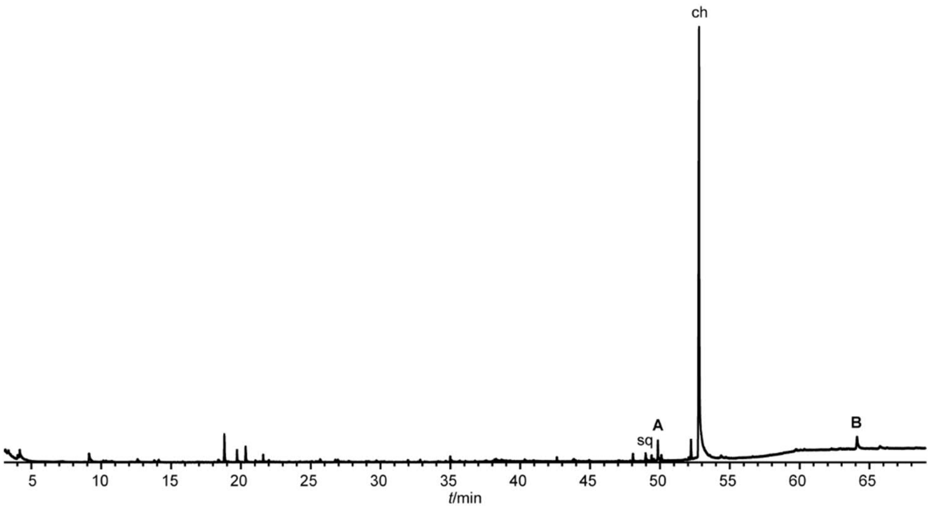

Figure S13. TIC of the CH_2Cl_2 extract of *Anurophorus laricis* (temp. A). **A**=cholest-3,5-diene, **B**= hentetracosadiene, related to Figure 1.

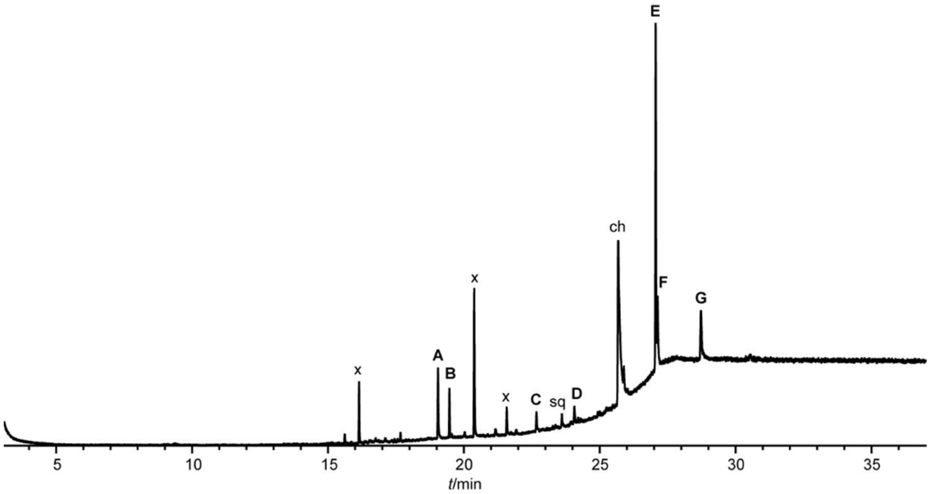

Figure S14. TIC of the pentane extract of *Folsomia quadrioculata* (temp. B). **A**= sclareol (**16**), **B**= unknown, **C, D**= unknown terpenes, **E, F**= unknown [8]-terpene, **G**= hexatriacontatetraene, related to Figure 1.

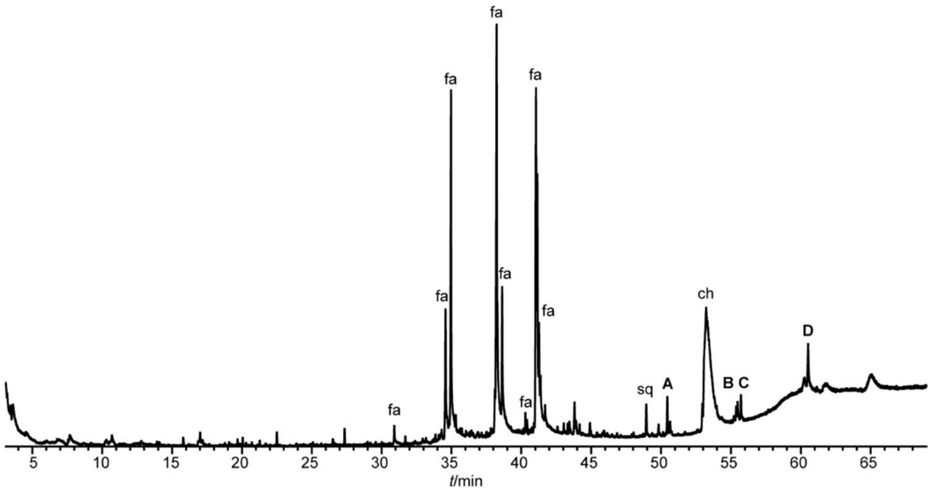

Figure S15. TIC of the CH_2Cl_2 extract of *Folsomia candida* (temp. A). **A**, **B**= unknown, **C, D, E,** = unknown socialane type cyclized linear terpene (**21**), related to Figure 1.

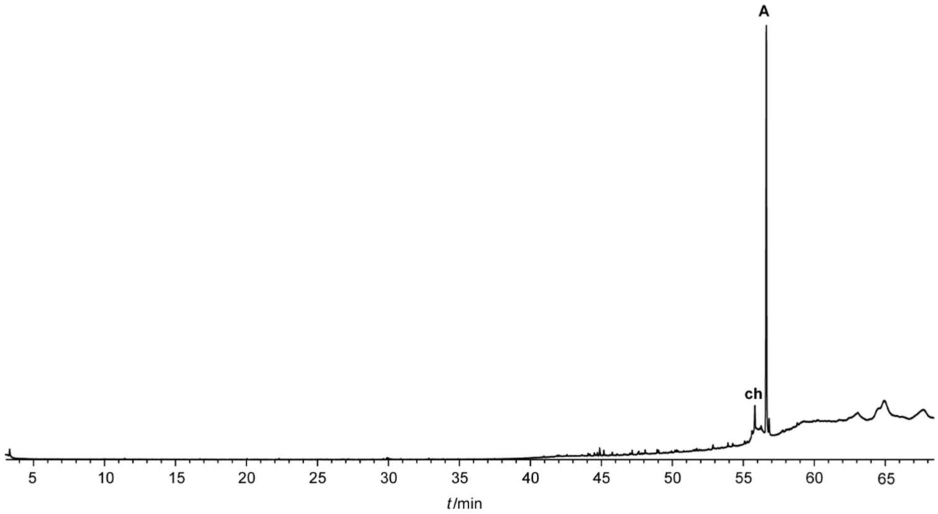

Figure S16. TIC of the pentane extract of *Vertagopus sarekensis* (temp. A). **A**= sarekensane (**1**), related to Figure 1.

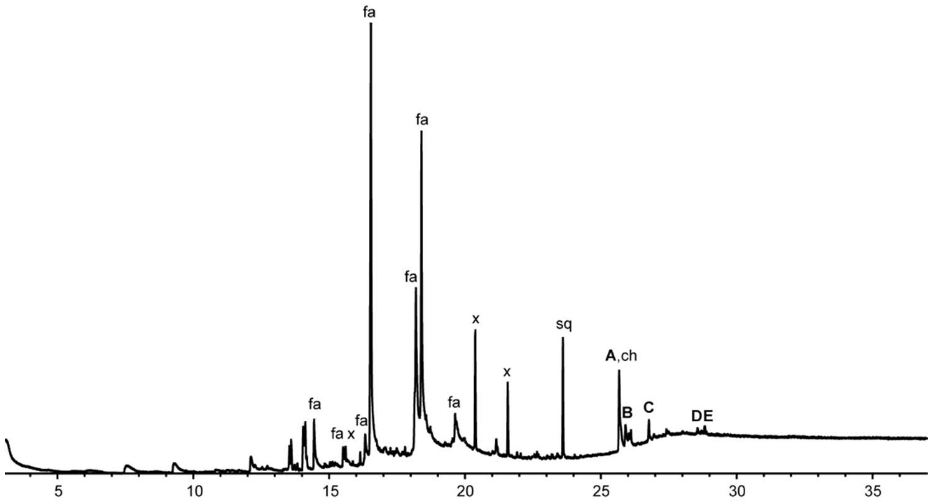

Figure S17. TIC of the CH_2Cl_2 natural extract of *Cryptopygus clavatus* (temp. B). **A**= tocopherol (**15**), **B**= cholesta-7-en-3-ol, **C**, **D**, **E**= unknown aliphatic compounds, related to Figure 1.

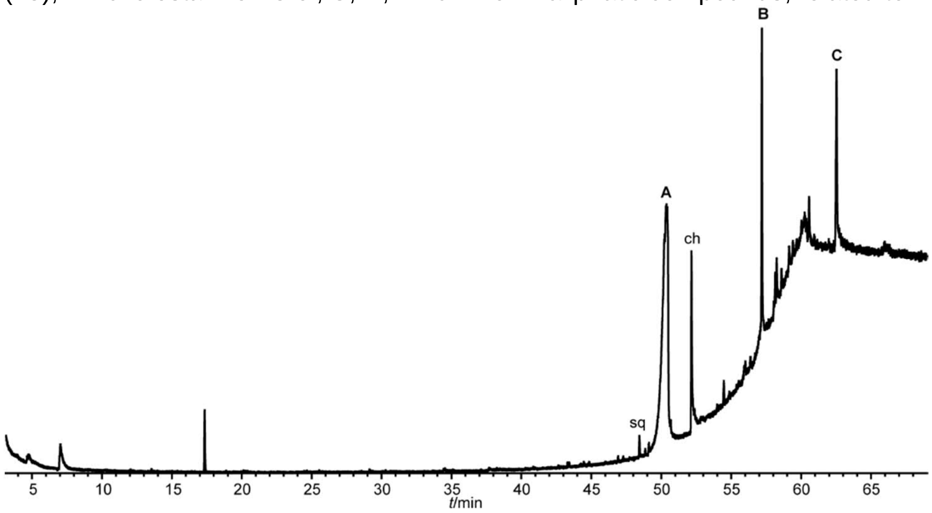

Figure S18. TIC of the pentane extract of *Heteromurus nitidus* (temp. A). **A**= nitidane (**9**), **B**= unknown [8]-terpene, **C**= unknown [9]-terpene, related to Figure 1.

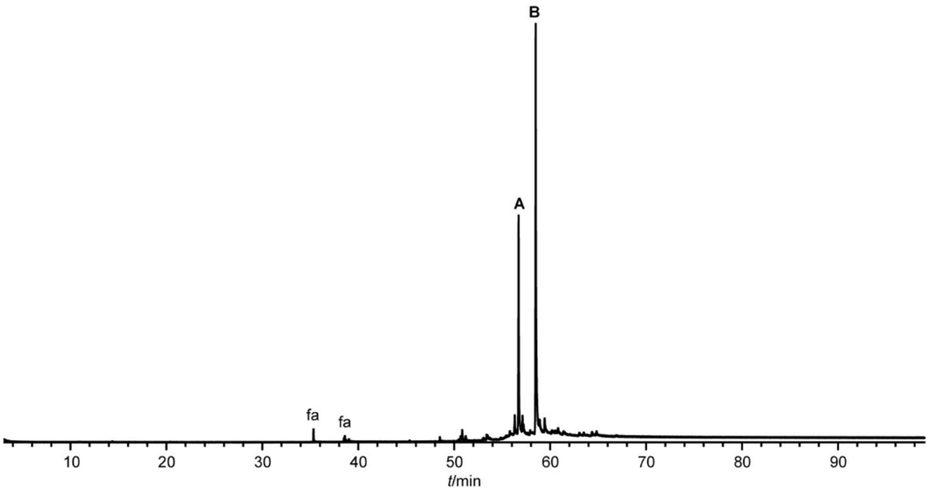

Figure S19. TIC of the pentane extract of *Orchesella cincta* (temp. A). **A**= 3,11,23-trimethyltritriacont-1-ene (**3**), **B**= 9,29-dimethylpentatriacont-1-ene (**2**), related to Figure 1.

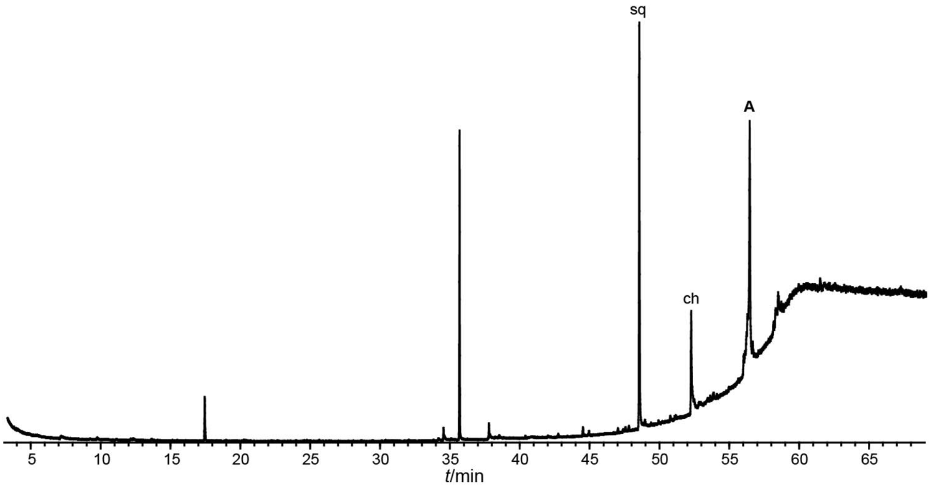

Figure S20. TIC of the pentane extract of *Sinella curviseta* (temp. A). **A**= pentamethylhentriacontahexaene, related to Figure 1.

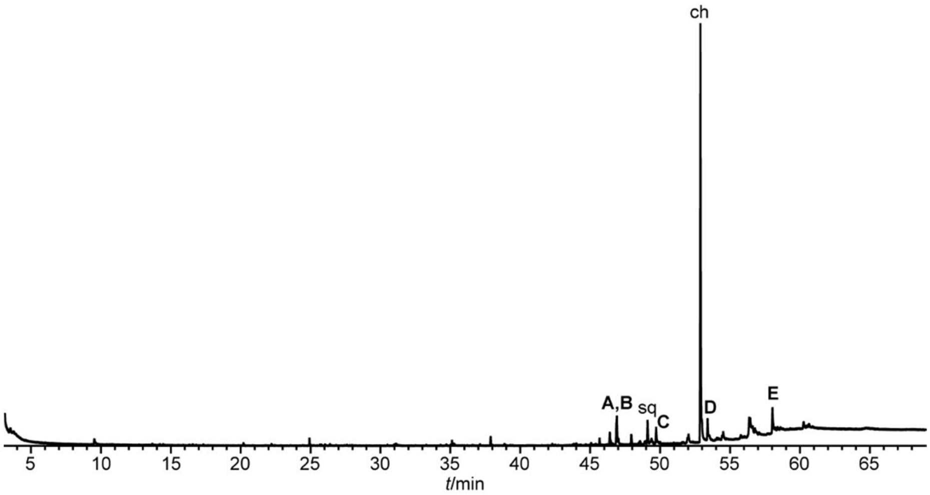

Figure S21. TIC of the pentane extract of *Tomocerus vulgaris* (temp. A). **A**= C_{27}-diene, **B**= C_{27}-ene, **C**= C_{29}-ene, **D**= desmosterol, **E**= 9,17-dimethylheptatriacontane (**4**), related to Figure 1.

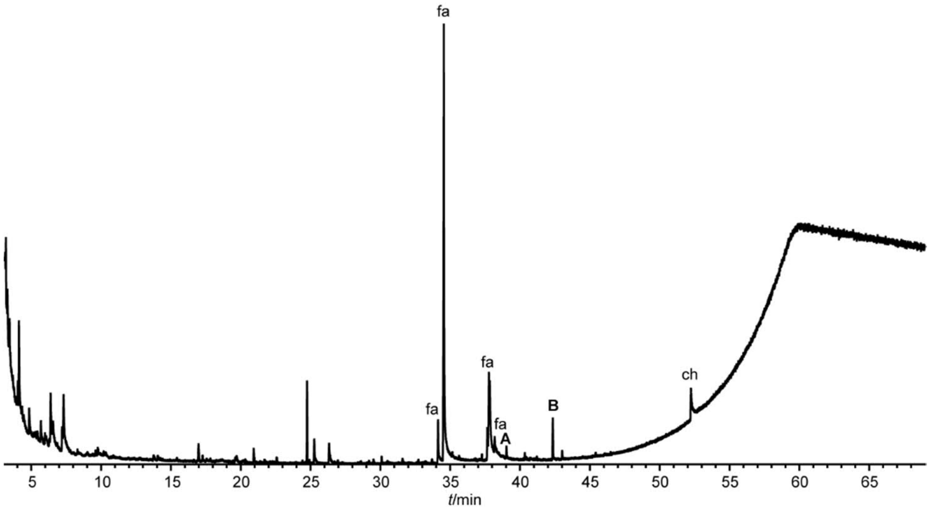

Figure S22. TIC of the CH_2Cl_2 extract of *Sminthurides aquaticus* (temp. A). **A**= C_{18}OAc, **B**= C_{20}OAc, related to Figure 1.

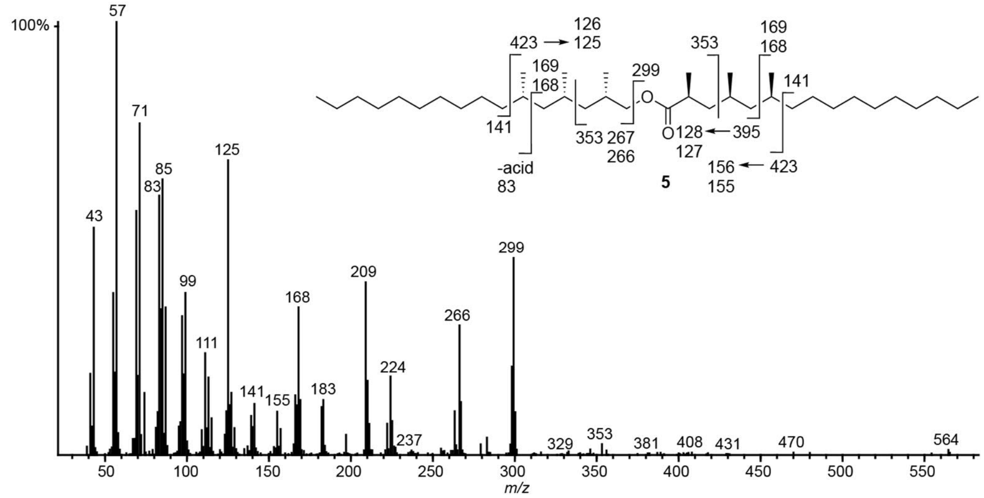

Figure S23. Mass spectrum of the natural compound **ANM-A** from *A. maritima,*related to STAR methods.

.

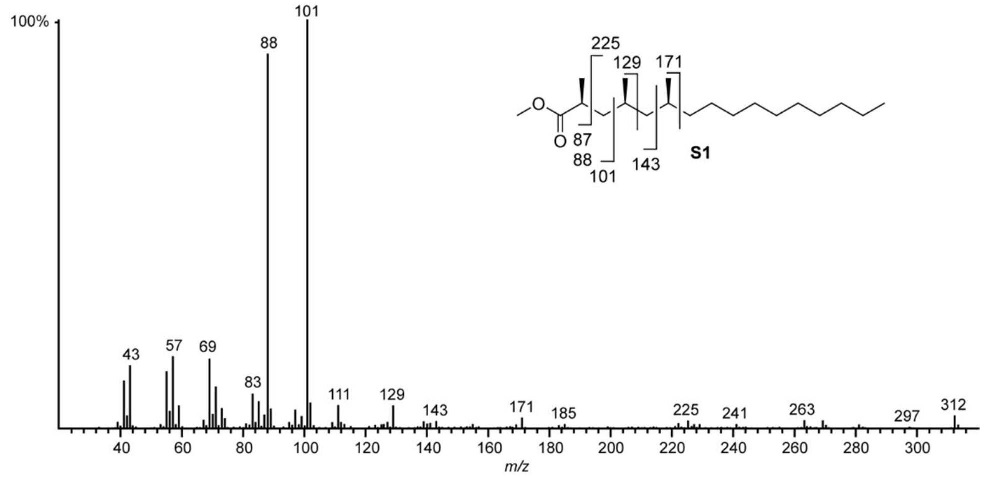

Figure S24. Mass spectrum of the methyl ester obtained by transesterification with TMSH from **ANM-A**, related to STAR methods.

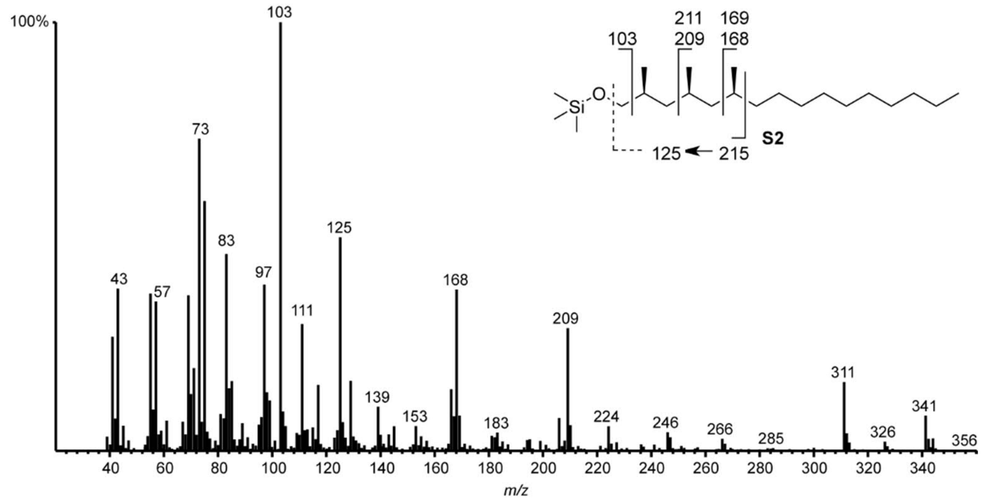

Figure S25. Mass spectrum of the silyl ether **S2** obtained by micro derivatization with MSTFA after transesterification of **ANM-A,** related to STAR methods.

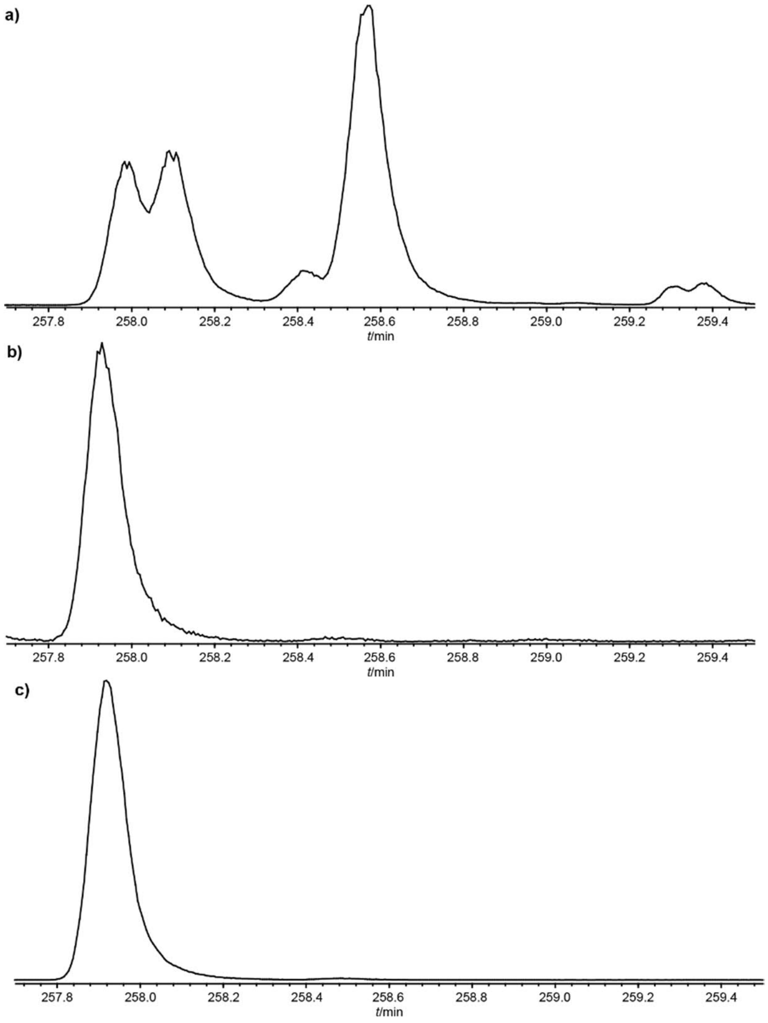

Figure S26. Selected ion chromatogram of *m/z* 101 of the methyl ester of **a)** (2*R**,4*R**,6*R**)- and (2*R**,4*R**,6*S**)-diastereomers of **34**, **b)** methyl ester of **ANM-A**, **c)** methyl esters of **ANM-A** and (2*S*,4*S*,6*S*)-**34** coinjected. Separation was performed using a Hydrodex β-6TBDM phase (30.0 m × 0.25 mm, 1.5 mL/min He, initial temp. 50 °C then 10 °C min^{-1} to 125 °C holding time for 240 min, then with 10 °C min^{-1} to final temp. 230 °C**,** related to STAR methods.

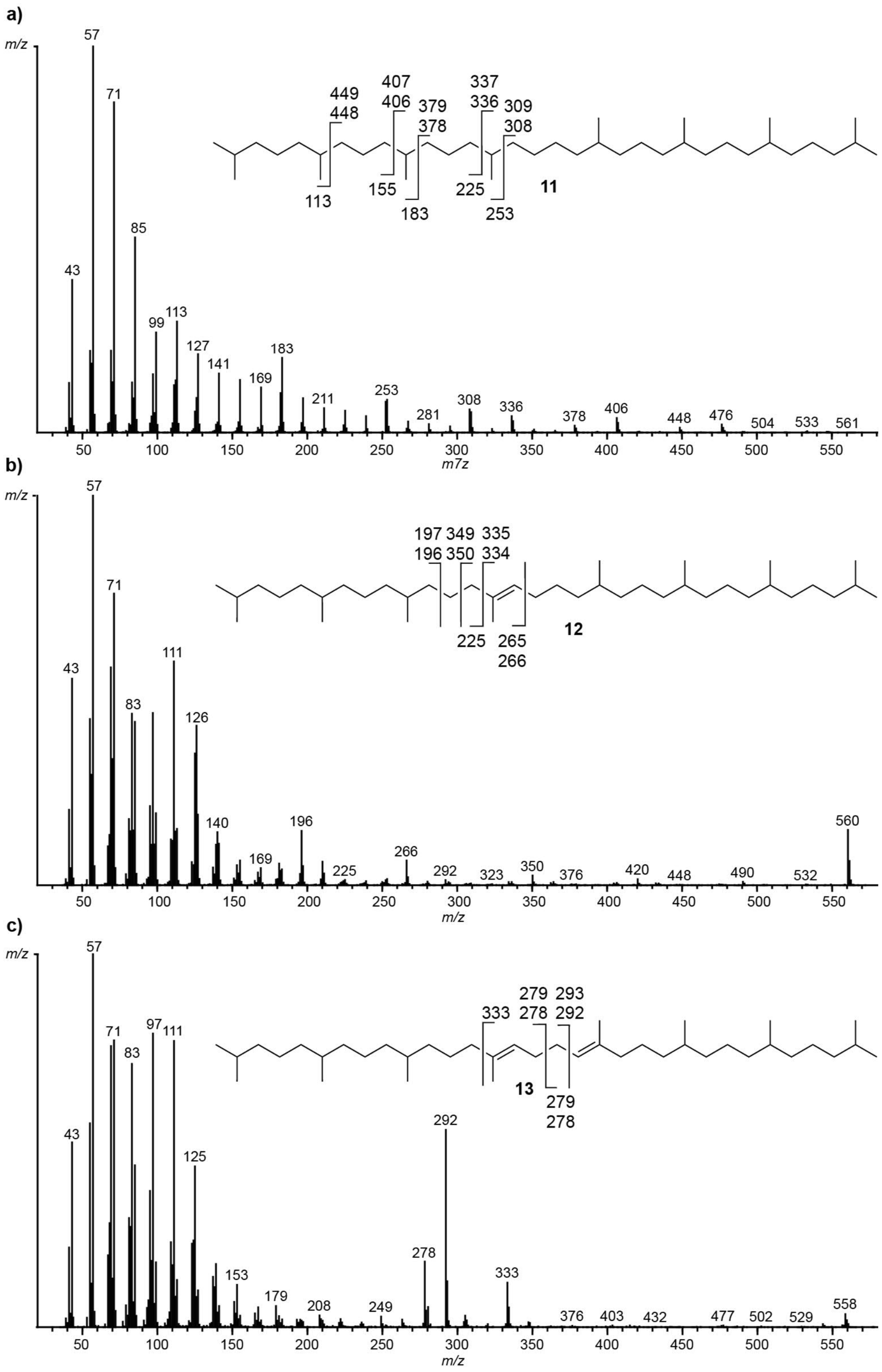

Figure S27. Mass spectrum of the natural compound lycopane (**11**) (a), lycopaene (**12**) (b), and lycopadiene (**13**) (c)**,** related to STAR methods.

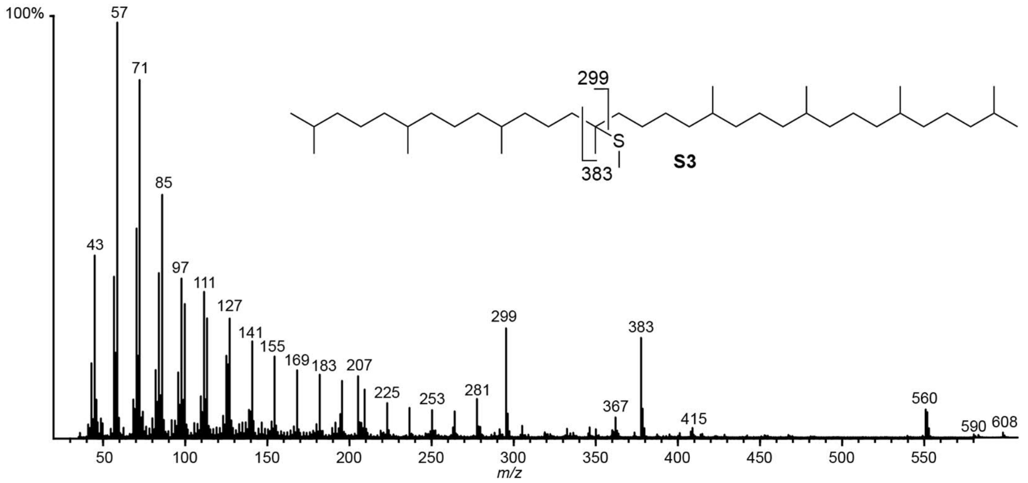

Figure S28. Mass spectrum of **S3**, the product of the DMDS derivatization of lycopaene (**12**), related to STAR methods.

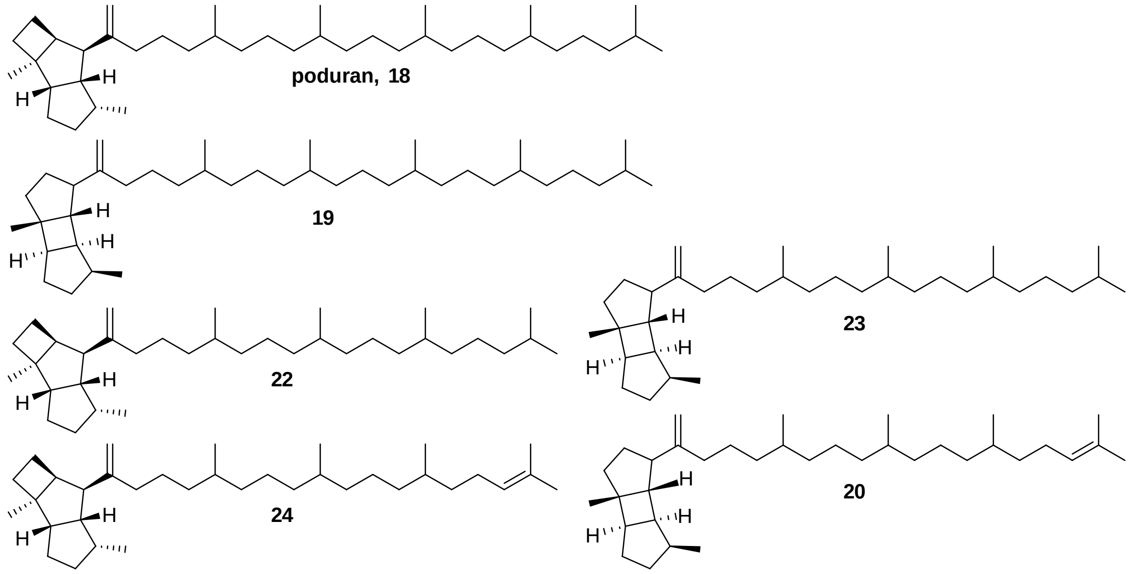

Figure S29. Poduran (**18**) and its analogs (**19**, **20**, **22**–**24**) from *P. aquatica* and *X. grisea*, related to STAR methods.

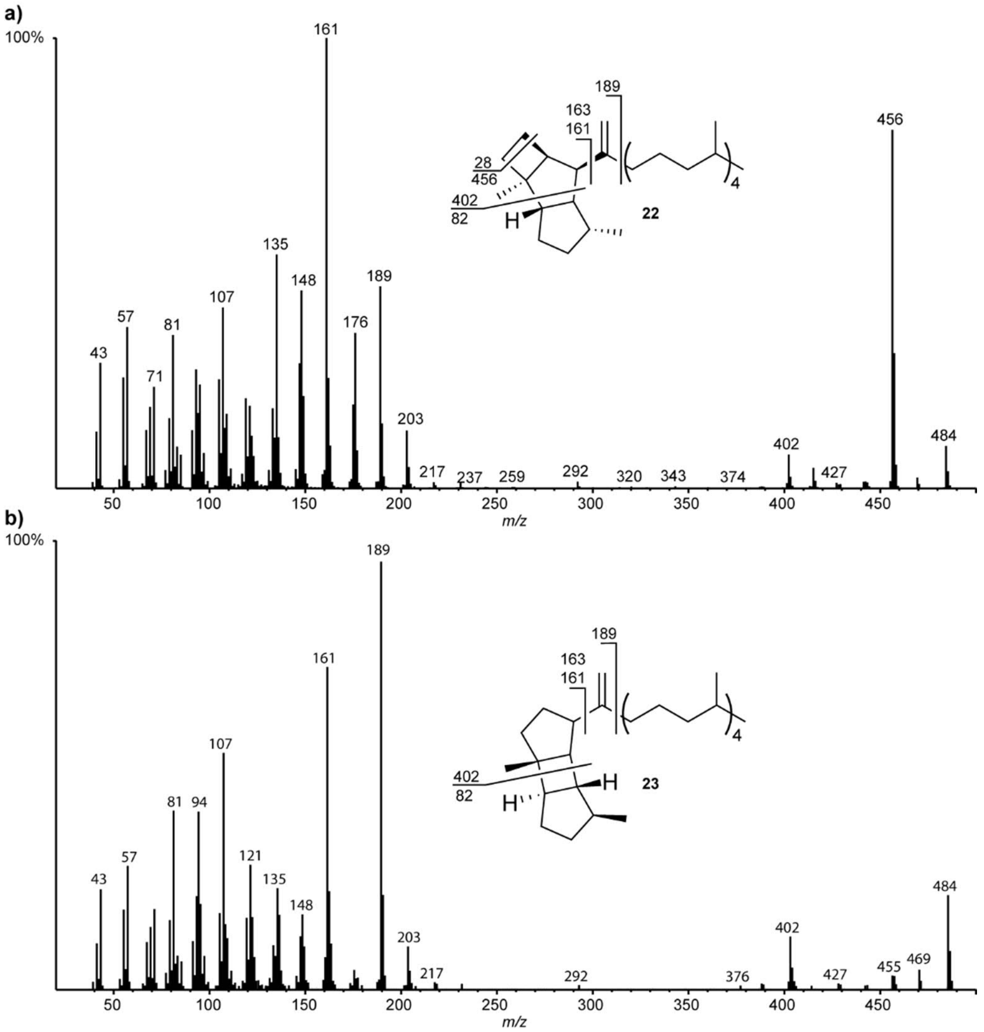

Figure S30. Mass spectra of the natural compounds ocathydrotetraprenylkelsoene (**22**) (a) and octahydrotetraprenylprespatane (**23**) (b) from *X. grisea*, related to STAR methods.

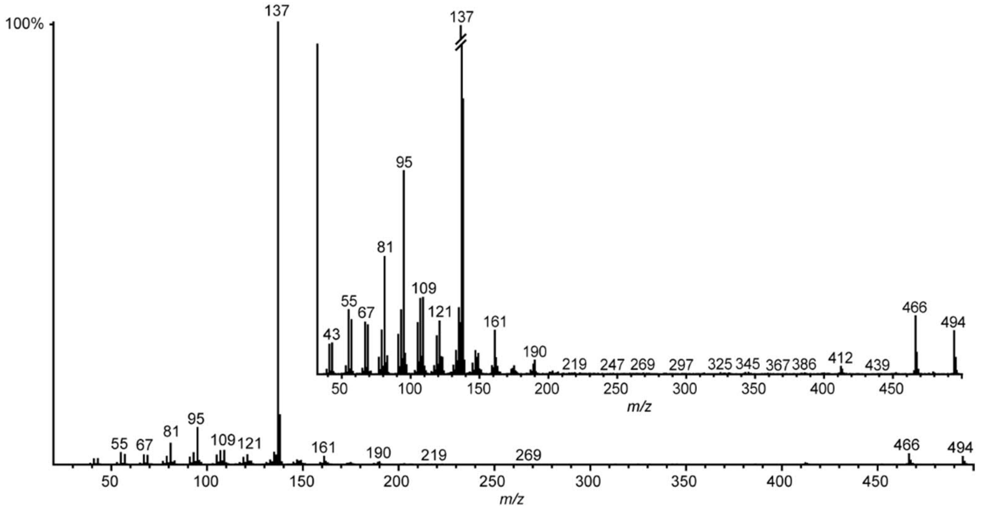

Figure S31. Mass spectrum of the natural compound **XGR-F** from *X. grisea***,** related to STAR methods.

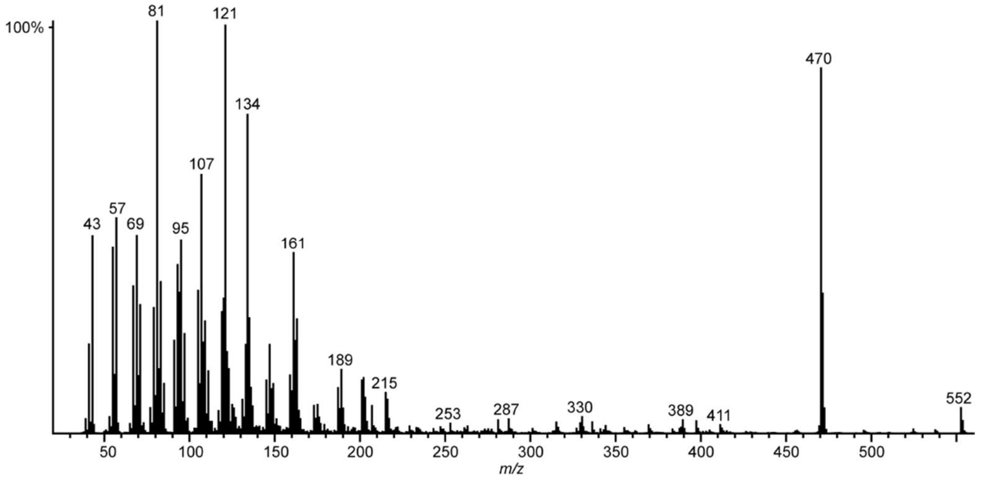

Figure S32. Mass spectrum of the natural compound **XEM-B** from *X. maritima***,** related to STAR methods.

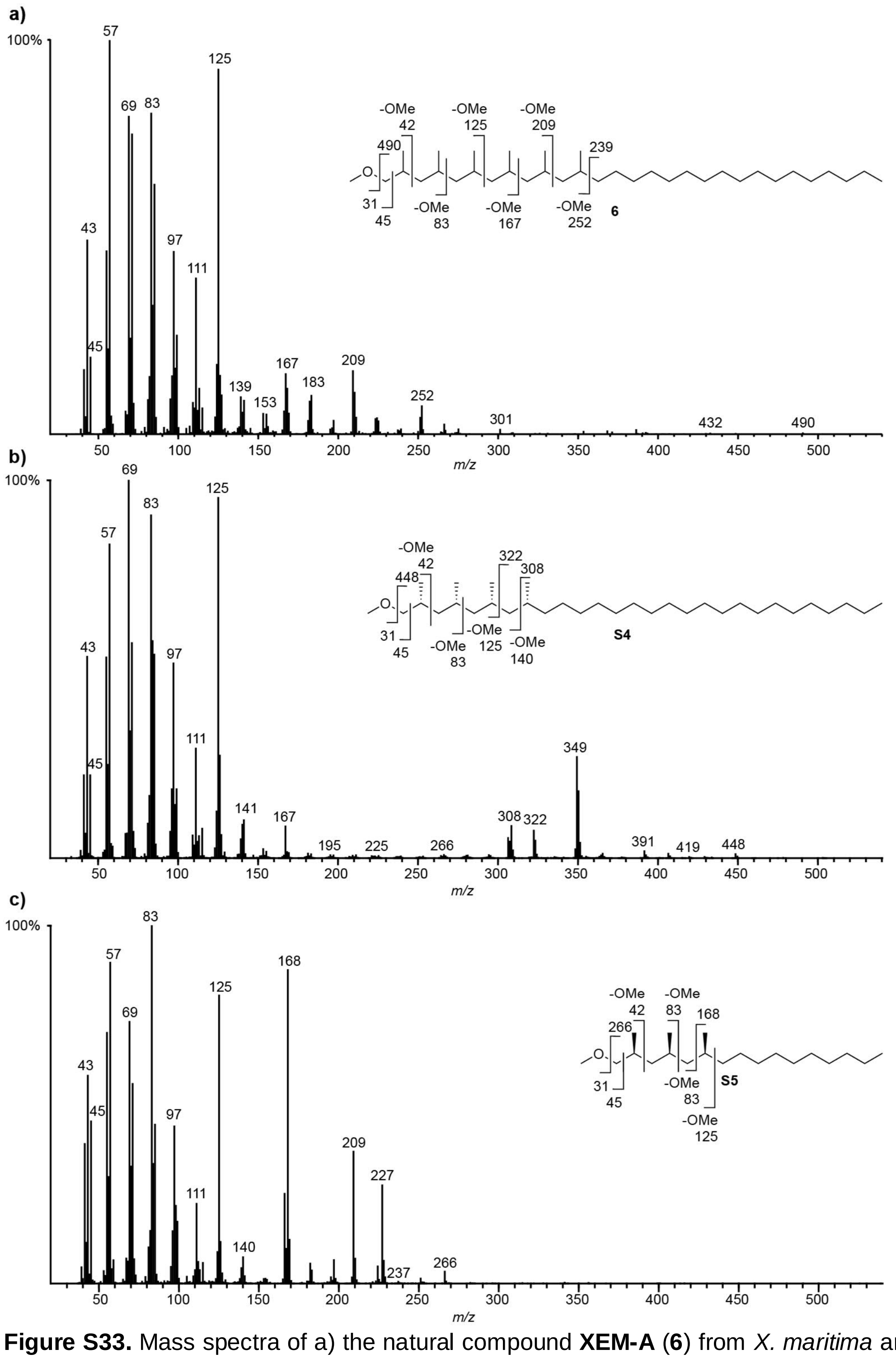

Figure S33. Mass spectra of a) the natural compound **XEM-A** (**6**) from *X. maritima* and b) **S4**, c) **S5**, as synthetic examples of similar ethers**,** related to STAR methods.

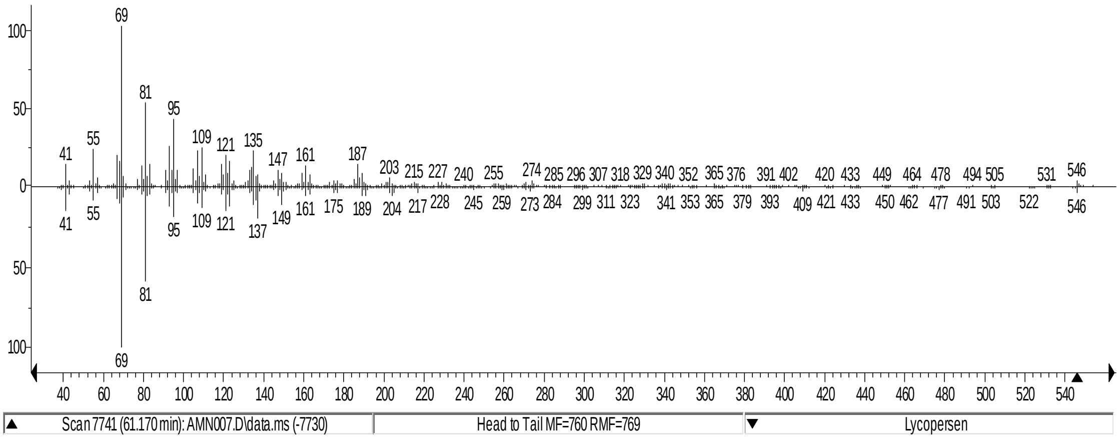

Figure S34. Mass spectra of **PFI-C** (upper part) and lycopersene (**S6**, lower part), related to STAR methods.

.

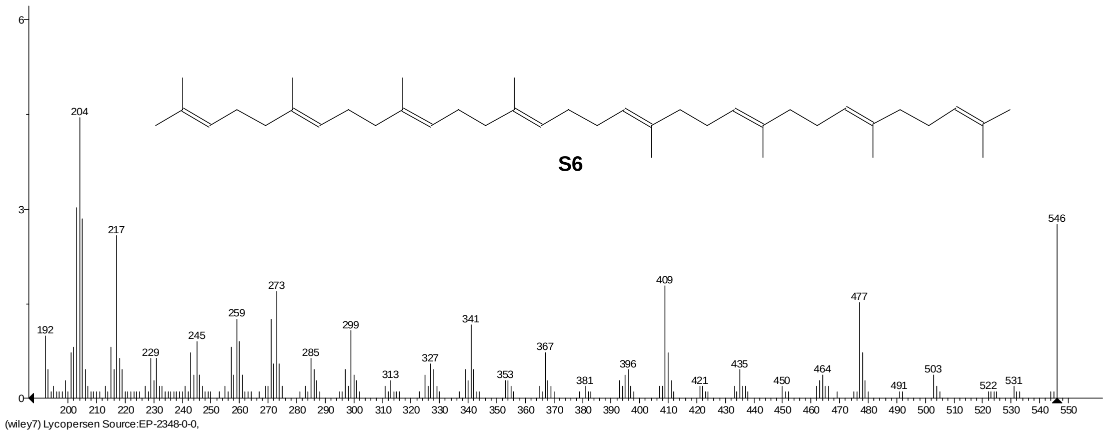

Figure S35. Mass spectrum of lycopersene (**S6**), > *m/z* 200, related to STAR methods.

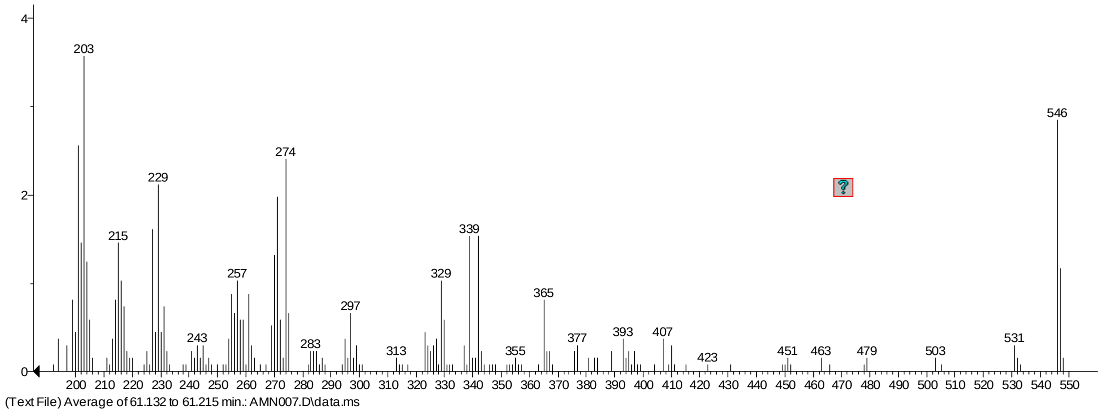

Figure S36. Mass spectrum of **PFI-C**, > *m/z* 200, related to STAR methods.

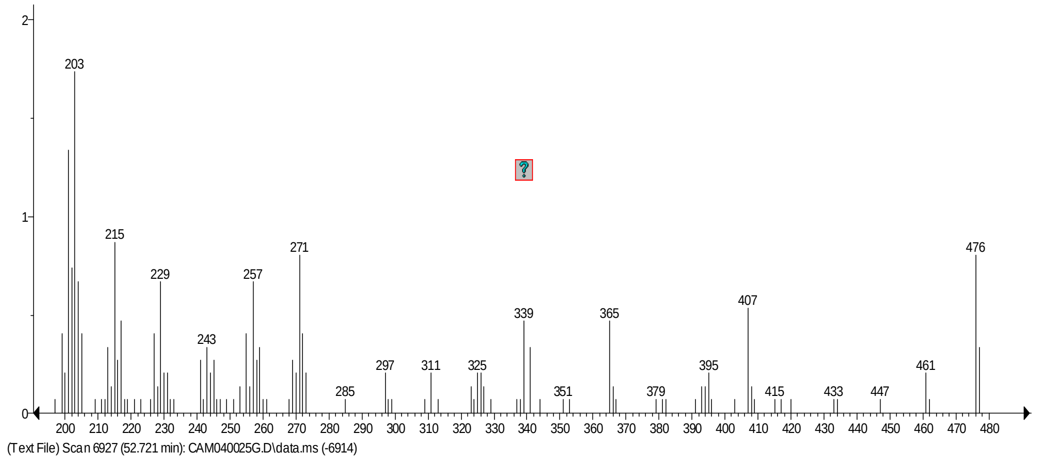

Figure S37. Mass spectra of α-geranylgeranylfarnesene (**S7**),[4] > *m/z* 200, related to STAR methods.

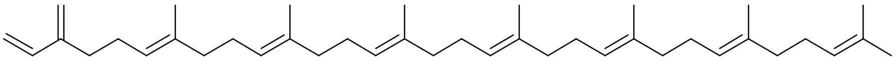

S6

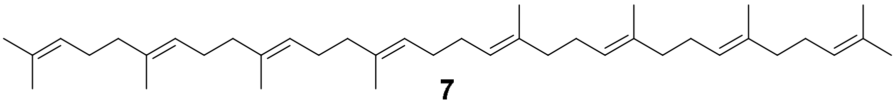

7

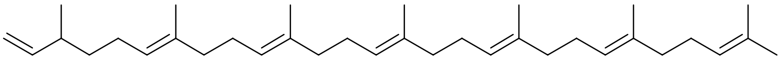

S7

Figure S38. Structures of lycopersene (**S6**), **PFI-C** (**7**), and α-geranylgeranylfarnesene (**S7**), related to STAR methods.

.

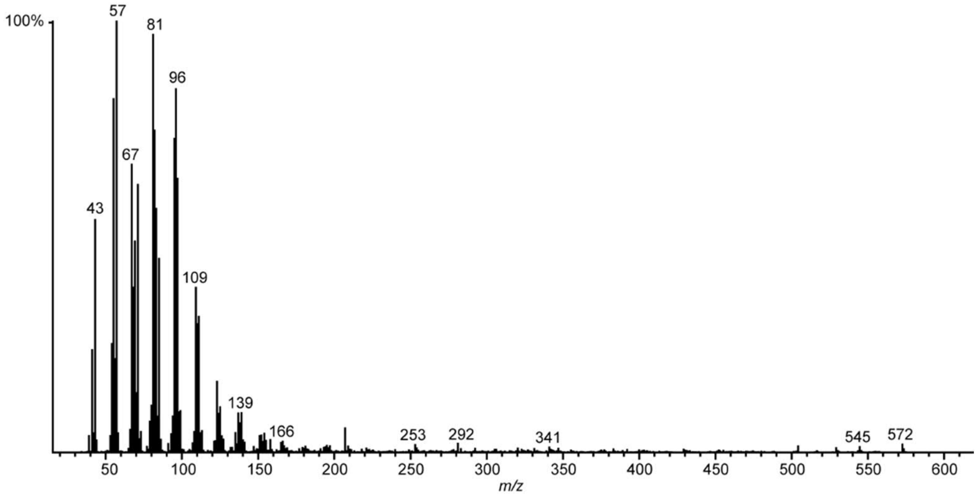

Figure S39. Mass spectrum of **ANL-B** from *A. laricis*, related to STAR methods.

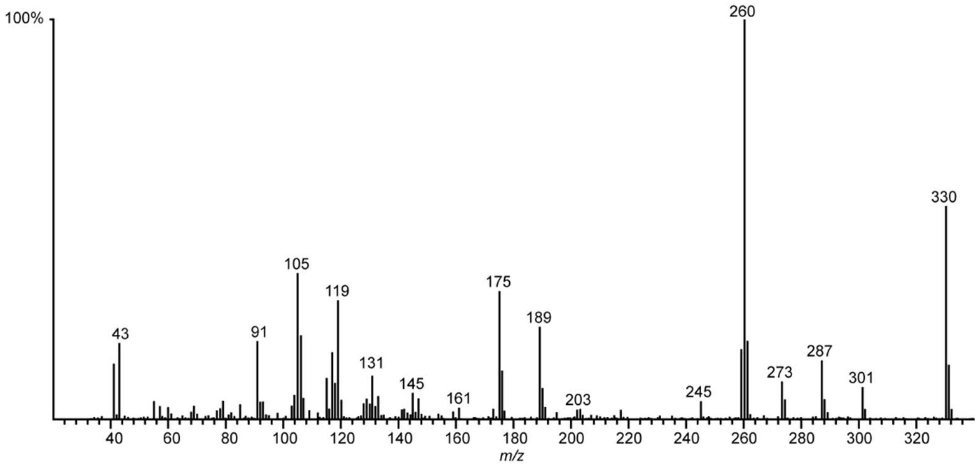

Figure S40. Mass spectrum of **FQU-A** from *F. quadrioculata*, related to STAR methods.

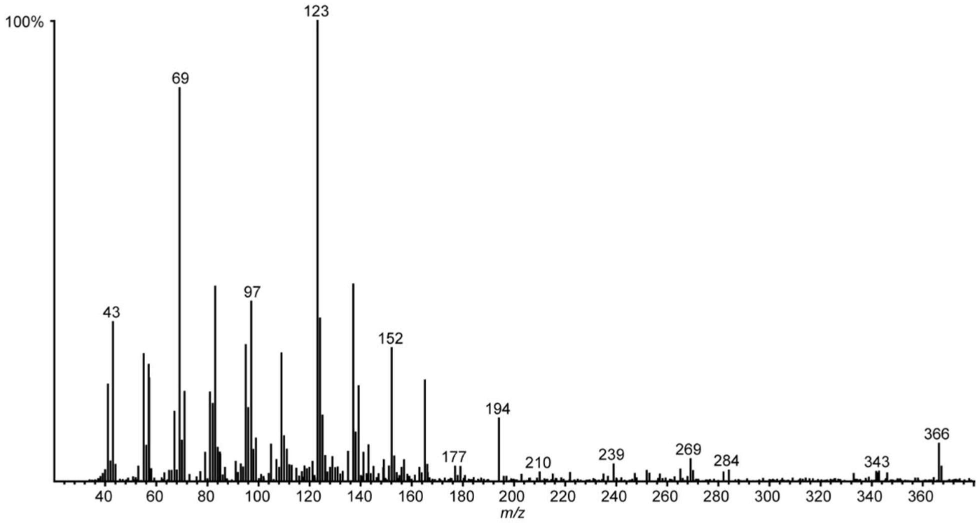

Figure S41. Mass spectrum of **FQU-D** from *F. quadrioculata*, related to STAR methods.

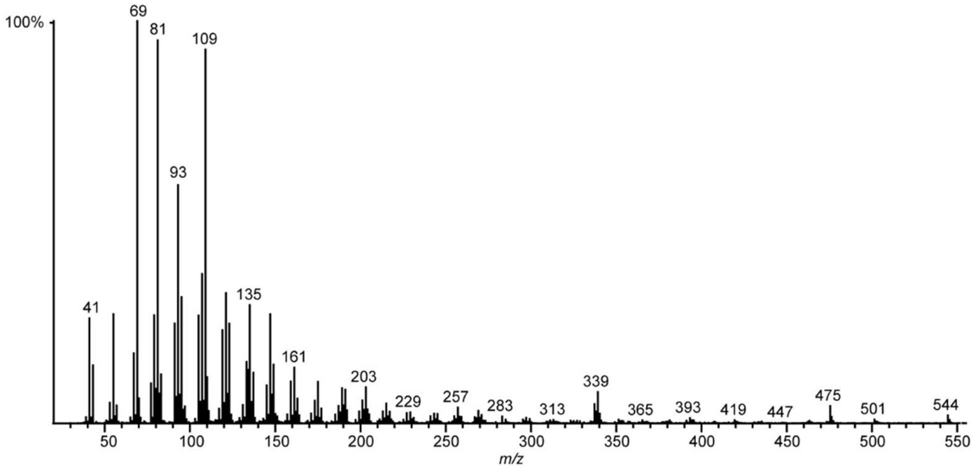

Figure S42. Mass spectrum of **FQU-E** from *F. quadrioculata*, related to STAR methods.

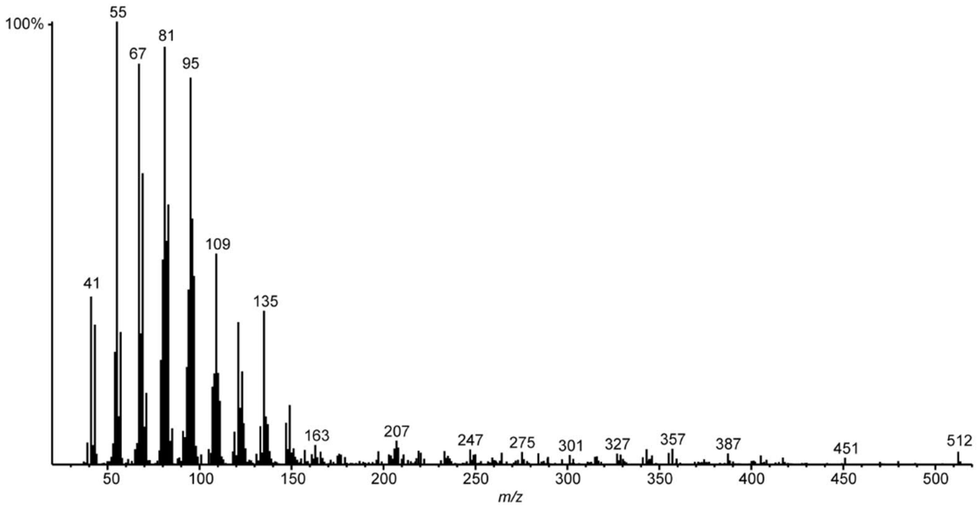

Figure S43. Mass spectrum of **FQU-G** from *F. quadrioculata*, related to STAR methods.

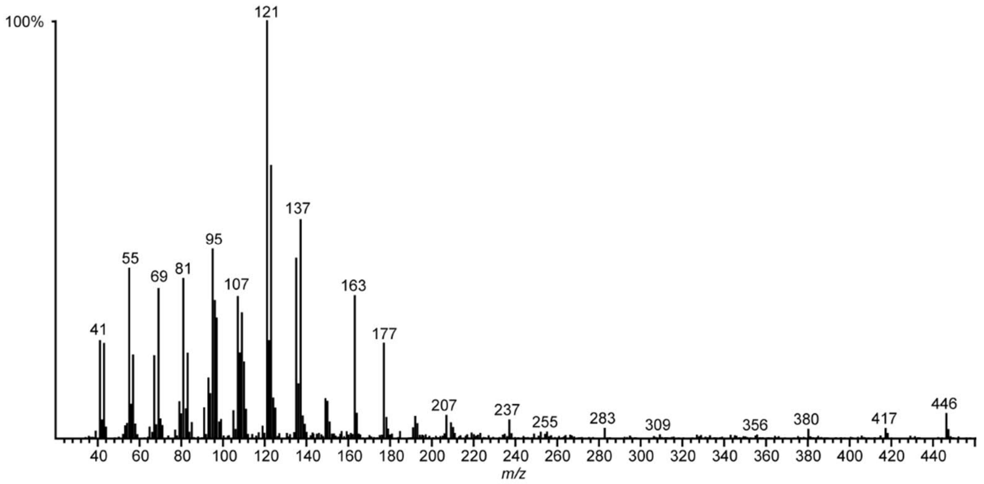

Figure S44. Mass spectrum of the **FC-A** from *F. candida*, related to STAR methods.

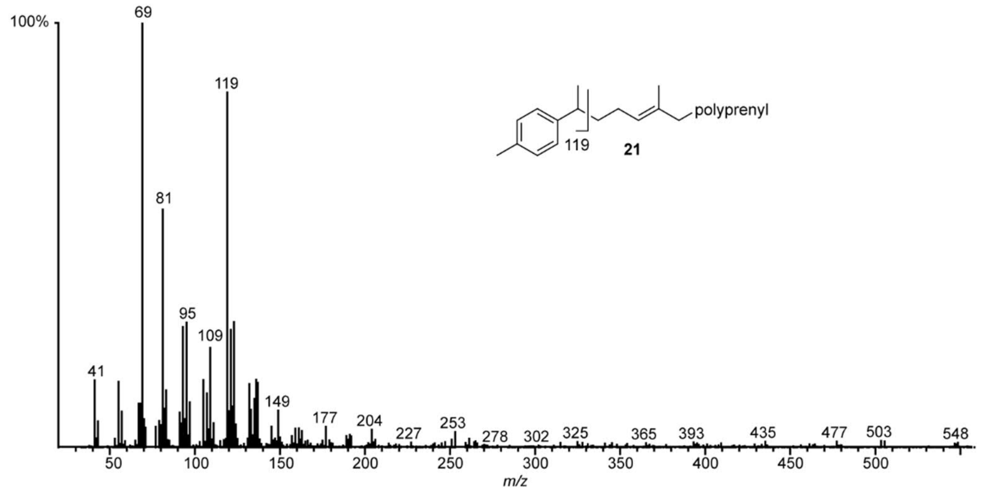

Figure S45. Mass spectrum of the **FC-D** representative for **FC-B–D** from *F. candida*, related to STAR methods.

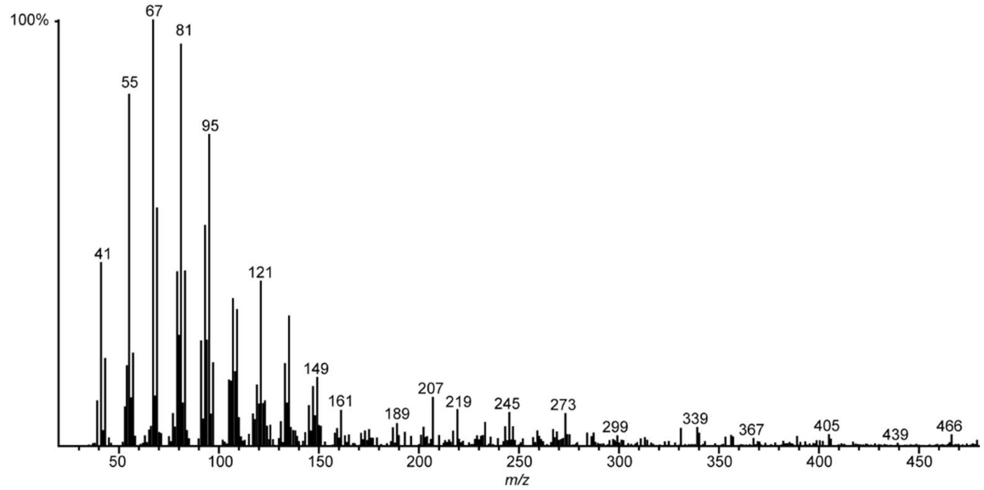

Figure S46. Mass spectrum of the natural compound **CCL-C** from *C. clavatus*, related to STAR methods.

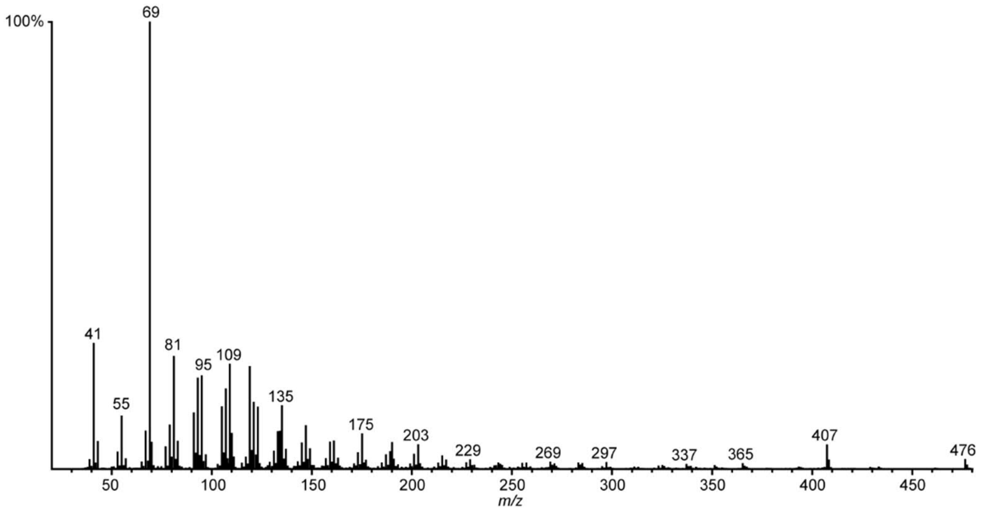

Figure S47. Mass spectrum of nitidane (**9**, **HEN-A)** from *H. nitidus*, related to STAR methods.

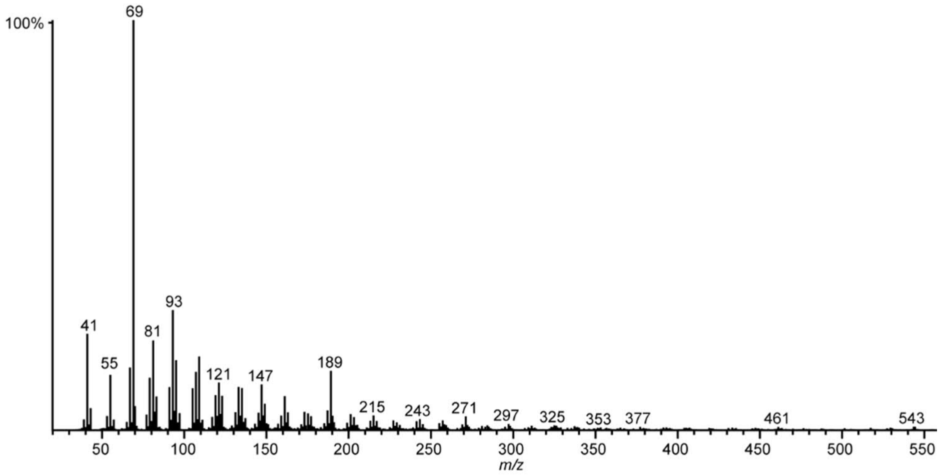

Figure S48. Mass spectrum of **HEN-B** from *H. nitidus*, related to STAR methods.

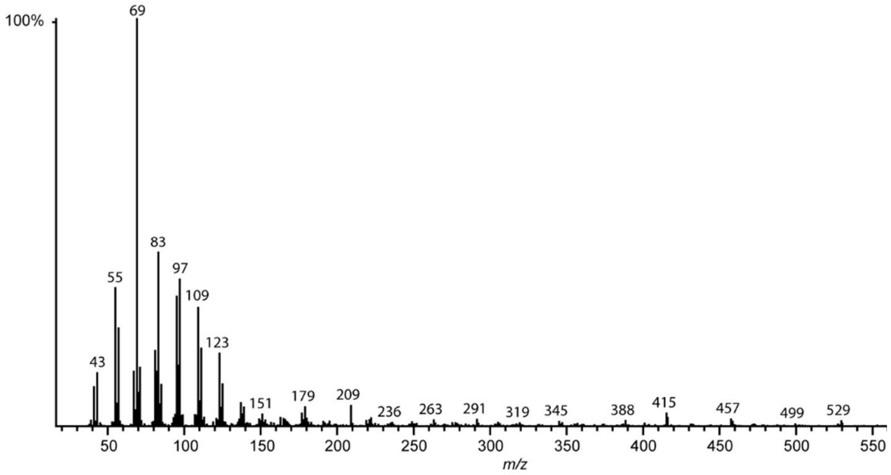

Figure S49. Mass spectrum of **HEN-C** from *H. nitidus*, related to STAR methods.

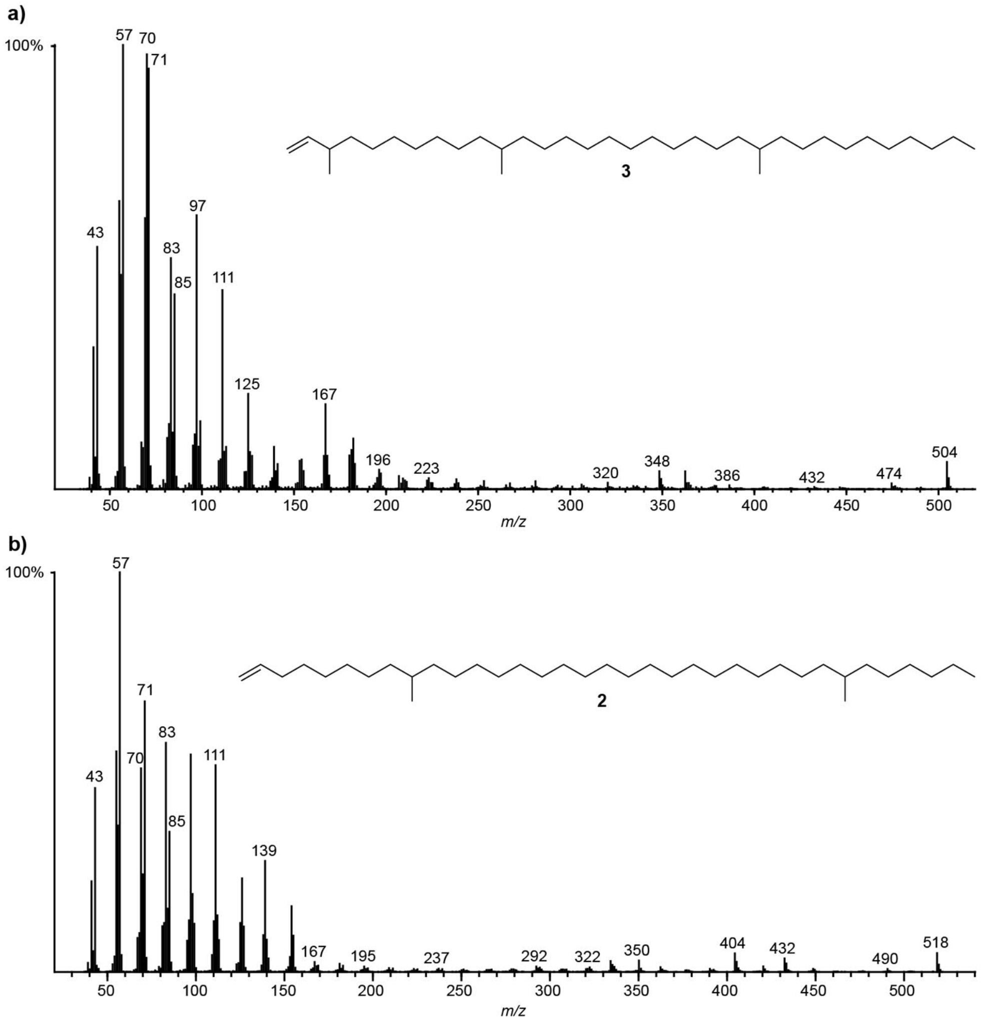

Figure S50. Mass spectra of a) **OSA-A** and b) **OSA-B** from *O. cincta*, related to STAR methods.

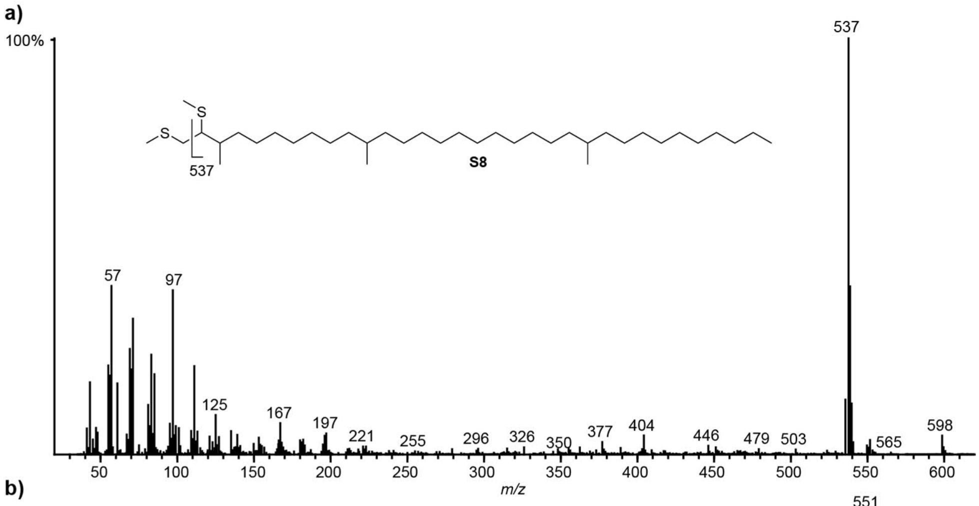

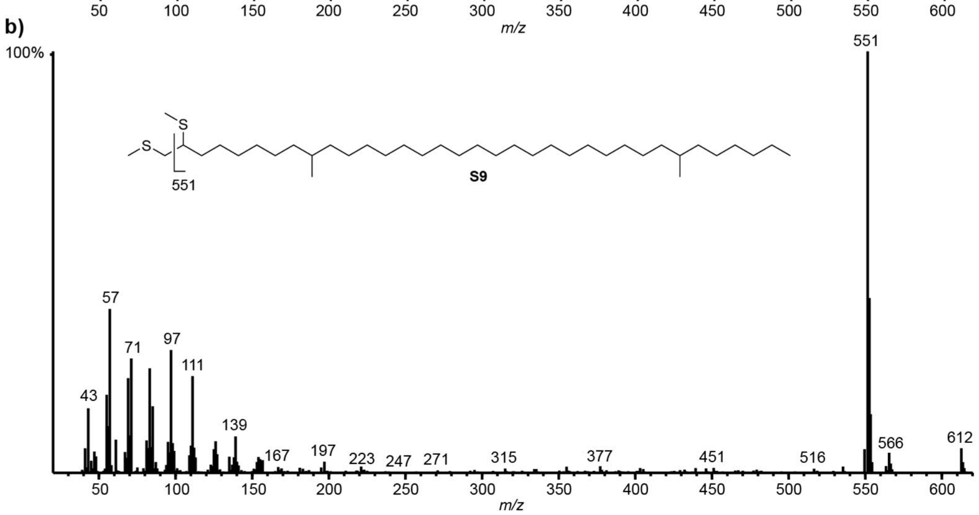

Figure S51. Mass spectra of the DMDS-derivatives of the natural compounds a) **OSA-A** and b) **OSA-B** from *O. cincta*, related to STAR methods.

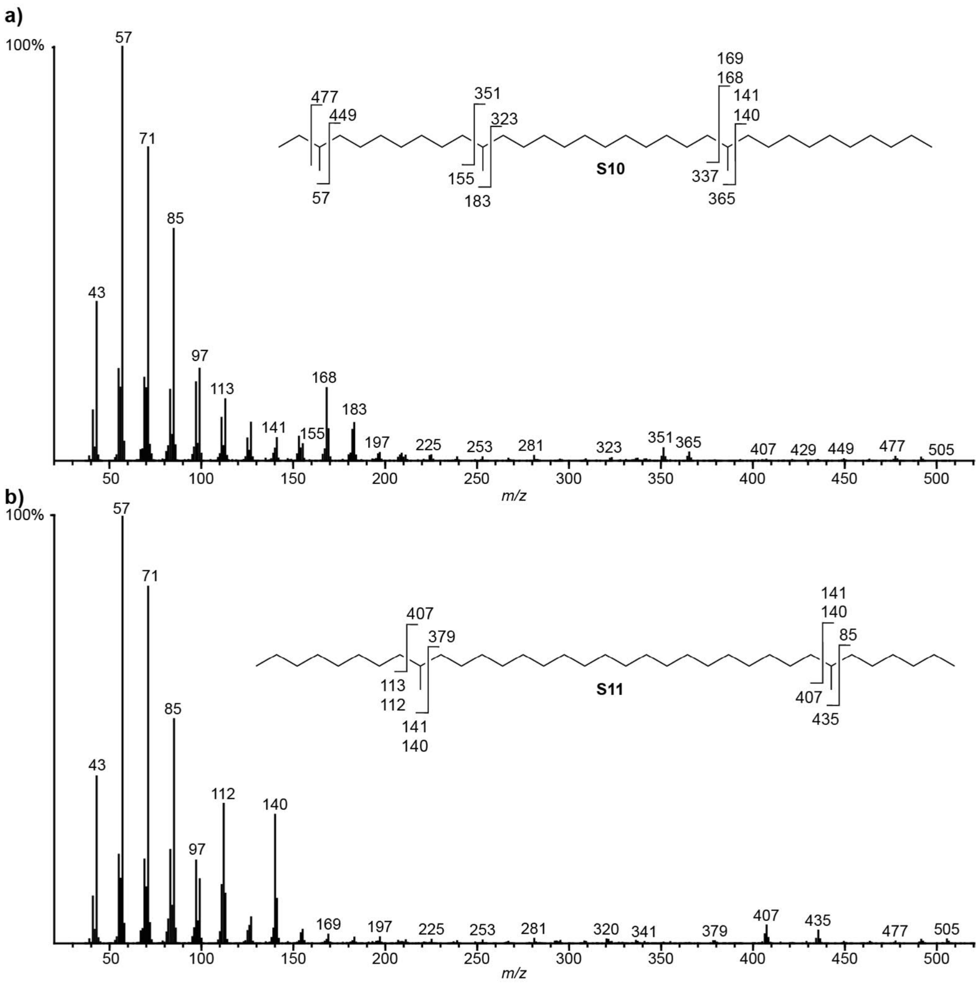

Figure S52. Mass spectra of the hydrogenated derivatives of the natural compound a) **OSA-A** and b) **OSA-B** from *O. cincta*, related to STAR methods.

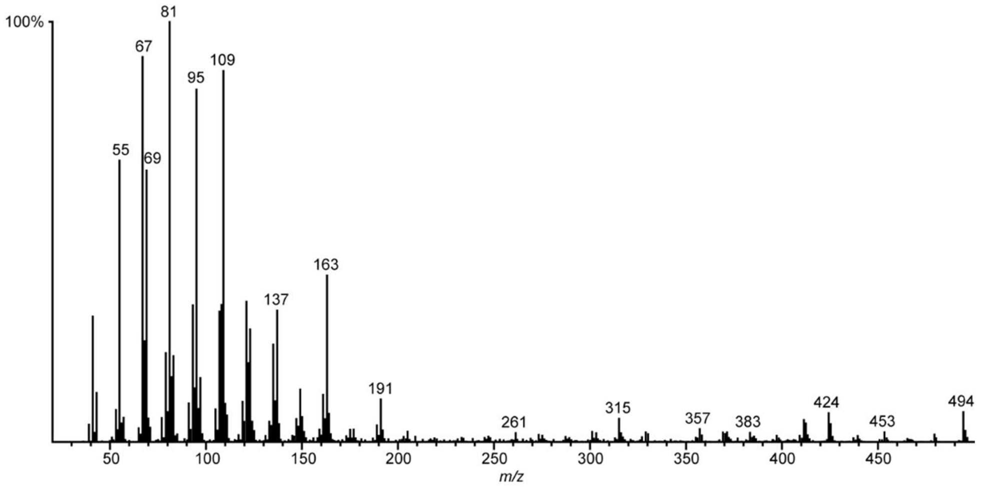

Figure S53. Mass spectrum **SIC-A** from *S. curviseta*, related to STAR methods.

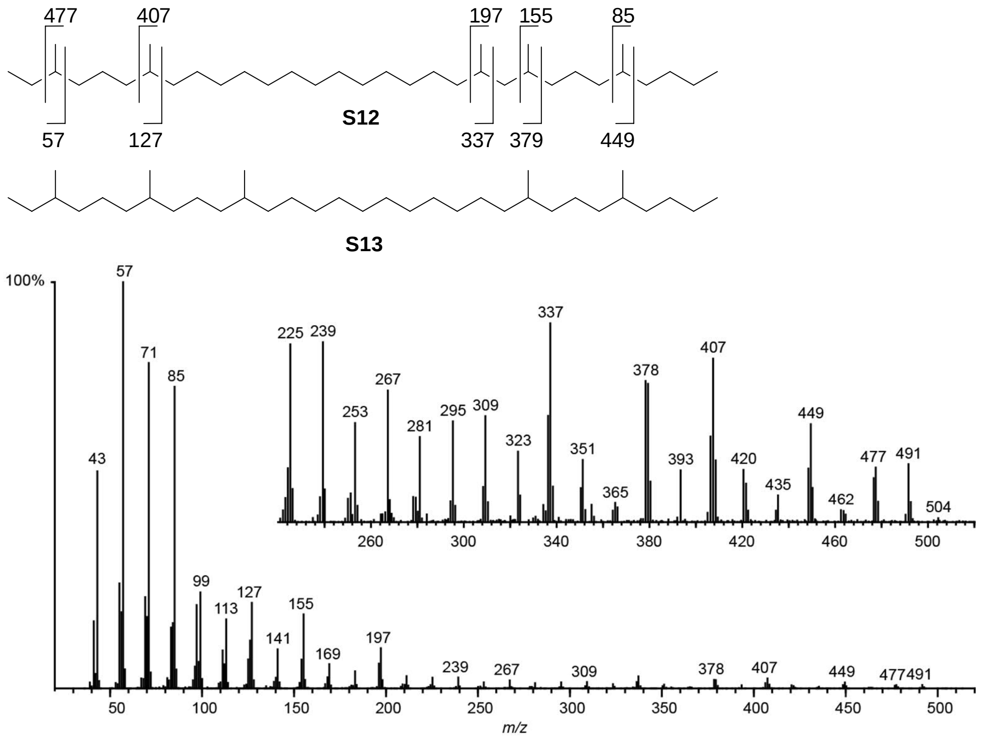

Figure S54. Mass spectrum and suggested fragmentation of the hydrogenated derivative of compound **SIC-A** from *S. curviseta*, related to STAR methods.

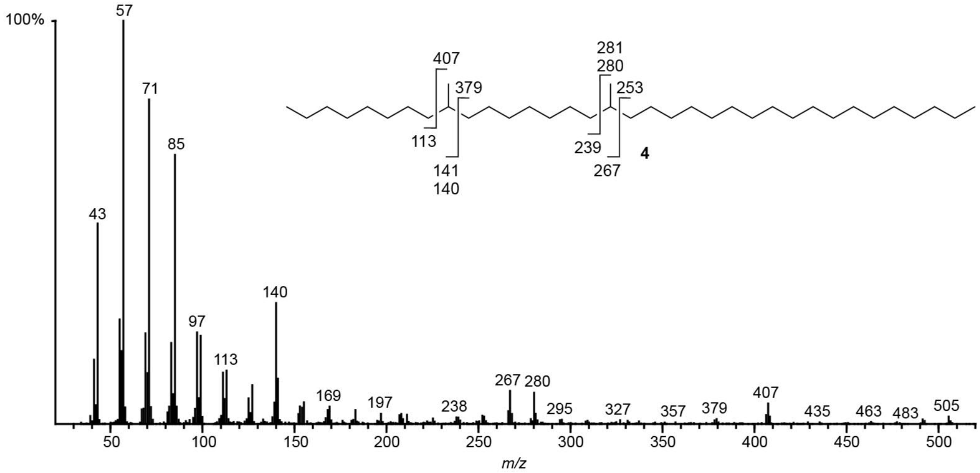

Figure S55. Mass spectrum of the **TOM-E**, identified as 9,17-dimethylpentatriacontane (**4**) by typical alkane fragmentation, related to STAR methods.

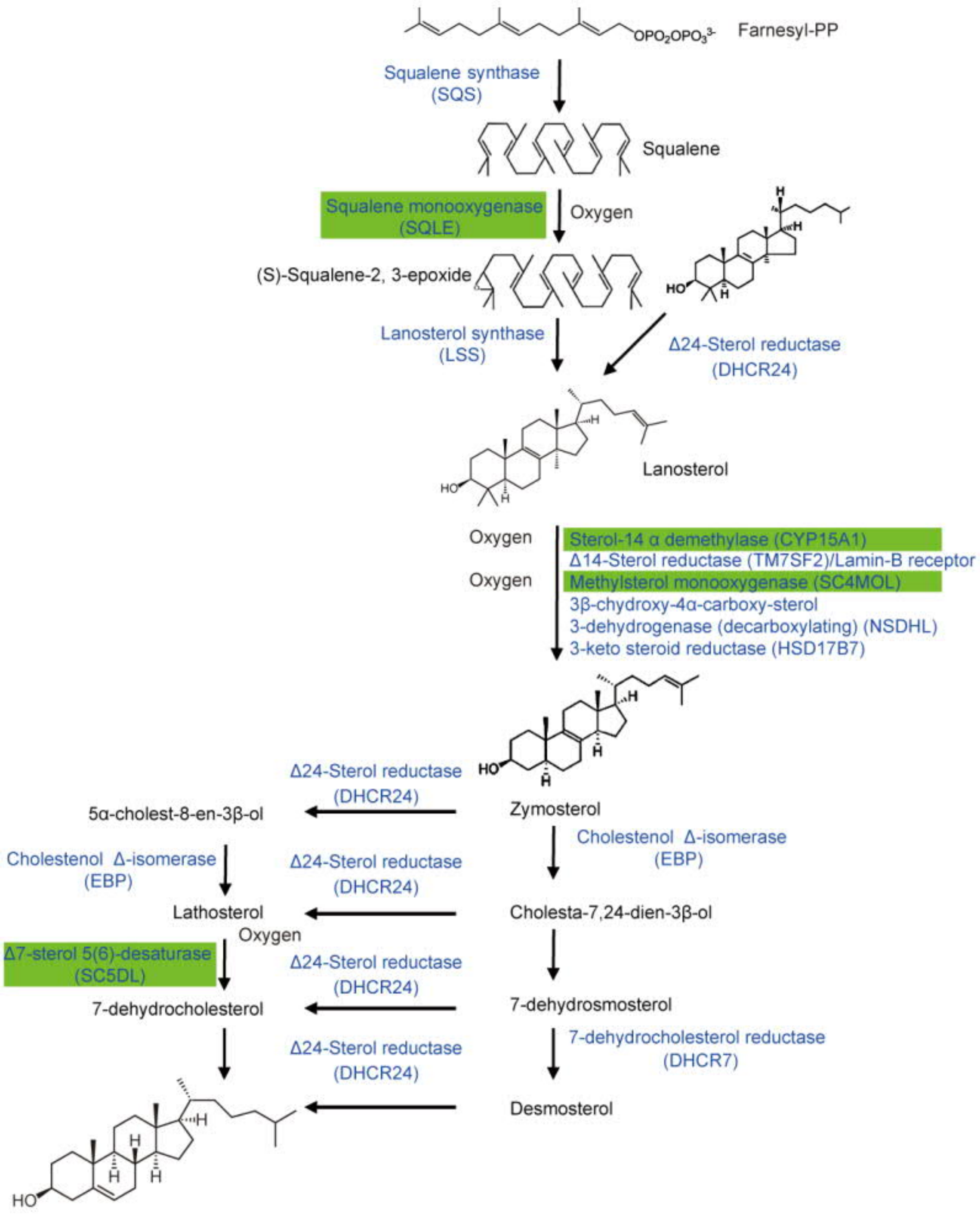

Figure S56. Biosynthetic pathway of cholesterol biosynthesis[5]. Reproduction of a Figure by Zhang *et al*., Evolution of the Cholesterol Biosynthesis Pathway in Animals. Mol. Biol. Evol. 2019 36:2548–2556 by permission of Oxford University Press. Genes in green indicate four P450 enzymes that are not relevant to our study, related to STAR methods.

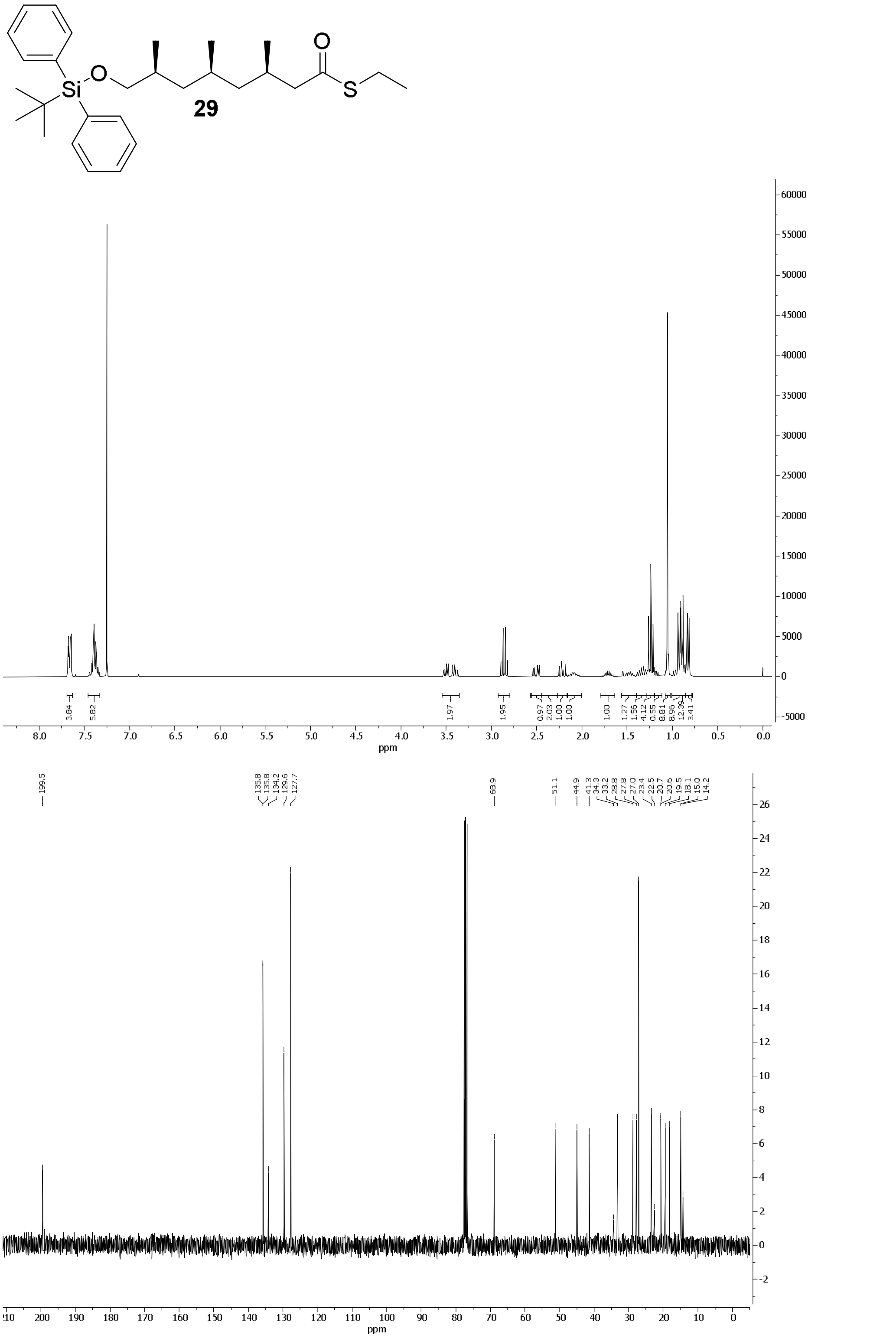

Figure S57. ^{1}H-NMR (300 MHz, $CDCl_3$) and ^{13}C-NMR (76 MHz, $CDCl_3$) spectra *S*-ethyl (3*R*,5*S*,7*S*)-8-((*tert*-butyldiphenylsilyl)oxy)-3,5,7-trimethyloctanethioate (**29**), related to STAR methods.

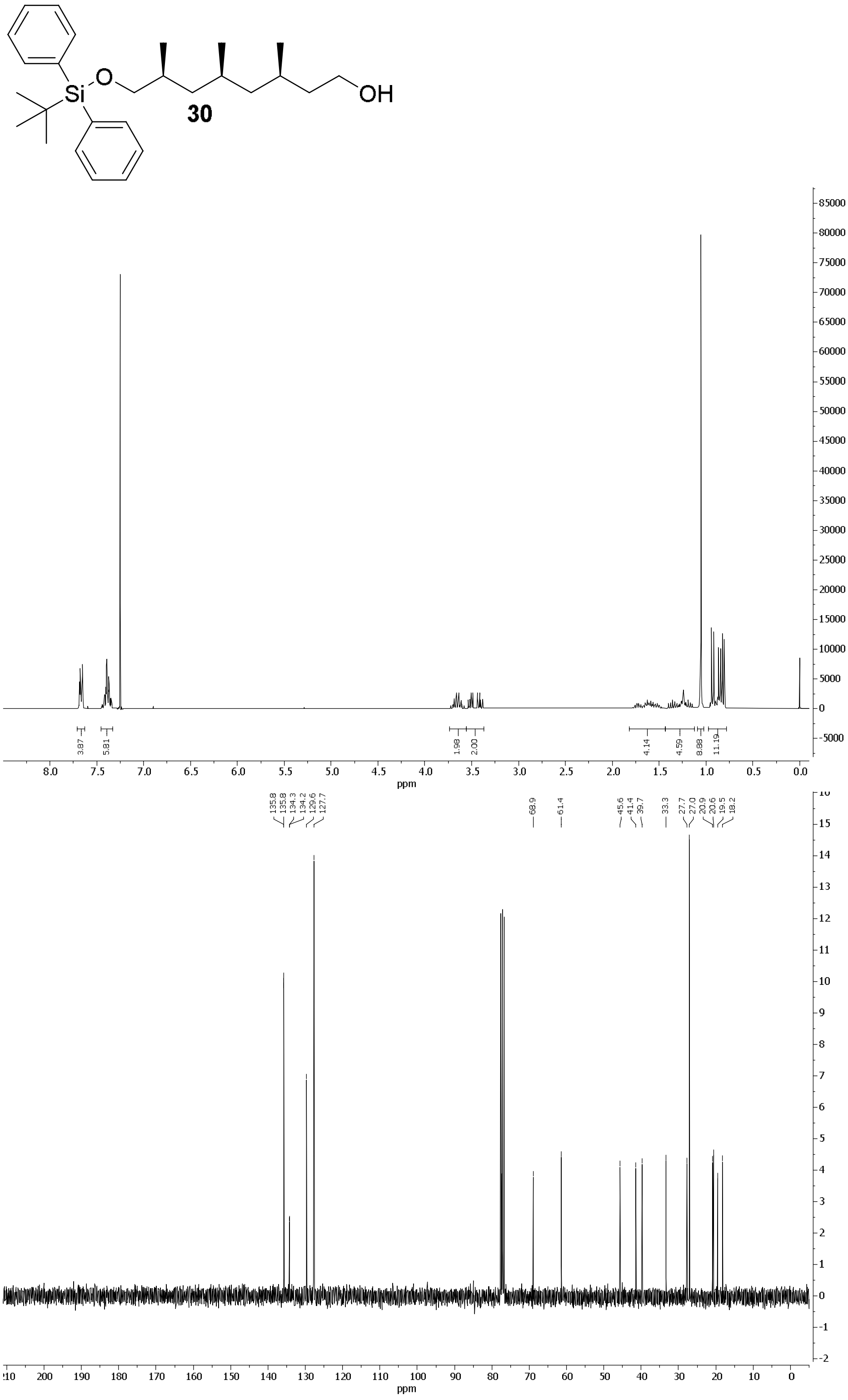

Figure S58. ^{1}H-NMR (300 MHz, $CDCl_3$) and ^{13}C-NMR (76 MHz, $CDCl_3$) spectra *S*-ethyl (3*R*,5*S*,7*S*)-8-((*tert*-butyldiphenylsilyl)oxy)-3,5,7-trimethyloctan-1-ol (**30**), related to STAR methods.

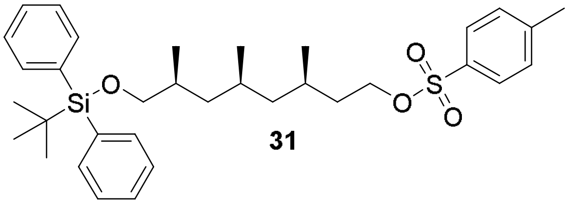

31

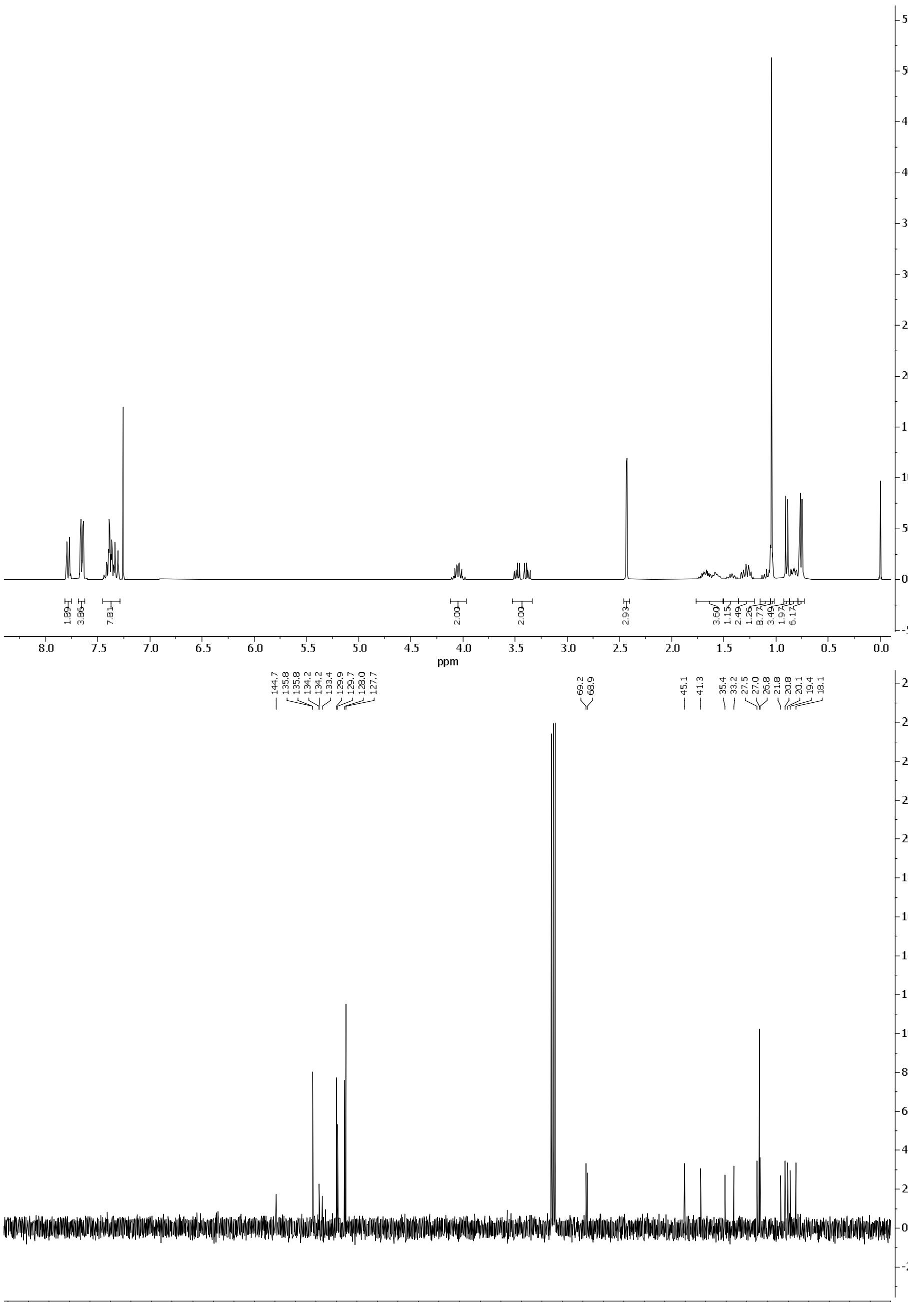

Figure S59. ^{1}H-NMR (300 MHz, $CDCl_3$) and ^{13}C-NMR (76 MHz, $CDCl_3$) spectra *S*-ethyl (3*R*,5*S*,7*S*)-8-((*tert*-butyldiphenylsilyl)oxy)-3,5,7-trimethyloctyl 4-methylbenzenesulfonate (**31**), related to STAR methods.

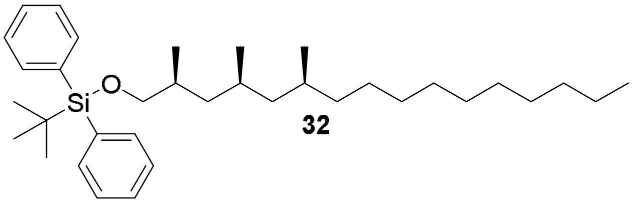

32

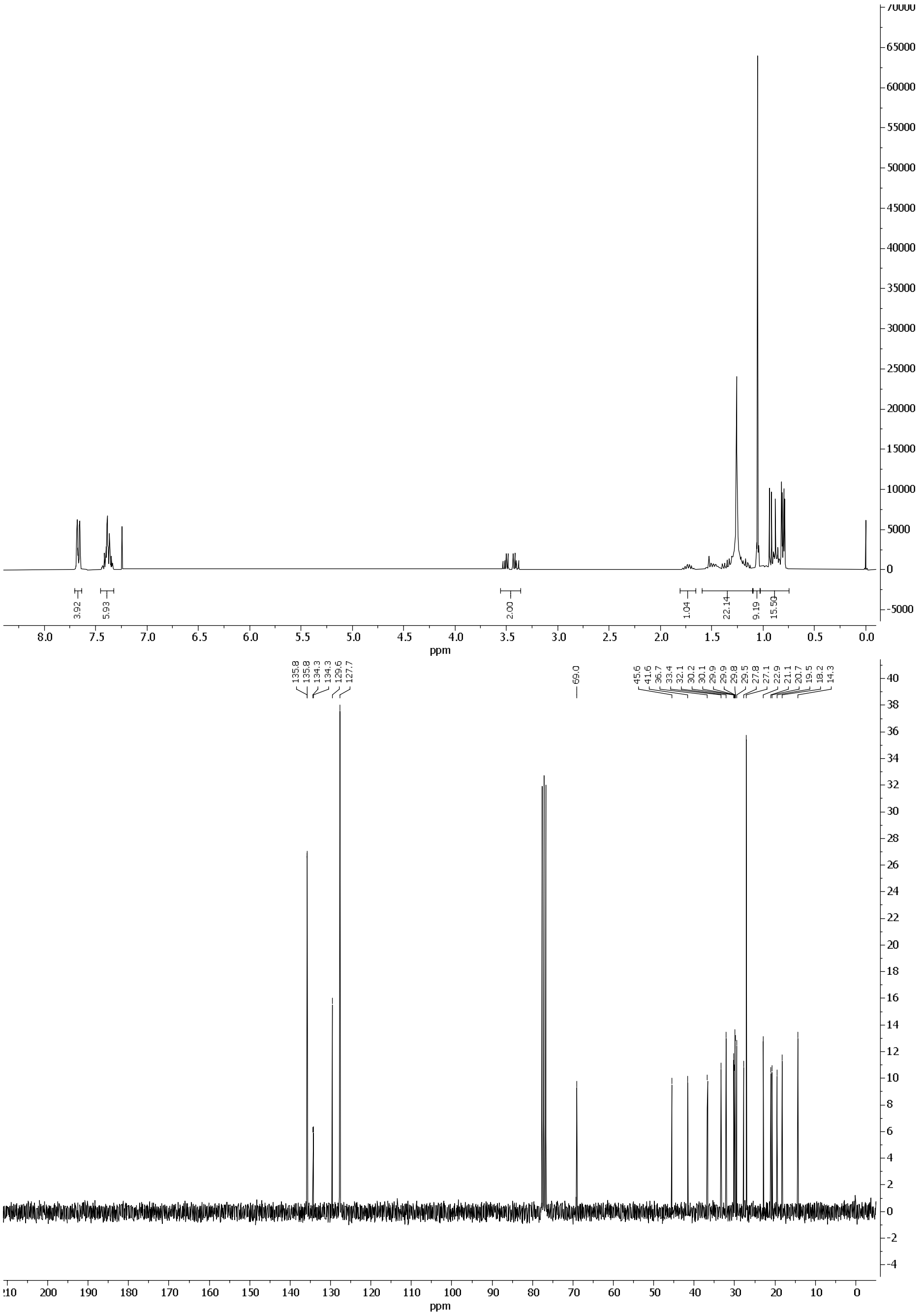

Figure S60. ^{1}H-NMR (300 MHz, $CDCl_3$) and ^{13}C-NMR (76 MHz, $CDCl_3$) spectra *tert*-butyldiphenyl(((2*S*,4*S*,6*S*)-2,4,6-trimethylhexadecyl)oxy)silane (**32**), related to STAR methods.

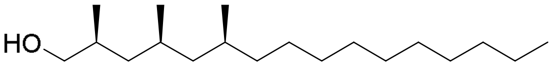

33

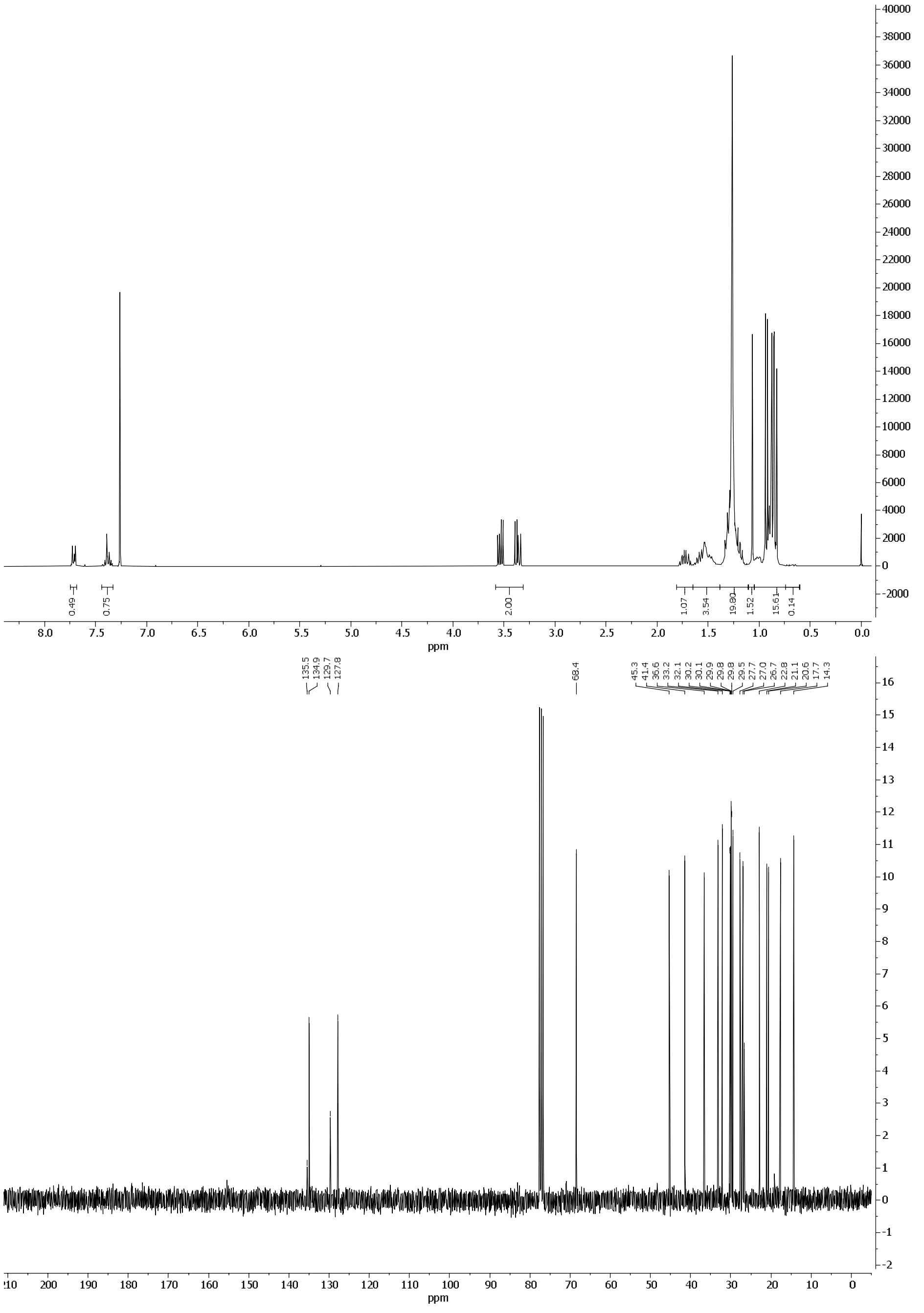

Figure S61. ^{1}H-NMR (300 MHz, $CDCl_3$) and ^{13}C-NMR (76 MHz, $CDCl_3$) spectra (2*S*,4*S*,6*S*)-2,4,6-trimethylhexadecan-1-ol (**33**), related to STAR methods.

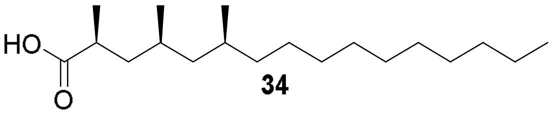

34

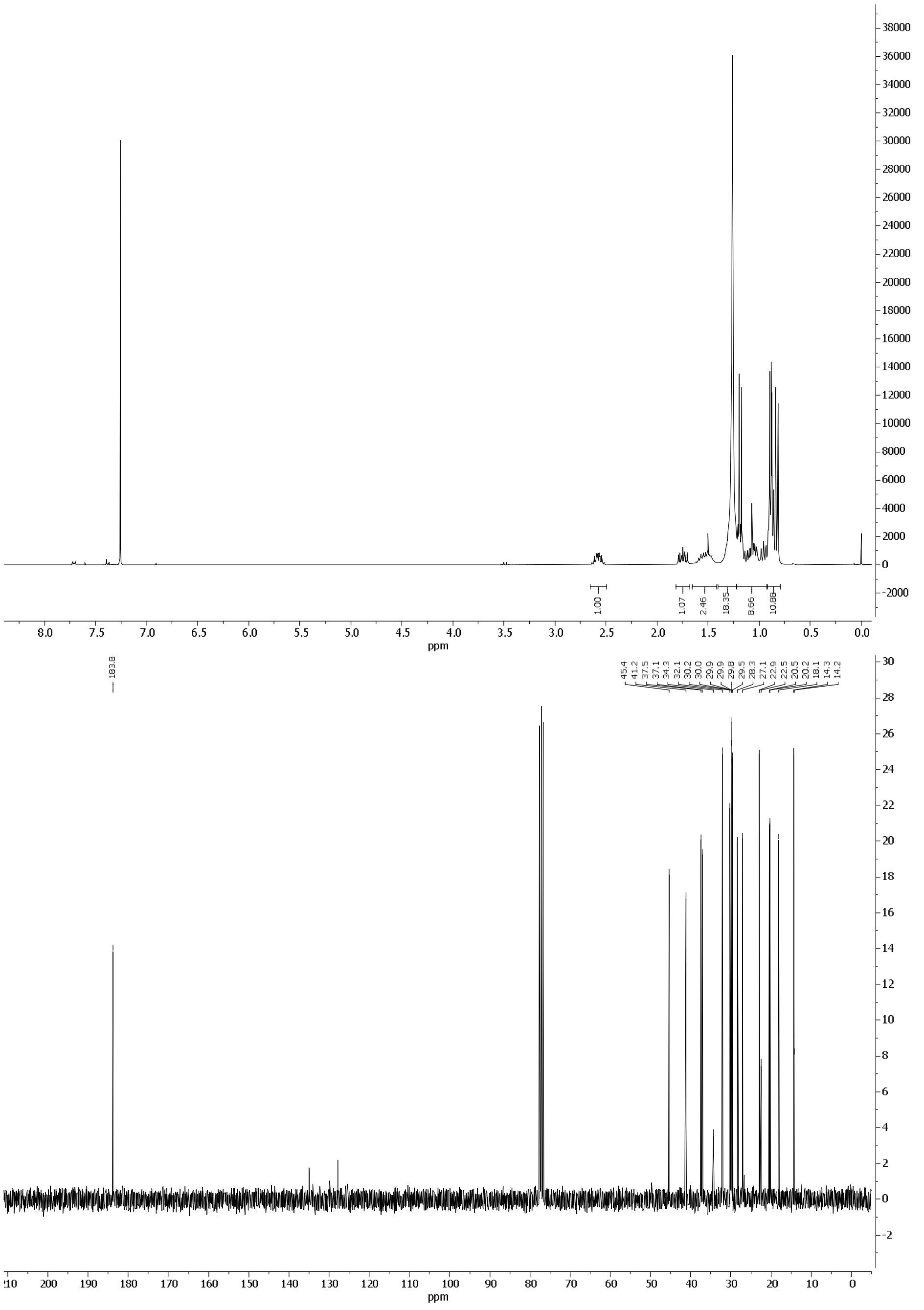

Figure S62. ^{1}H-NMR (300 MHz, $CDCl_3$) and ^{13}C-NMR (76 MHz, $CDCl_3$) spectra (2*S*,4*S*,6*S*)-2,4,6-trimethylhexadecanoic acid (**34**), related to STAR methods.

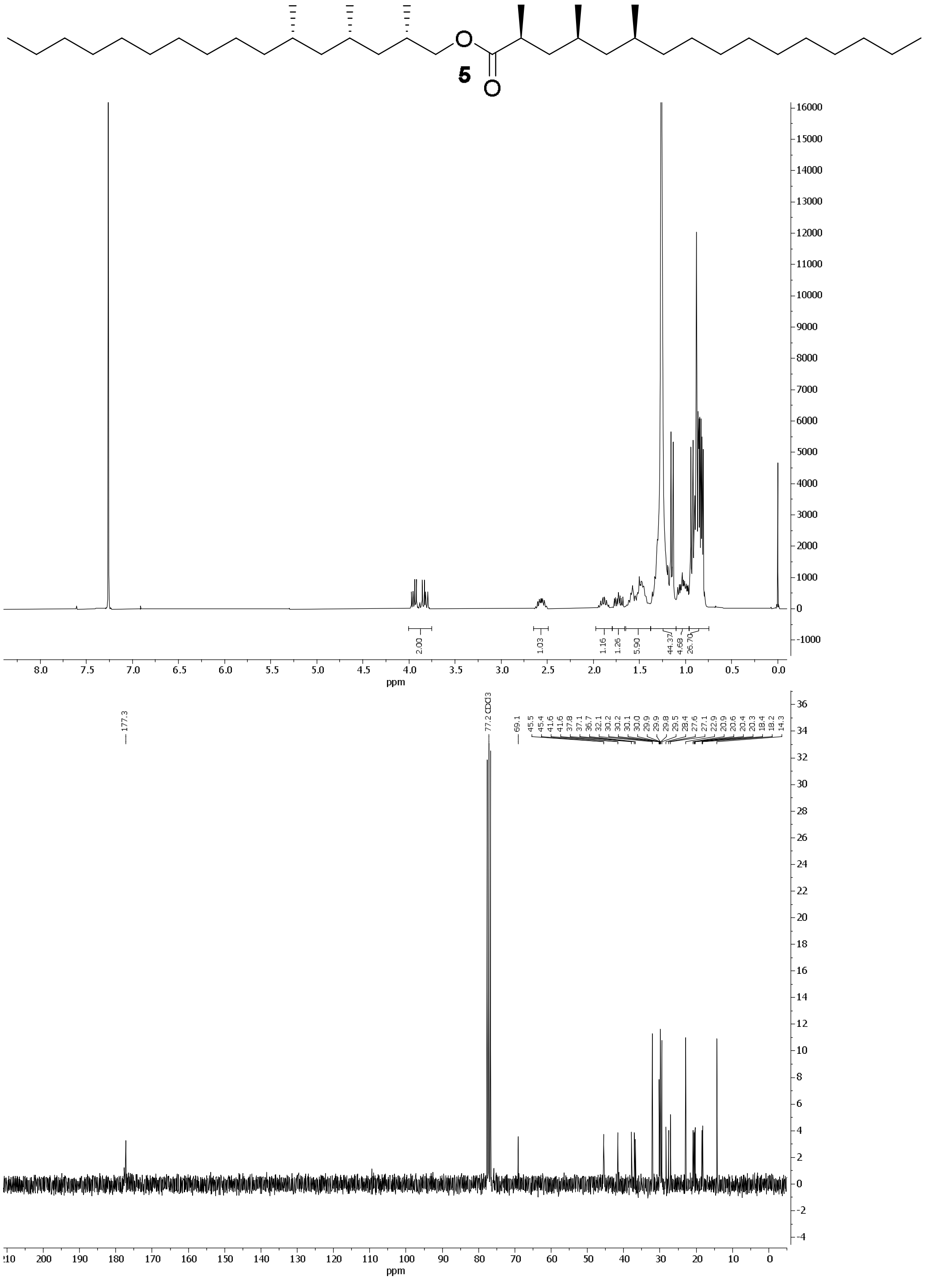

Figure S63. ^{1}H-NMR (300 MHz, $CDCl_3$) and ^{13}C-NMR (76 MHz, $CDCl_3$) spectra (2*S*,4*S*,6*S*)-2,4,6-trimethylhexadecyl (2*S*,4*S*,6*S*)-2,4,6-trimethylhexadecanoate (**5**), related to STAR methods.

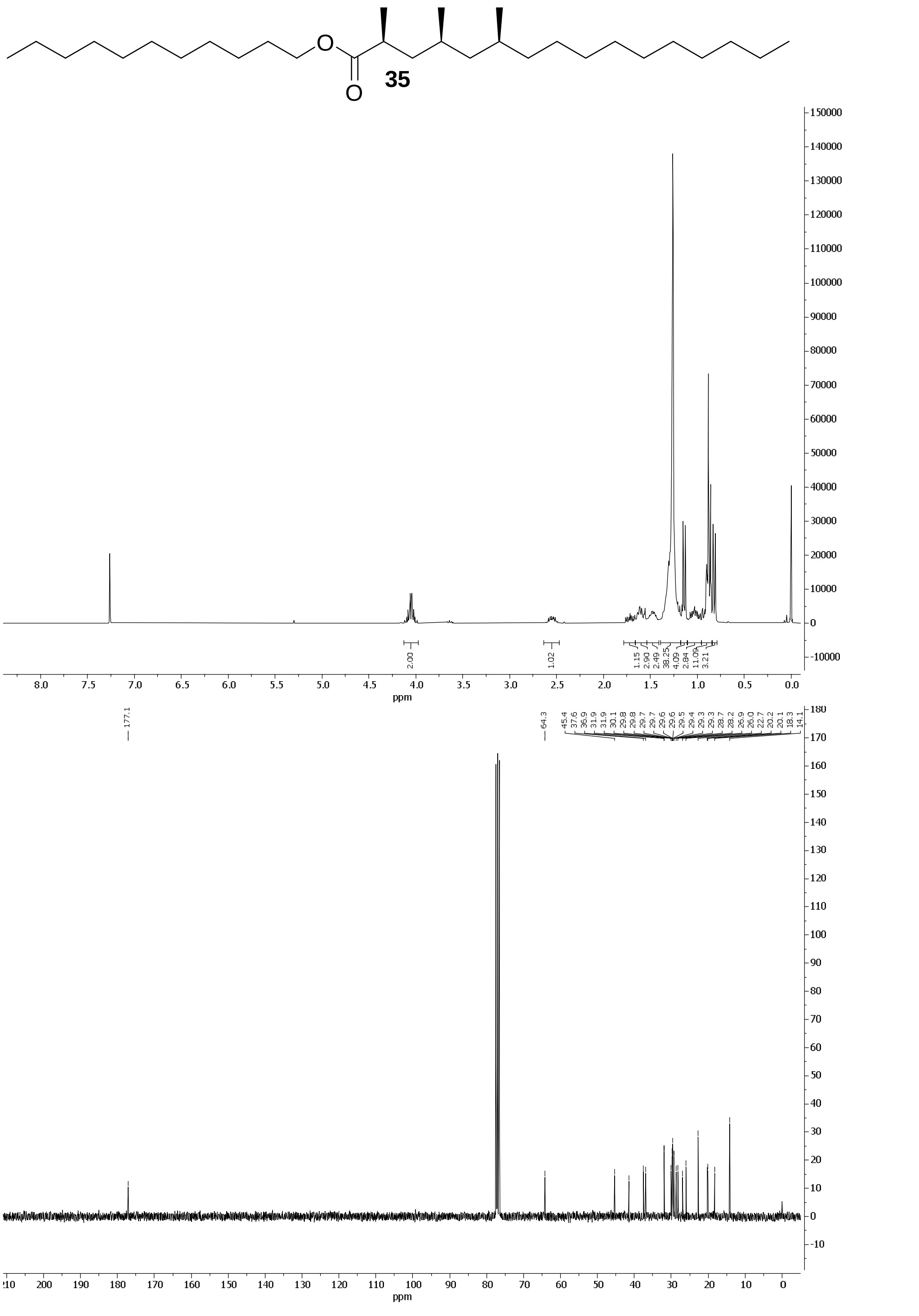

Figure S64. ^{1}H-NMR (300 MHz, $CDCl_3$) and ^{13}C-NMR (76 MHz, $CDCl_3$) spectra undecyl (2*S*,4*S*,6*S*)- 2,4,6-trimethylhexadecanoate (**35**), related to STAR methods

References

1. Stránský, K., Trka, A., Budesínský, M., and Streibl, M. (1986). Lipid compounds from the extract of the springtail *Tetradontophora bielanensis* (Waga). Collect. Czech. Chem. Commun. *51,* 948–955.
2. Nickerl, J., Tsurkan, M., Hensel, R., Neinhuis, C., and Werner, C. (2014). The multi-layered protective cuticle of Collembola. J. R. Soc. Interface *11,* 20140619. 10.1098/rsif.2014.0619.
3. Stránský, K., Zarevúcka, M., Valterová, I., and Wimmer, Z. (2006). Gas chromatographic retention data of wax esters. J. Chromatogr. A *1128,* 208–219. 10.1016/j.chroma.2006.06.035.
4. Ehlers, S., Szczerbowski, D., Harig, T., Stell, M., Hötling, S., Darragh, K., Jiggins, C.D., and Schulz, S. (2021). Identification and Composition of Clasper Scent Gland Components of the Butterfly *Heliconius erato* and Its Relation to Mimicry. ChemBioChem *22,* 3300–3313. 10.1002/cbic.202100372.
5. Zhang, T., Yuan, D., Xie, J., Lei, Y., Li, J., Fang, G., Tian, L., Liu, J., Cui, Y., and Zhang, M., et al. (2019). Evolution of the Cholesterol Biosynthesis Pathway in Animals. Mol. Biol. Evol. *36,* 2548–2556. 10.1093/molbev/msz167.
